# Implementation of Spore Display in *Paenibacillus polymyxa* with Different Hydrolytic Enzymes

**DOI:** 10.3390/microorganisms12071438

**Published:** 2024-07-16

**Authors:** Maximilian Zander, Jochen Schmid, Johannes Kabisch

**Affiliations:** 1 Institute for Molecular Microbiology and Biotechnology, University of Münster, Corrensstrasse 3, 48149 Muenster, Germany; 2 Department of Biotechnology and Food Science, NTNU Trondheim, Sem Sælandsvei 6/8, 7491 Trondheim, Norway

**Keywords:** *Paenibacillus polymyxa*, spore surface display, lipase, esterase

## Abstract

Biotechnological processes are essential for producing climate-friendly high-value chemicals or pharmaceutical compounds, which can include steps catalyzed by enzymes. Therefore, establishing new, robust, and cheap enzyme production processes is desirable. One possible way to enhance processes is through the use of the spore display method. Spore display can present heterologous proteins on the surface of bacterial spores, offering numerous advantages in a range of biotechnological applications. This study demonstrates the implementation of the spore display method in *Paenibacillus polymyxa,* achieved by modifying the spore surface, incorporating an anchoring protein, and attaching green fluorescent protein to it, allowing the visualization of fluorescent spores. Following the initial experiment, a native lipase (Lip3), a heterologous lipase (LipA) from *Bacillus subtilis*, a native esterase (PnbA) from *P. polymyxa,* and a lipoyl synthase were expressed during sporulation and displayed on the spore surface. The activity profiles were determined in the temperature range from 4 °C to 70 °C. The PnbA reached its optimum at 4 °C, whereas the LipA from *B. subtilis* showed 4.4-fold higher activity at 42 °C compared to the control. Furthermore, we explored a possible new technique for the purification of enzymes with the TEV cleavage site between the anchor and the protein of interest. Finally, we showed a not-yet-described side activity of the lipoyl synthase over a wide temperature range.

## 1. Introduction

Biotechnological methods have emerged as promising ways to effectively address pressing issues like the energy crisis and global warming in an ever-evolving global landscape characterized by increasing waste production and demand for sustainably produced chemicals [1]. Purely enzymatic process variants can outperform classical processes by maximizing substrate conversion rates and yields by accounting for losses to maintain cell metabolism. Via enzymatic catalysis, a wide range of substances can be produced efficiently under environmentally friendly conditions, such as low temperatures and atmospheric pressure, as well as in aqueous solutions [1,2,3]. However, these methods also have limitations. For example, free enzymatic biocatalysts can usually only be used once, but process variants in which the enzymes are immobilized can solve this problem [4,5,6].

The display of enzymes on the surface of a host organism, such as bacteriophages, bacteria, yeast cells, or the spore display, has emerged as a powerful tool in protein immobilization [7,8,9,10,11]. This technique offers a range of applications in biocatalysis, drug discovery, and bioremediation, allowing for the identification and optimization of enzymatic activities [12,13]. In the context of enzyme engineering strategies like directed evolution or rational design, enzyme display provides additional advantages due to a genotype–phenotype coupling [14]. It enables the rapid screening of large enzyme variant libraries, offering a high-throughput approach to enzyme optimization and selecting enzymes with specific properties for desired applications [15,16]. Consequently, enzyme display has become a valuable tool for enhancing the performance and versatility of enzymes, which play crucial roles in various industries and biotechnological fields [5,17,18]. One such display technique is autodisplay, which enables the surface expression of a protein of interest on the bacterium *Escherichia coli* by utilizing the specialized outer membrane protein AIDA-I [19]. This versatile system has applications in protein engineering, drug discovery, and vaccine development [20,21,22]. Autodisplay in *E. coli* offers notable advantages compared to other display systems, including user-friendliness, high expression levels, and the capability to display a diverse array of proteins [19]. However, this method has some limitations, such as the requirement for suitable conditions for *E. coli* growth, which may restrict the experimental setup. An alternative method is the spore surface display, in which the endospores of spore-producing bacteria are used as carriers for the immobilized proteins. Spores can withstand extreme conditions such as high temperatures, radiation, and desiccation, making them highly durable [23]. *B. subtilis* serves as a model organism in terms of sporulation and spore display, and many studies have demonstrated the efficacy of spore display in the expression of various proteins, such as enzymes, antibodies, and antigens [24,25,26]. Enzymes such as lipases, xylanases, and cellulases have been successfully presented on the spore surface, highlighting the potential of spore display for biocatalytic applications [24,27,28]. The high robustness and the genotype–phenotype preservation under the harsh conditions of this technique give major advantages [29,30,31]. An additional benefit of spore surface display is the easy recycling of the spores themselves by simple separation through, e.g., centrifugation and the reuse of the spores. Compared to conventional methods, this offers several advantages, such as lower energy consumption, lower costs, and improved sustainability, since the spores can be reused over a long period [32]. It has become a promising approach for showcasing different proteins on the outer layer of bacterial spores, generating considerable interest for its wide range of potential applications in biotechnology [24,30,33]. While the efficacy of spore surface display has been successfully demonstrated in *B. subtilis*, and its general application to *Paenibacillus polymyxa* has recently been patented, there are no publications on its application yet. This publication marks a starting point [34].

*P. polymyxa* is a Gram-positive, endospore-forming soil bacterium which is used as a biofertilizer commercially due to its ability to fix nitrogen. In addition, it has a high potential for various biotechnological applications due to its diverse metabolic capabilities and currently optimized genetic accessibility in combination with a Generally Recognized As Safe (GRAS) status for several applications [35]. Therefore, *P. polymyxa* is known for its production of a variety of extracellular enzymes, antibiotics, exopolysaccharides (EPS), and high-value-added chemicals like *R,R*-2,3-butanediol, which renders it a promising candidate for utilization in agriculture, white biotechnology, and the food industry. However, the high production yields required for an industrial process lead to the problem of product toxicity. Developing spore display in *P. polymyxa* would add a not-yet-described tool for the scientific community to its repertoire of advantages and tackle the toxicity problems (production of *R,R*-2,3-butanediol). To achieve a working spore display in *P. polymyxa*, we constructed a specific plasmid to enable the expression and display of target proteins onto the spore surface. This was accomplished by fusing the desired protein to a spore coat protein and incorporating a flexible linker between the two components [18,36,37,38,39,40]. To verify that the constructed system worked, we used GFP as a reporter system to establish the basic molecular components as proof of principle in this study. As a proof of concept, we present a native and a heterologous lipase, as well as an esterase and a lipoyl synthase on the surface of the spore. These enzymes belong to an industrially important class that catalyzes the hydrolysis of ester bonds in lipids, facilitating the breakdown of fats and oils into smaller components [41,42]. Additionally, lipases have been utilized in various industrial applications, such as food processing, detergent formulation, and biodiesel production, due to their ability to perform efficient and selective reactions under mild conditions [43]. This study thus lays the foundation for *P. polymyxa* spore display technology and can contribute to the way we harness the natural repertoire of the bacterium for a sustainable biotechnological future, with displayed enzymes paving the way for innovative and environmentally friendly industrial processes.

## 2. Material and Methods

### 2.1. Bacterial Strain and Growth Conditions

*P. polymyxa* DSM 365 was obtained from the German Collection of Microorganisms and Cell Culture (DSMZ), Germany, with the accession number CP141264. Plasmid cloning and multiplication were performed in *E. coli* Turbo (New England Biolabs, Ipswich, MA, USA). *E. coli* S17-1 (ATCC 47055) was used as a conjugative donor strain to mediate the transformation of *P. polymyxa.* The strains were cultivated in LB media (10 g/L peptone, 5 g/L yeast extract, and 5 g/L NaCl). For plate media, an additional 1.5% of agar was used. The media were supplemented with 50 μg/mL neomycin and 20 μg/L polymyxin if required. *P. polymyxa* was cultivated at 30 °C, while *E. coli* was cultivated at 37 °C unless stated otherwise. For liquid culture, the strains were cultivated in 3 mL of LB media in 13 mL culture tubes and incubated at 250 rpm. The strains were stored as cryo-cultures in 24% glycerol and kept at −80 °C for more extended storage.

### 2.2. Strain and Plasmid Construction

The plasmids used in this study use the same backbone as the pHEiP vector [44]. The backbone with the respective overhangs to the construct was amplified via PCR by use of Q5-polymerase (New England Biolabs, USA) with the pHEiP vector. The genes used here were identified by using databases like *Subti*wiki [45] and were then blasted against the *P. polymyxa* genome. The gene coding for the anchoring protein CotE and the lipase genes were amplified via Q5-PCR from genomic DNA of *P. polymyxa* DSM 365. The target proteins were fused to the anchor protein via a flexible Glycine/Serine (GGGGGSGGGGG) peptide linker. In most cases, the anchoring and fusion protein’s linker is necessary for displaying enzymes and keeping their activity [2,3]. The constructed plasmids contain a resistance gene against neomycin and an origin of replication for *E. coli* and *P. polymyxa.* GenBank files of the plasmids and strain lists are provided in the Supplementary Information.

### 2.3. Sporulation and Purification

Sporulation is frequently induced via media change or the usage of sporulation media like SG-Media [24,27]. For our studies, we used minimal media based on the M9 media with the additives described in the Material and Methods part, giving us a faster sporulation without formation of cell clumps, as frequently occurs with SG-media. Sporulation of both wild-type and mutant *P. polymyxa* strains was induced using a nutrient-deficient medium, M9. The M9 consists of M9 5 x salts (KH_2_PO_4_ 3 g/L, NaCl 0.5 g/L, Na_2_HPO_4_ 6.78 g/L, NH_4_Cl 1 g/L), glucose monohydrate 4 g/L, MgSO_4_ 1 mM, CaCl_2_ 0.1 M, Vitamin solution 2 mL/L, and trace elements solution 1 mL/L (composition in Supplementary Information). Each starter culture was inoculated with a single colony in 50 mL M9 medium in a 250 mL Erlenmeyer flask and incubated overnight at 37 °C. The main culture was then inoculated when the optical density at 600 nm (OD_600_) reached 0.1. The incubation time varied for different variants based on their respective growth rates and was individually optimized. The cells were harvested by centrifugation at 3000× *g* for 30 min, and the resulting cell pellets were resuspended in an equal volume of phosphate-buffered saline (1 × PBS, pH 7.4, composition in Supplementary Information) treated with 50 μg/mL lysozyme for 1 h at 37 °C. Finally, the harvested spores were washed twice with PBS, and their morphology was examined using an optical microscope. For rapid small-scale sporulation, agar plates could be used as follows: A 200 µL amount of a preculture grown in LB was plated on M9 minimal media (see above) agar (1.5% agar) and incubated at 30 °C for at least two days until a biofilm was observed. The number of sporulated cells was checked by light microscopy. The spore-containing biofilm was removed with a spatula, resuspended in PBS, and washed as described above.

### 2.4. Fluorescence Microscopy and Image Analysis

A 3 μL aliquot of a CotE-GFP spore suspension in PBS was dropped onto a microscope slide, covered with a coverslip, and mounted on a Zeiss Imager Fluorescent microscope (Oberkochen, Germany). A 100× phase contrast objective and a 488 nm laser line (100 ms exposure time, 3% laser) were used to image the spores. Images were exported and processed using ImageJ 1.53 (NIH) [46].

### 2.5. Cytometric Analysis

To verify the fluorescent spores and to quantify the respective number of spores per OD, the cytometer (BD Plus6) was used. A 195 μL amount of purified spore solution was mixed with 50 μL beads (Rainbow calibration 8peaks Beads, BD Bioscience, Lakes, NJ, USA) with a defined concentration of 10^7^/mL and was measured in triplicate via the cytometer. A threshold of 75,000 in the FCS scatter was set. The acquisition was stopped either when the bead number in gate R1 (respectively for the normalization and counting of the spores) reached 10,000 or after 2 min. This method was adapted from [47].

**Figure 1:**
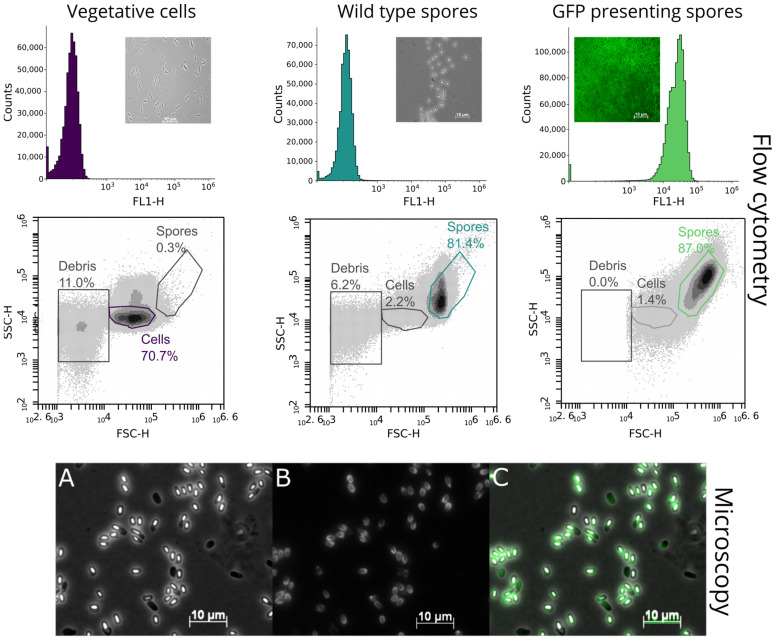
Merged graphs from left to right and top to bottom: The first row shows a cytometric analysis plotting fluorescence intensity against event count with corresponding microscopic images of wild-type vegetative cells in bright field, wild-type spores in bright field, and a fluorescence image of GFP-presenting spores. Fluorescence intensities and scatter signals of *P. polymyxa* vegetative cells, wild-type spores, and GFP-presenting spores were measured separately. In the second row, the front scatter (FSC-H) is plotted against the side scatter (SSC-H). Gates were set according to the size of the cells and spores and double-checked by microscopy. The third row shows fluorescence microscopy images of GFP-presenting spores in bright field (**A**) and in the fluorescence channel (**B**) and a merged image (**C**) showing that most of the spores showed GFP. The fluorescence (green) of each sample was excited with a 488 nm laser (100 ms exposure time, 3% laser).

Gates, indicated in in Figure 1, were set to distinguish between cells, spores, and debris while analyzing the spores with the flow cytometer. The gate for debris and for vegetative wild-type cells was defined using the scatter plot shown in the left-hand image of the second row (Figure 1), which shows a sample of a fresh overnight culture of *P. polymyxa.* In the same row, the middle plot shows purified wild-type spores, so the gate for spores was defined as the spore gate. In the scatter plots, the forward scatter (FSC-H) is plotted against the side scatter (SSC-H) to see the population differences. After setting the gates, they were fixed for every follow-up measurement to ensure comparability.

### 2.6. Hydrolysis Activity Assay

Spores with the fusion protein were harvested as described above and resuspended in Tris-HCL buffer (50 mM, pH 7.5). Lipase activity was determined using a slightly adapted *p*NPP method previously established by [48]. Each substrate, C8, C12, and C16, was prepared as a 10 mM stock solution in isopropanol. The OD_600_ of the spores in the reaction volume was set to 1 using a cuvette with a 1 cm path length, 1 mM substrate was added to a final volume of 1 mL, and the standard reaction was carried out for 1 h at 42 °C and 800 rpm agitation. In addition to these, reactions with higher temperatures were carried out with the same conditions. The reaction mix was spun down (10 min, 20,000× *g* at 20 °C) after 1 h, and 100 µL of the supernatant was loaded in triplicates on a 96-well microtiter plate ( Greiner Bio-One GmbH, Frickenhausen, Germany flat, transparent, item no.: 655101). The absorbance of the corresponding product was measured at 410 nm with a microplate reader (TECAN^®^, Männedorf, Switzerland).

The absorbance was converted into product concentration using a calibration curve (Supplementary Figure S2). The enzyme activity unit, U, was defined as the conversion of 1 µm substrate into *p*-nitrophenol in one hour at 42 °C.

### 2.7. Tobacco Etch Virus (TEV) Cleavage Assay

Cleavage activity was evaluated with spores of CotE-GFP with the TEV cleavage site between the linker and GFP. The spores were diluted to a final OD_600_ of 1 using a cuvette with 1 cm path length, and the assay was adapted and performed according to the manufacturer’s instructions (New England Biolabs, USA). After the incubation for 16 h at 30 °C, a 10 µL sample was taken and 1-to-10 diluted into 96-well black microtiter plates (GREINER flat, black, item no.: 655076). After this, the tubes were spun down (10 min, 20,000× *g* at 20 °C), and a 10 µL sample was taken and 1-to-10 diluted in the same plate. Fluorescence was measured with a microplate reader (TECAN^®^) with an excitation of 485 nm and an emission of 510 nm with a gain of 115.

### 2.8. Recycling of Spore Activity

Every recycling run was performed as described in the Material and Methods part (Hydrolysis Activity Assay). For the repeated, continuous usage of the spores, centrifugation (10 min, 20,000× *g* at 20 °C) of the used recombinant (CotE-Lip3, CotE-PnbA, CotE-LipA, and CotE-LipoylA) spore, washing with PBS buffer, and resuspension into a newly prepared substrate containing reaction buffer were performed.

### 2.9. Statistical Analysis

All experiment data were plotted with GraphPad PRISM 5 with the ±standard error of the mean. All assays were performed for at least three replicates. Statistical significance and *p* values were derived from two-tailed *t*-tests.

## 3. Results

### 3.1. Construction of Strains and Plasmids for the Expression of Fusion Proteins on the Spore Surface

The spore surface display was designed based on the previous knowledge obtained from *B. subtilis* spore display approaches [24,30,33]. The display system includes an anchoring protein located in the coat of the spore, a linker for flexibility and distance, and the displayed protein. By using the database *Subti*Wiki [45], we identified anchoring proteins known from *B. subtilis* and performed a blastX-search (a Blosum80 matrix with a max e value of 0.1 was used) using the *B. subtilis* protein sequences against the genome of *P. polymyxa* DSM 365 [49,50,51]. By that approach, we could identify *cotE* as the only *cot* gene identified in *P. polymyxa* [52]. CotE plays a significant role in assembling the outermost spore layer, called the crust, of *B. subtilis*, and the homolog protein from *P. polymyxa* showed an E value of 9.47 × 10^−38^ and a query coverage of 71.27 %.

First, GFP was chosen for the proof of principle as it gives a readily measurable output. A His-Tag was added to the C-terminus of the fusion protein for future purification purposes. The medium-high copy number pHEiP shuttle vector, already developed for *P. polymyxa,* was chosen as a backbone [53,54]. The construct was designed with the native promoter of *cotE* from *P. polymyxa*, *P_cotE_,* to synchronize the spore formation with the expression of the fusion protein and, hence, the incorporation into the spore’s crust layer. For the native promoter 500 bp upstream, the *cotE* gene was selected as the putative promoter region. An exemplary schematic design of the spore display system used here can be found in the Supplementary Information.

In addition to the CotE-GFP spore display, we created a spore display with a native lipase, an esterase from *P. polymyxa*, and a heterologous lipase from *B. subtilis*. In the literature, spore display was already performed in *B. subtilis* with its native lipase A [27]. We blasted for lipases known from *B. subtilis* in the *P. polymyxa* genome. No analog to lipase A (P37957) was found in the *P. polymyxa* genome. We thus used the *lipA* sequence from *B. subtilis* and used it for the spore display as a heterologous gene. The bioinformatic search provided us with a monoglyceride lipase (Lip3, NF024107.3) as well as a para-nitrobenzyl esterase (PnbA, P37967), which were chosen for the display of two native enzymes from *P. polymyxa*. Assay parameters were chosen as described for LipA from *B. subtilis* [28]. *P. polymyxa* strains were transformed with corresponding plasmids verified through DNA sequencing for spore production. All used oligonucleotides can be found in the Supplementary Information (Table S3).

### 3.2. Identification of the Green Fluorescent Protein Display via Fluorescence Microscopy and Flow Cytometry

The proof of function is shown in Figure 1. The merged image of purified and washed spores shows a signal in the channel corresponding to green fluorescence. The results indicate that the chosen expression system and the fusion protein are correctly expressed during sporulation and that GFP is bound to the spore surface. Cytometric analysis was performed to verify fluorescence on the spores, as shown in Figure 1 as well.

Figure 1 shows flow cytometric measurements as well as microscopic images of spores displaying GFP. The cytometric analysis shows that the wild-type cells and spores do not exhibit fluorescence signals in the FL1-H channel plotted against the event count compared to fluorescent spores in comparison to the GFP-displaying spores. This is underlined by the included microscopy images of the three cell types. The second row in the cytometric analysis shows the front scatter (FCS-H channel) plotted against the side scatter (SSC-H channel). The gates for vegetative cells and spores were set using fresh cells and purified spores. The wild-type vegetative cells showed no spores and 11% debris. Purified wild-type spores as a control showed 2.2% residual cells and 6% debris. The purified GFP-presenting spores showed 1.4% residual cells, and the gate for debris was previously cleared by the set threshold. The last row of images represents microscopic images taken using different channels of GFP-displaying spores with distinct fluorescence signals of the spores. These results indicate that the expression system is active, as verified via flow cytometry and fluorescence microscopy.

To obtain a measure of the used biocatalyst, correlation of the used OD_600_ to the number of spores per mL was performed by a calibration curve with different ODs which were measured using flow cytometry (Supplementary Figure S1).

**Figure 2:**
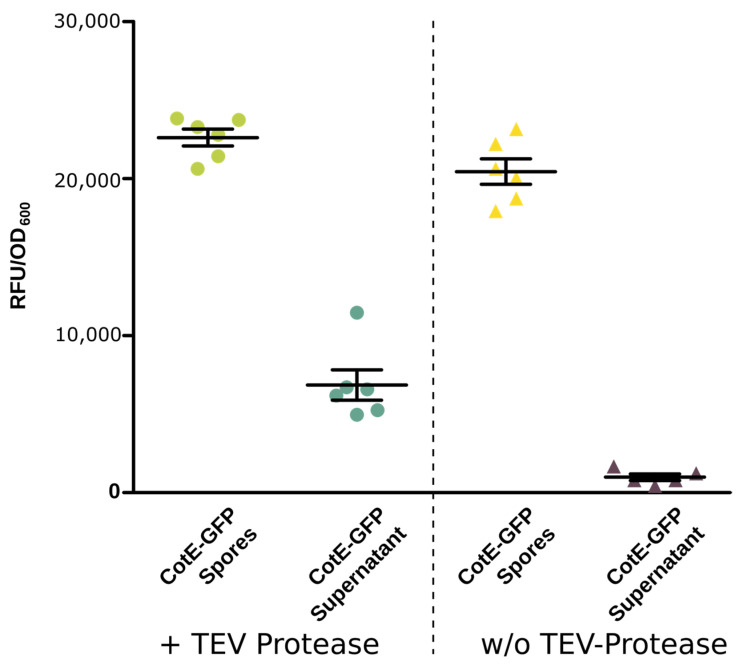
Dot plot of the TEV cleavage assay with (+TEV-Protease) and without (w/o TEV-Protease) adding TEV protease with the same conditions. Spores carrying the CotE-GFP with the TEV cleavage site were used in this experiment. TEV supernatant indicates the supernatant harvested after the TEV cleavage assay, as described in the Materials and Methods section (n = 6).

For further investigations of the spore display, we incorporated a TEV cleavage site [55] between the linker and the protein of interest, here, GFP (Supplementary Figure S8, showing where the TEV recognition site is located). The results in Figure 2 show a difference between the untreated and treated supernatant, indicating that the TEV cleavage site is accessible for the protease. Consequently, we showed that at least a part of the GFP was cleaved.

### 3.3. Enzyme Activity Assays of the CotE-Lip3, CotE-LipA, and CotE-PnbA Fusion Proteins

**Figure 3:**
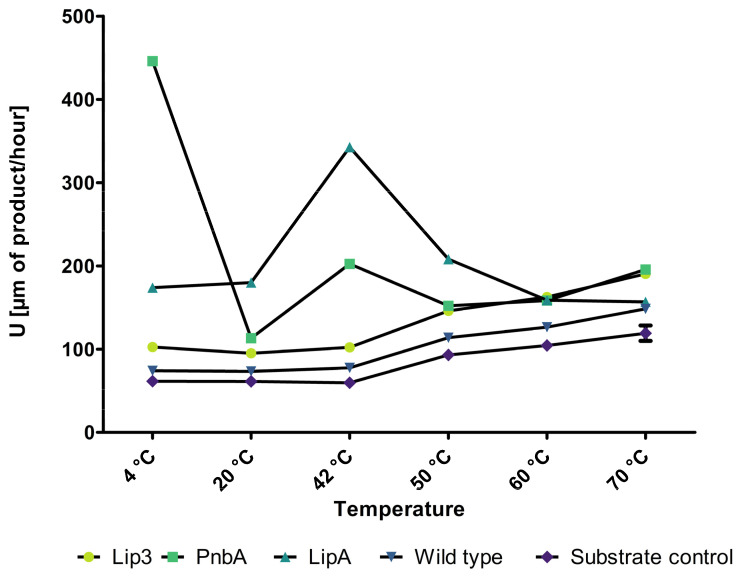
Activity Units of all three displayed enzymes, the wild-type spores, and substrate control plotted against the used temperatures. The assay used here was performed for 1 h at different temperatures and the C8 substrate. The *y*-axis shows the Units defined as the conversion of 1 µmol substrate into pNPB in one hour. The used spores were set to an OD_600_ of 1 and varied with the different temperatures (n = 3).

After validating the spore display of GFP, the next step was to express industrially relevant enzymes on the surface of *P. polymyxa* spores. The hydrolytic activity at different temperatures of purified spores expressing either lipase or esterase is shown in Figure 3. Figure 3 shows the different temperatures plotted against the absorbance levels. The first experiment was based on the lipase assay described by Hui et al., 2022 [28], conducted with an OD_600_ of 1 of spores, and showed hydrolysis activity for all constructs at 42 °C.

The absorbance at 410 nm is directly correlated to the activity of the spore display based on the standard curve of *p*-nitrophenol (Supplementary Figure S2). We defined the enzyme activity unit, U, as the conversion of 1 µm substrate into *p*-nitrophenol in one hour at 42 °C. The activities for all three displayed proteins varied concerning the respective substrate. For the long-chained fatty acids 4-Nitrophenyl-palmitate (C16) and 4-Nitrophenyl-dodecanoic (C12), the activities were at the level of the background and the substrate control (Supplementary Figures S3–S5). For the short-chained fatty acid 4-Nitrophenyl-octanoate (C8), the hydrolysis activities were significantly higher compared to the background, the substrate control, and longer-chain fatty acids. These results are comparable to those shown by Hui et al., 2022 [28]. Here, the same lipase (LipA) via *B. subtilis* spore display showed lower substrate affinities for smaller substrates in contrast to what was shown for the free enzyme [56,57]. After these first promising results at 42 °C, we tested our system with varying temperatures in a range from 4 °C to 70 °C.

The activity of the displayed esterase PnbA reached its optimum at 4 °C. The activity was 6-fold higher compared to the wild-type spores. The activity of the PnbA reached 446 U [µm of product/hour]. The LipA from *B. subtilis* showed the highest activity with 342 U [µm of product/hour] at 42 °C, 4.4-fold higher activity compared to the wild-type spores. The displayed Lip3 showed higher hydrolysis activity with rising temperature. It reached 190 U [µm of product/hour], which is 2.4-fold higher activity compared to the wild-type spores at 70 °C. Figure 3 shows the activities of all three displayed enzymes with the C8 substrate compared to the wild-type spores and the substrate control. The activity for the wild-type spores not displaying any enzyme can be explained by the autohydrolysis of the substrate with increasing temperatures, as shown in Figure 3. With the exception of Lip3, the assayed enzymes’ activity decreased from 42 °C to 50 °C (see Supplementary Figures S4 and S5). In order to evaluate higher temperature ranges for Lip3, the assay temperature was increased to 70 °C. Only the C8 substrate was used for the high-temperature assay because it showed the highest absorbance levels with all three enzymes on the spore display (Figure 3). The Lip3 doubled its activity from 40 °C to 70 °C. To evaluate the substrate consumption, a 16 h experiment was performed with Lip3, the wild-type spores, and the substrate as control at 42 °C (Supplementary Figure S7). The figure shows a three-times-higher absorbance level compared to the absorbance level at 70 °C, indicating slow substrate consumption and low activity of the displayed Lip3. Compared to the absorbance level at 42 °C, the 16 h experiment even reached a 6-fold higher absorbance level.

The activity of the LipA dropped from 42 °C to 60 °C to half of the maximum activity but kept this activity also at 70 °C and reached absorbance levels like the wild-type spores.

### 3.4. Recyclability and Reusability of the Spore Display

As the ease of spore purification is a major advantage of spore display, we wanted to assess the potential recyclability of our system, as demonstrated also in the *B. subtilis* spore display with simple centrifugation and washing steps (adapted from [28]). Exemplary recycling of CotE-LipA, CotE-Lip3, and CotE-PnbA spores was shown over 10 runs with the substrate 4-Nitrophenyl-octanoate. The experiment was performed as described for the lipase assay. The spore display for the Lip3 maintained its low activity as an immobilization particle after 10 cycles (Supplementary Figure S6). The activity of the immobilized enzymes, PnbA and LipA, dropped to a low activity. The CotE-LipA construct lost 45% hydrolytic activity after the first run and an additional 26% from runs 2 to 3. The CotE-PnbA lost 18% after the first and an additional 19% from runs 2 to 3. After the third run, the activities of all three enzymes stayed the same for four runs, dropping then nearly to the background activity from the wild-type spores and the substrate control (Supplementary Figure S6).

### 3.5. Hydrolytic Side Activity of the Lipoyl Synthase a Displayed on P. polymyxa Spores

**Figure 4:**
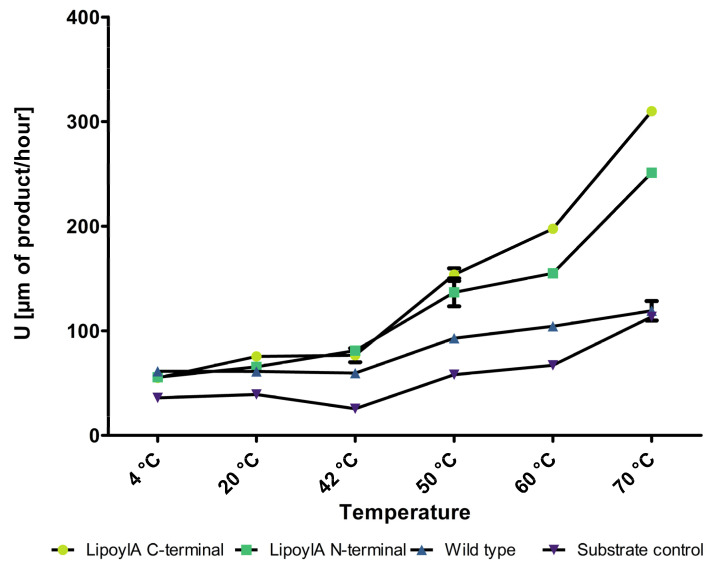
Activity Units of the lipoyl synthase displayed C- and N-terminal enzymes and the wild-type spores with different temperatures and the C8 substrate. The *y*-axis shows the Units defined as the conversion of 1 µmol substrate into pNPB in one hour. The used spores were set to an OD_600_ of 1 and varied with the different temperatures (n = 3).

The lipoyl synthase A, which belongs, in contrast to the lipases and esterases, to the class of synthases/transferases, is abbreviated as LipA in the well-structured *Subti*Wiki [45], which is also the abbreviation used for lipase A from *B. subtilis* in other major databases such as UniProt, where both lipase A and lipoyl synthase A are abbreviated as *lipA*. This inconsistent nomenclature made us consider and assay this synthase as a lipase until the mistake was recognized. Interestingly this enzyme showed hydrolytic activity for p-nitrophenyl caprylate (pNPC, C8), a substrate commonly used for lipase assays, revealing a hydrolytic activity of this enzyme. Furthermore, the lipoyl synthase showed increased activity in this assay with increasing temperature. This activity is likely not able to be accentuated by autohydrolysis or the spores themselves, as can be seen in Figure 4. The lipoyl synthase reached a maximum absorbance level at 410 nm of 1.04 which is 4.99-fold higher compared to the control. The correlation to the standard curve of *p*-nitrophenol gives 309 U [µm of product/hour].

**Figure 5:**
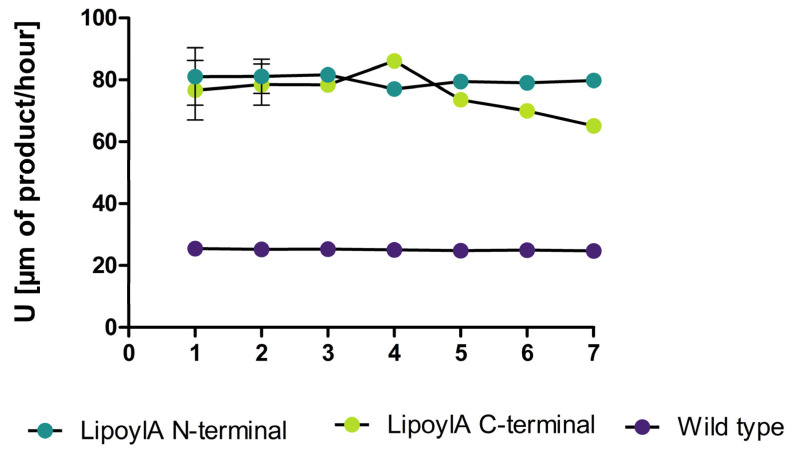
Recycling runs of the lipoyl synthase over seven runs. The recycling runs were performed according to the described method in the Materials and Methods section for 1 h at 42 °C (n = 3).

Additionally, we tested the recycling abilities of the lipoyl synthase and observed no significant loss in hydrolysis side activity over seven runs (Figure 5).

## 4. Discussion

We have successfully developed a spore surface display system for *P. polymyxa* DSM 365 utilizing an analog version of the spore surface display technology employed in *B. subtilis* [28]. Our findings demonstrate the system’s functionality through the fluorescence signal of CotE-GFP spores, the catalytic activity of two different lipases, and an esterase when fused to the anchoring protein. In a recently published manuscript about spore surface display in *B. subtilis,* the researchers observed a 50% activity loss over 50 °C with the LipA displayed [28]. Additionally, we saw, as described in the Results part, a significant activity for our spore display system. In comparison to this, a publication from Kim in 2017 saw no activity in *B. subtilis* with the same system [27]. With rising temperatures, we saw a loss in activity for the lipases and esterases but an increase in the measured activity of the lipoyl synthase. The lipoyl synthase has so far not been described as an iron–sulfur cluster enzyme required for the synthesis of lipoic acid, during which two sulfurs are transferred to an octanoic acid [57]. As both the substrates for the synthesis and the hydrolysis contain a C8 fatty acid, making a binding in the enzyme pocket likely, the observed hydrolytic could represent an example of substrate promiscuity [58], yet further experiments are required to confirm this. Previous studies using spore display of lipases and esterases demonstrated a substrate shift to preferably small- to medium-chain fatty acids, which the authors attributed to the local spore environment distorting substrate access [59]. We observed the same feature with lipase A displayed on *P. polymyxa* spores. In addition, we observed for the first time a high hydrolytic activity (446 U [µm of product/hour]) at 4 °C for the esterase PnbA from *P. polymyxa*. Cold-active esterases are described in different bacterial species but not from *P. polymyxa* [60,61,62,63]. *P polymyxa* is known to have a cold-active pullulanase [62]. Future work should use the free enzymes to determine which property changes arise from the immobilized forms. Concerning the recyclability properties of all four spore displays, the LipA showed a drop in activity after the first run to its half. Meanwhile, Lip3 activity remained in the same low range during recycling. The PnbA lost 18% of its activity after the first and an additional 19% after the second. In contrast to the two native and one heterologously expressed enzymes, the recyclability of the lipoyl synthase from *P. polymyxa* showed no significant loss in activity over seven runs (Figure 5). Our study serves as a foundation for expanding the application of spore surface display to *P. polymyxa.* Investigations of different linkers and promoters could further enhance the activity of displayed proteins. A previous study achieved an increase in the protein load on the spore surface and the activity of this system by knocking out extracellular proteases in *B. subtilis*, which would also be beneficial in *P. polymyxa* [28].

Additionally, in this study, the spore surface display was used for the first time with a TEV cleavage site between the linker and the protein. This enabled us to show that the displayed enzymes are not just absorbed by the spores but are a real fusion protein incorporated into the spore crust. This approach could enable the use of surface expression of diverse proteins on spores as a simple protein purification method. However, the system’s current cleavage efficiency is not optimal and requires further enhancements to achieve maximum efficacy. Future studies could allow the simultaneous expression of multiple enzyme types, facilitating the engineering of enzymatic cascades on a single spore surface and creating versatile biocatalysts capable of multiple chemical transformations like those already described for *B. subtilis* [24,61]. These developments are expected to contribute significantly to the field of sustainable biocatalysis.

## 5. Conclusions

In this study, we have demonstrated the suitability of the *P. polymyxa* spores for immobilization of enzymes. Our spore display approach offers several advantages, including cost, self-immobilization, and the ability to easily recover the immobilized enzymes for multiple cycles of use. Immobilization also improves protein handling and allows rapid separation of immobilized enzymes from the reaction product. In addition, spore display offers economic advantages, longer shelf life, and resistance to solvents compared to free enzymes [64,65]. This spore display approach provides significant advantages for protein immobilization and reuse. It could be used for testing different protein-displaying spores in a standardized manner. Furthermore, *P. polymyxa* wild-type spores are already applied in agriculture, and the present system for heterologous spore display could advance this application through spores displaying relevant enzymes such as phytases to increase phosphorus availability [66]. The free enzymes need to be compared to the spore display for further characterization of the lipases and the esterase of *P. polymyxa*. Additionally, the lipoyl synthase should be assayed for synthase activity while immobilized, to examine the effects of spore display on this enzyme. Overall, our results highlight the potential of *P. polymyxa* spore display as a versatile tool for protein immobilization and offer a promising avenue for further research and application development in this field.

## Data Availability

The datasets used and/or analyzed during the current study are available from the corresponding author on reasonable request.

## Supplementary Materials

The following supporting information can be downloaded at: https://www.mdpi.com/article/10.3390/microorganisms12071438/s1, Figure S1: Calibration curve of the correlation between spores and OD600; Figure S2: Standard curve for the absorbance of p-nitrophenol at 410 nm; Figure S3: First lipase assay performed showing activity compared to the controls after 1 h and 42°C; Figure S4: Lipase assay performed with C12 substrate and temperatures from 4 °C to 50 °C with all three displayed enzymes, the wild type spores, and the substrate as control; Figure S5: Lipase assay performed with C16 substrate and temperatures from 4 °C to 50 °C with all three displayed enzymes, the wild type spores, and the substrate as control; Figure S6: Spore recycling of all three spore displayed enzymes and the substrate control over 10 runs exemplary with the substrate 4-Nitrophenyl-octanoate; Figure S7: Lipase assay performed with the Lip3 enzyme and C8 substrate for 16 h at 42 °C; Figure S8: Plasmidmap of the spore display including the nativ promotor side (orange), the anchoring protein (yellow), the flex-linker and TEV recognition site (red) and the fusion protein (here in green sfGFP); Figure S9: Exemplary schematic design of the used spore display from expression inside the cells to the lysis of the mature spores including the displayed proteins on there crust; Table S1: List of strains and organisms used in this study; Table S2: List of plasmids used in this study; Table S3: List of primers used in this study; Table S4: Composition of the vitamin solution and the Trace Elements; Table S5: 10× Phosphate-buffered saline (10× PBS); Table S6: Recycling runs of all three spore displayed enzymes with their respective loss in activity after each run.
