# Supplementary material for: Implementation of Spore Display in Paenibacillus polymyxa with Different Hydrolytic Enzymes
Source: Microorganisms. 2024 Jul 16;12(7):1438. doi: 10.3390/microorganisms12071438 (PMC11278568; doi:10.3390/microorganisms12071438)
Supplement: Supplementary file 1 [file microorganisms-12-01438-s001.zip › microorganisms-3097717-supplementary.pdf]

Supplementary material for the journal **Microorganisms**

Implementation of spore display in *Paenibacillus polymyxa* with different hydrolytic enzymes

Maximilian Zander[1], Jochen Schmid[1] and Johannes Kabisch[2]*

[1]Institute for Molecular Microbiology and Biotechnology, University of Münster, Correnstrasse 3, 48149 Münster, Germany

[2] Department of Biotechnology and Food Science, NTNU Trondheim, Kjemi 3, Gløshaugen, Trondheim, Norway

*Corresponding author

Authors:

Maximilian Zander: max-zander@live.de

Jochen Schmid: jochen.schmid@uni-muenster.de

Johannes Kabisch: johannes.kabisch@ntnu.no

Prof. Dr. Johannes Kabisch

Kjemi 3, Gløshaugen, Sem Sælands vei 6

7034 Trondheim

Norway

Phone: +4773412932

Email: johannes.kabisch@ntnu.no

Strains list and organisms

Table S1 List of strains and organisms used in this study

Strains	Genotype and description	Source or reference
E. coli TOP10	F-mcrAΔ(*mrr-hsdRMS-mcrBC*) Φ80*LacZ*ΔM15 Δ*Lac*X74 *rec*A1 *ara*D139 Δ(*araleu*) 7697*gal*U *gal*K *rps*L (StrR) *end*A1 *nup*G	Invitrogen
E. coli Turbo	F' *proA*$^{+}$*B*$^{+}$ *lacI*^{q} Δ*lacZM15 / fhuA2* Δ(*lac-proAB) glnV galK16 galE15 R*(*zgb-210::Tn10*)TetS *end*A1 *thi*-1 Δ(*hsdS-mcrB*)5	NEB
E. coli S17-1	Conjugation strain; *recA* pro *hsdR RP42Tc::Mu-Km::Tn7* integrated into the chromosome	ATCC 47055
P. polymyxa DSM 365	Wild type	DSMZ
S50002	*P. polymyxa* strain carrying the cotE-LipoylA spore display (C-terminal)	This Work
S50003	*P. polymyxa* strain carrying the cotE-LipoylA spore display (N-terminal)	This Work
S50004	*P. polymyxa* strain carrying the cotE-GFP spore display	This Work

S50005	*P. polymyxa* strain carrying the cotE-TEV-GFP spore display	This Work
S50006	*P. polymyxa* strain carrying the cotE-LipA spore display	This Work
S50007	*P. polymyxa* strain carrying the cotE-Lip3 spore display	This Work
S50008	*P. polymyxa* strain carrying the cotE-PnbA spore display	This Work

Table S2 List of plasmids used in this study.

**Plasmid**	**Description**	**Source or reference**
pCasPP	*P. polymyxa* CRISPR-Cas9 genome editing plasmid	[59]
pHEiP	Empty shuttle vector based on the pCasPP for the expression of the spore display	This study
pHEiP-CotE_GFP	High-copy expression plasmid for the spore display with *pcotE* and the flexible linker in between	This study
pHEiP-CotE_TEV_GFP	High-copy expression plasmid for the spore display with *pcotE* and the flexible linker and the TEV-cleavage site in between	This study

pHEiP-CotE_LipA	High-copy expression plasmid for the spore display with *pcotE* and the flexible linker in between	This study
pHEiP-CotE_Lip3	High-copy expression plasmid for the spore display with *pcotE* and the flexible linker in between	This study
pHEiP-CotE_PnbA	High-copy expression plasmid for the spore display with *pcotE* and the flexible linker in between	This study
pHEiP-CotE_LipoylA (C-terminal)	High-copy expression plasmid for the spore display with *pcotE* and the flexible linker in between	This study
pHEiP-CotE_LipoylA (N-terminal)	High-copy expression plasmid for the spore display with *pcotE* and the flexible linker in between	This study

Table S3 List of primers used in this study.

**Primer**	**Sequence (5´-3´)**

BB_fwd	CATCATCACCACCACCATTAATCTAGAGTCGACGTCCCC
BB_rev	AAAGGATTGAAGGATGGGAG
Promotor_fwd	AAAGGATTGAAGGATGGGAGCGCTGGTTACCTATACCC
Promotor_rev	TCTTGAACCTCCTCCATACAATC
CotE_fwd	GATTGTATGGAGGAGGTTCAAGAATGGCATTAAGTCATAAAAATCGTAG
CotE_Linker_rev	ACCTCCACCACCTCCACCACTTCCACCACCTCCACCGAGCTCGTCGTCAATGAGG
GFP_Linker_fwd	GGTGGAGGTGGTGGAAGTGGTGGAGGTGGTGGAGGTATGCGTAAAGGCGAAGAGC
GFP_HIS_rev	TAGATTAATGGTGGTGGTGATGATGTTTGTACAGTTCATCCATACCATGC
CotE_Linker_TEV_rev	AGATTCTCACCACCACCTCCACCACTTCCACCACCTCCACCGAGCTCGTCGTCAATGAG
GFP_Linker_TEV_fwd	GAAGTGGTGGAGGTGGTGGTGAGAATCTTTATTTTCAGGGCATGCGTAAAGGCGAAGAG

LipA_Linker_fwd	GGTGGAGGTGGTGGAAGTGGTGGAGGTGGTGGAGGTATGAAATTTGTAAAAAGAAGGAT
LipA_HIS_rev	TTAATGGTGGTGGTGATGATGATTCGTATTCTGGCCCC
Lip3_Linker_fwd	GGTGGAGGTGGTGGAAGTGGTGGAGGTGGTGGAGGTATGCGGGAATACACCTTCA
Lip3_HIS_rev	ACTCTAGATTAATGGTGGTGGTGATGATGCAAGGCTGATGACTTGGGC
PnbA_Linker_fwd	GGTGGAGGTGGTGGAAGTGGTGGAGGTGGTGGAGGTATGGAGAGTATTACAGTACATAC
PnbA_HIS_rev	TAGATTAATGGTGGTGGTGATGATGTAGGGTCATCTGACCGGT
CotE_Linker_rev_C-terminal	ACCTCCACCACCTCCACCACTTCCACCACCTCCACCGAGCTCGTCGTCAATGAGG
CotE_fwd_ C-terminal	TGATTGTATGGAGGAGGTTCAAGAATGGCATTAAGTCATAAAAATCGTAG
LipoylA_Linker_fwd	TGGTGGAAGTGGTGGAGGTGGTGGAGGTTTGTCTAGAAAAGAGAAGGAAG
LipoylA_HIS_rev	TCGACTCTAGATTAATGGTGGTGGTGATGATGAGCCTGCGAAAGGGCGCT

CotE_Linker_fwd_N-terminal	GGTGGAGGTGGTGGAAGTGGTGGAGGTGGTGGAGGTATGGCATTAAGTCATAAAAATC
CotE_HIS_rev_ N-terminal	TCGACTCTAGATTAATGGTGGTGGTGATGATGGAGCTCGTCGTCAATGAG
LipoylA_Linker_rev-N-terminal	CCACCACCTCCACCACTTCCACCACCTCCACCAGCCTGCGAAAGGGCGCT
LipoylA_fwd_N-terminal	TGATTGATTGTATGGAGGAGGTTCAAGATTGTCTAGAAAAGAGAAGGAAG

Table S4 Composition of the vitamin solution and the Trace Elements.

Trace Elements (store at 4 °C)

Substance	Amount	Concentration
$MnCl_2.4H_2O$	1.80	g/L
$FeSO_4.7H_2O$	2.50	g/L
Boric acid	258	mg/L
$CuSO_4.\ 7H_2O$	31.0	mg/L

$ZnCl_2$	21.0	mg/L
$CoCl_2.6H_2O$	75.0	mg/L
$MgMoO_4$	23.0	mg/L
Sodium tartrate.$2H_2O$	2.10	g/L

Vitamin solution RPMI 1640 100X (store at -20 °C)

Substance	Weight	Concentration
D-Biotin	0.02	g/L
Choline chloride	0.3	g/L
Folic acid	0.1	g/L
myo-Inositol	3.5	g/L
Niacinamide	0.1	g/L
p-Amino benzoic acid	0.1	g/L
D-Patothenic acidx 0.5 Ca	0.025	g/L
Pyridoxal.HCl	0.1	g/L
Riboflavin	0.02	g/L

Thiaminel.HCl	0.1	g/L
Vitamin B12	0.0005	g/L
KCl	0.2	g/L
KH_2PO_4 (anhydrous)	0.2	g/L
NaCl	8.0	g/L
Na_2HPO_4 (anhydrous)	1.15	g/L

Table S5 10x Phosphate-buffered saline (10x PBS)

Substance	Amount per 1 L
NaCl	80 g
KCl	2 g
Na_2HPO_4	18 g
KH_2PO_4	2.4 g
dH_2O	to 1 L

Table S6 Recycling runs of all three spore displayed enzymes with their respective loss in activity after each run.

Recycling Runs	Lip3 [A410 nm]	Change in %	PnbA [A410 nm]	Change in %	LipA [A410 nm]	Change in %
1	3,4433E-01	/	6,8187E-01	/	1,1549E+00	/
2	3,9179E-01	-14%	5,6180E-01	18%	6,3360E-01	45%
3	3,8470E-01	2%	4,5480E-01	19%	4,7157E-01	26%
4	3,5587E-01	7%	4,4443E-01	2%	4,6197E-01	2%
5	3,4897E-01	2%	4,2273E-01	5%	4,2637E-01	8%
6	3,4707E-01	1%	4,0180E-01	5%	4,1923E-01	2%
7	3,1297E-01	10%	3,6830E-01	8%	3,9290E-01	6%
8	2,8870E-01	8%	3,4555E-01	6%	3,3160E-01	16%
9	2,7643E-01	4%	2,9600E-01	14%	2,6603E-01	20%
10	2,6287E-01	5%	2,5137E-01	15%	2,4897E-01	6%

Supplementary figures

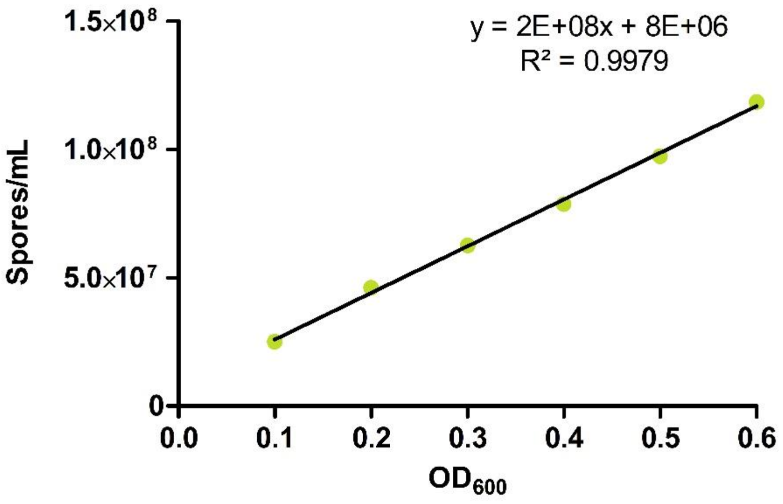

Supplement Figure S1: Calibration curve of the correlation between spores and OD_{600}. Different amounts of spores in water were set to different OD-values and measured in the cytometer. A linear fitting was performed for the regression curve giving an R^2 value of 0.9979.

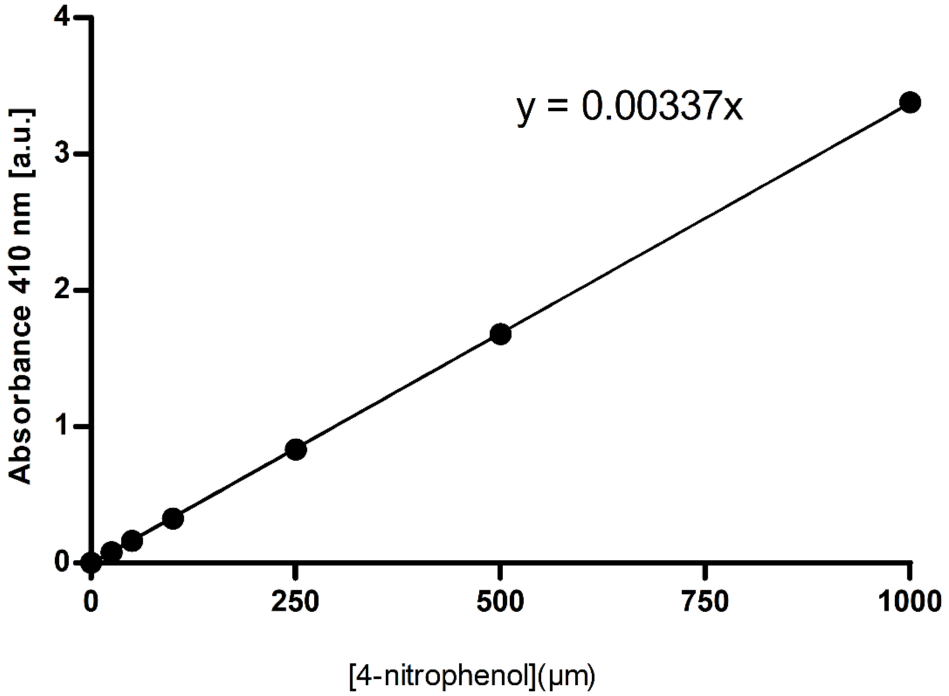

Supplement Figure S2: Standard curve for the absorbance of p-nitrophenol at 410 nm.

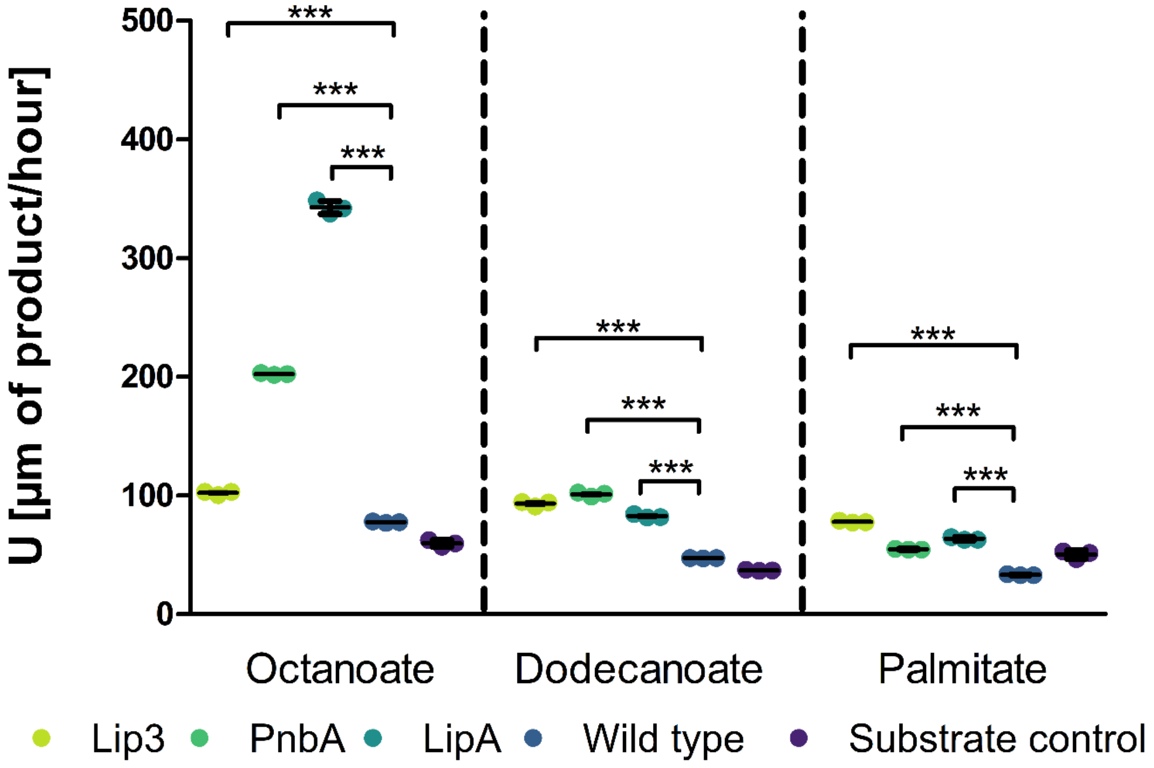

Supplement Figure S3: First lipase assay performed showing activity compared to the controls after 1 h and 42°C. P value <0.05 was considered in determining the significant differences between the hydrolysis activities (* $p < 0.05$, ** $p < 0.01$, *** $p < 0.001$).). The y-axis shows the U defined as the conversion of 1 µmol substrate into pNPB in one hour. The used spores were set to an OD_{600} of 1(n= 3)

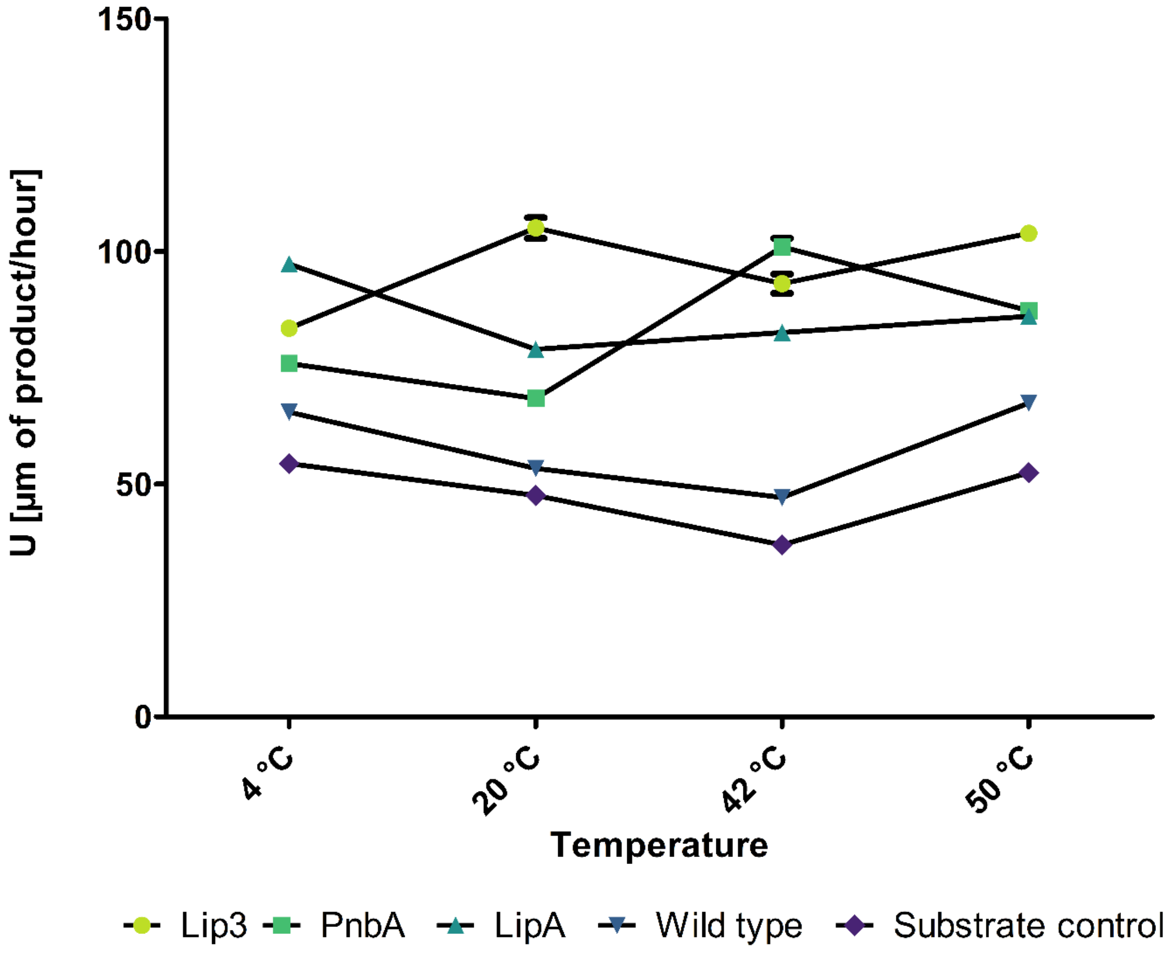

Supplement Figure S4: Lipase assay performed with C12 substrate and temperatures from 4 °C to 50 °C with all three displayed enzymes, the wild type spores, and the substrate as control. The y-axis shows the U defined as the conversion of 1 µmol substrate into pNPB in one hour. The used spores were set to an OD_{600} of 1(n= 3)

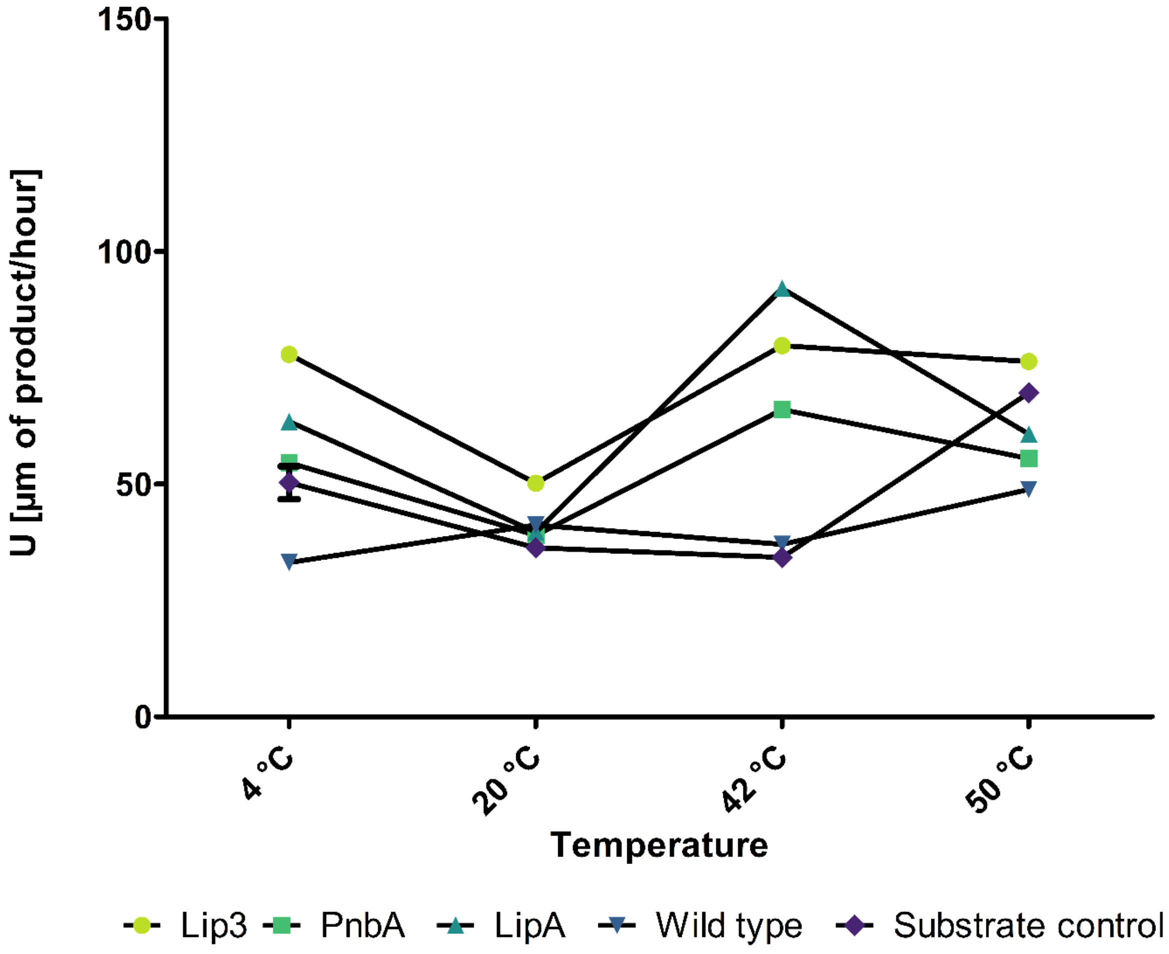

Supplement Figure S5: Lipase assay performed with C16 substrate and temperatures from 4 °C to 50 °C with all three displayed enzymes, the wild type spores, and the substrate as control. The y-axis shows the U defined as the conversion of 1 µmol substrate into pNPB in one hour. The used spores were set to an OD_{600} of 1(n= 3)

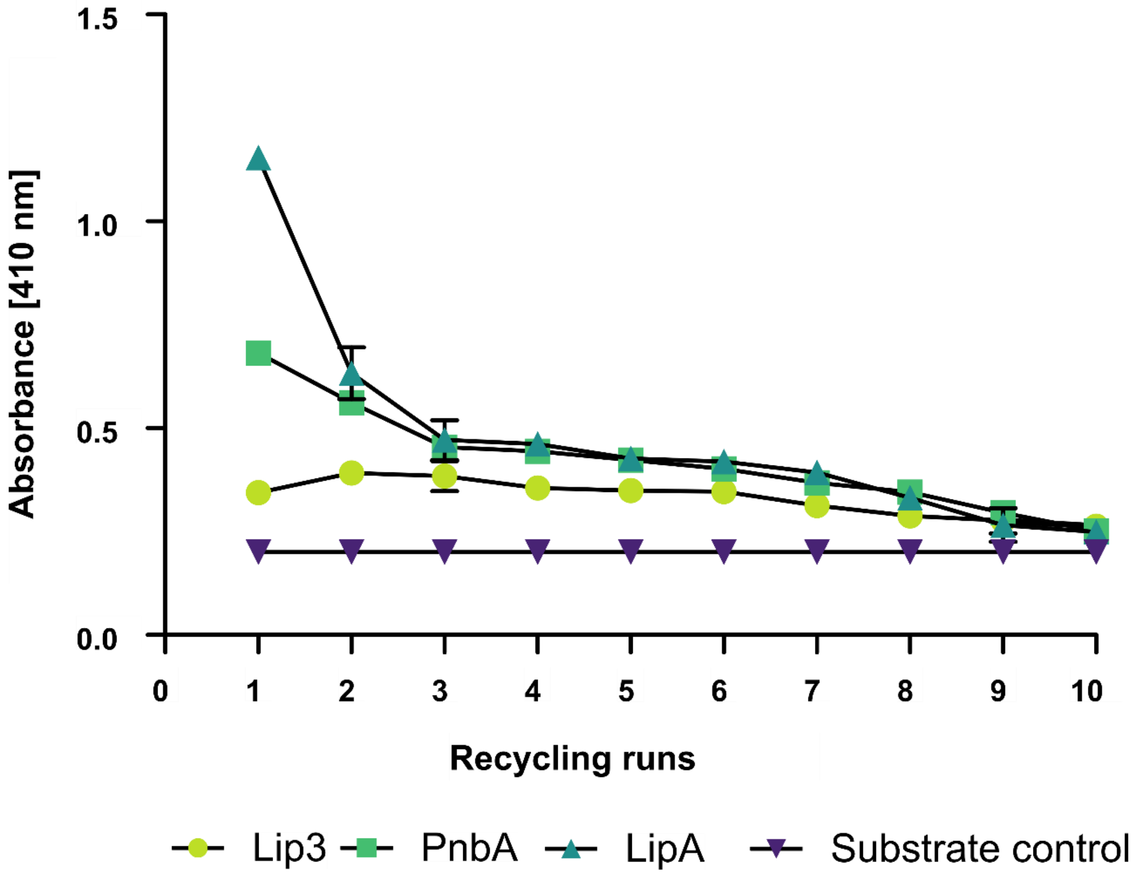

Supplement Figure S6: Spore recycling of all three spore displayed enzymes and the substrate control over 10 runs exemplary with the substrate 4-Nitrophenyl-octanoate. The experiments were performed at 42 °C, 800 rpm, and for 1 h as described in the Material and Methods section. The used spores were set to an OD_{600} of 1. (n= 3)

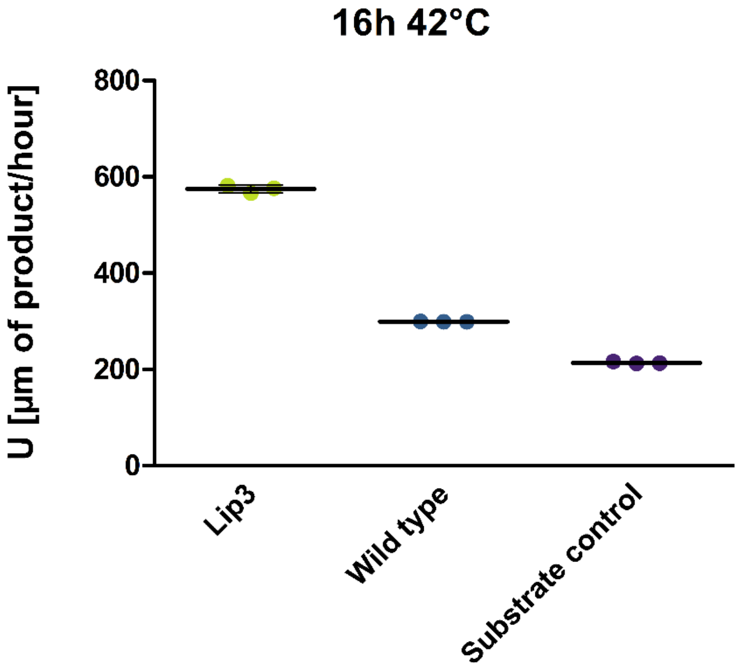

Supplement Figure S7: Lipase assay performed with the Lip3 enzyme and C8 substrate for 16h at 42 °C. As controls the wild type spores and the substrate control is shown. The y-axis shows the U defined as the conversion of 1 µmol substrate into pNPB in one hour. The used spores were set to an OD_{600} of 1.

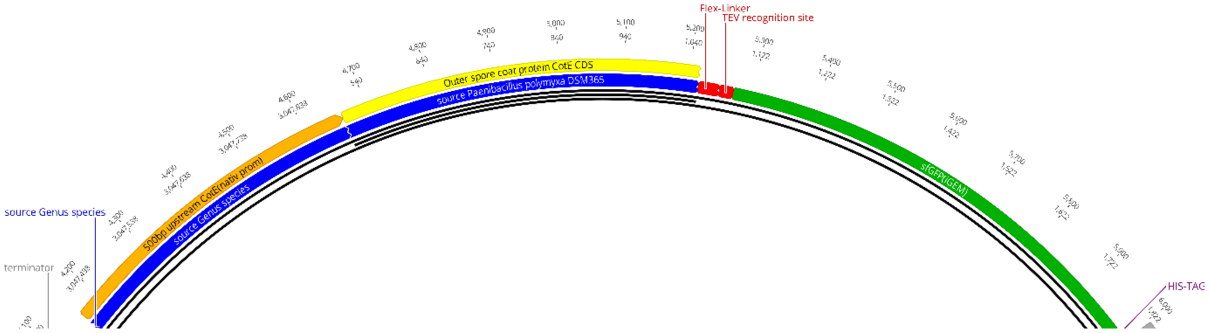

Supplement Figure S8: Plasmidmap of the spore display including the nativ promotor side (orange), the anchoring protein (yellow), the flex-linker and TEV recognition site (red) and the fusion protein (here in green sfGFP).

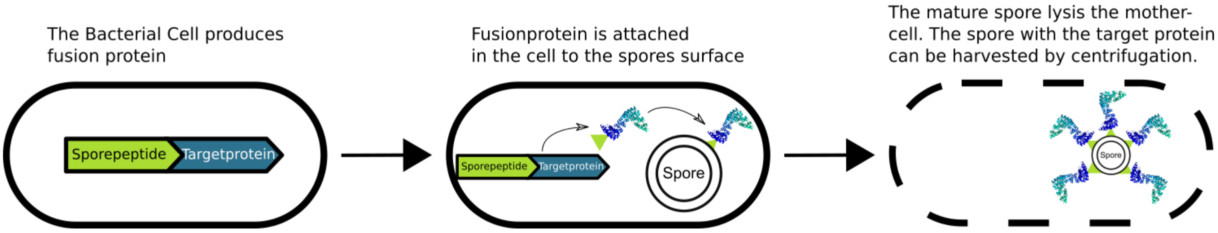

Supplement Figure S9: Exemplary schematic design of the used spore display from expression inside the cells to the lysis of the mature spores including the displayed proteins on there crust.

Genbank sequenze of the pHeip plasmid carrying the cotE-GFP spore display.
LOCUS pHEiPspacZ_sporedisplayGFP_c-terminal 6279 bp DNA circular UNA 19-AUG-2022
DEFINITION .
ACCESSION urn.local...x4-ee4vfx3
VERSION urn.local...x4-ee4vfx3
KEYWORDS .

```
SOURCE
  ORGANISM  .
FEATURES             Location/Qualifiers
     misc_feature    complement(1..22)
                     /note="Promotor"
                     /standard_name="rpsLp(XC)"
     misc_feature    complement(23..510)
                     /standard_name="oriT"
     primer_bind     complement(117..136)
                     /standard_name="seq_Harms_R"
     oriT            244..353
                     /note="incP origin of transfer"
                     /standard_name="oriT"
     primer_bind     378..402
                     /standard_name="seq_PSG5_F"
     primer_bind     complement(523..553)
                     /standard_name="GG_oriT_R"
     primer_bind     complement(535..549)
                     /standard_name="oriT_XbaI.FOR"
     primer_bind     550..578
                     /standard_name="GG_oriT_F"
     rep_origin      556..637
     rep_origin      complement(592..1180)
     primer_bind     complement(679..696)
                     /standard_name="seq_sg_pCasMC.REV"
     primer_bind     complement(767..786)
                     /standard_name="XbaI_ori_rev"
     primer_bind     1101..1125
                     /standard_name="oriMCSseq2"
     primer_bind     1207..1226
                     /standard_name="kan_fwd"
     primer_bind     complement(1231..1254)
                     /standard_name="GG_ori.FOR"
     primer_bind     complement(1235..1254)
                     /standard_name="pUC_oriMCS_BbsI_fwd"
     primer_bind     complement(1341..1367)
                     /standard_name="GG_Cas9Com2.REV"
     primer_bind     1372..1396
                     /standard_name="GG_neo.FOR"
     primer_bind     1377..1398
                     /standard_name="neo_rev"
     misc_feature    1378..1383
                     /standard_name="-35"
     misc_feature    1400..1408
                     /standard_name="-10"
     misc_feature    1447..2217
                     /note="neo"
                     /standard_name="neo"
     primer_bind     complement(1519..1552)
                     /standard_name="seq_neo.REV"
     primer_bind     complement(1571..1595)
                     /standard_name="oriMCSseq1"
     primer_bind     complement(2292..2311)
                     /standard_name="pUB110_rGI2_ligCtrl_rev"
     primer_bind     complement(2442..2463)
                     /standard_name="neo_fwd"
```

primer_bind 2717..2736
/standard_name="seq_BsaI.FOR"
primer_bind complement(2867..2896)
/standard_name="GG_neo.REV"
primer_bind 2892..2924
/standard_name="GG_bsaI.FOR"
misc_feature 2896
/note="transcription terminator (beta)"
/standard_name="terminator"
misc_feature 3028..3033
/standard_name="-35"
misc_feature 3048..3056
/standard_name="-10"
misc_feature join(3086..3189,3190..4090)
/note="plasmid replication protein from Staph. aureus"
/standard_name="repU"
primer_bind complement(3163..3193)
/standard_name="GG_bsaI.REV"
primer_bind 3190..3220
/standard_name="GG_repU.FOR"
primer_bind 4031..4060
/standard_name="seq_repu.FOR"
misc_feature 4091..4116
/note="transcription terminator"
/standard_name="terminator"
misc_feature 4161..5987
/Original_Bases="AGAAGATTTATTTGAGGTAGCCCTTGCCTACCTAGCTTCCA

AGAAAGATATCCTAACAGCACAAGAGCGGAAAGATGTTTTGTTCTACATCCAGAACAA

CCTCTGCTAAAATTCCTGAAAAATTTTGCAAAAAGTTGTTGACTTTATCTACAAGGTG

TGGCATAATGTGTGGAATTGTGAGCGGATAACAATTAAAGGAGGAAGGATCCACGCAA

GTCTTCTCAGCCGCTACAGGGCGCGTCCCATTCGCCATTCAGGCTGCGCAACTGTTGG

GAAGGGCGATCGGTGCGGGCCTCTTCGCTATTACGCCAGCTGGCGAAAGGGGGATGTG

CTGCAAGGCGATTAAGTTGGGTAACGCCAGGGTTTTCCCAGTCACGACGTTGTAAAAC

GACGGCCAGTGAGCGCGCGTAATACGACTCACTATAGGGCGAATTGGGTACCGGGCCC

CCCCTCGAGGTCCTCCAGCTTTTGTTCCCTTTAGTGAGGGTTAATTGCGCGCTTGGCG

TAATCATGGTCATAGCTGTTTCCTGTGTGAAATTGTTATCCGCTCACAATTCCACACA

ACATACGAGCCGGAAGCATAAAGTGTAAAGCCTGGGGTGCCTAATGAGTGAGCTAACT

CACATTAATTGCGTTGCGCTCACTGCCCGCTTTCCACCGGTGAAGACATGTTT"

/label="AGAAGATTTATTTGAGGTAGCCCTTGCCTACCTAGCTTCCAAGAAAGATA

TCCTAACAGCACAAGAGCGGAAAGATGTTTTGTTCTACATCCAGAACAACCTCTGCTA

AAATTCCTGAAAAATTTTGCAAAAAGTTGTTGACTTTATCTACAAGGTGTGGCATAAT

GTGTGGAATTGTGAGCGGATAACAATTAAAGGAGGAAGGATCCACGCAAGTCTTCTCA

GCCGCTACAGGGCGCGTCCCATTCGCCATTCAGGCTGCGCAACTGTTGGGAAGGGCGA

TCGGTGCGGGCCTCTTCGCTATTACGCCAGCTGGCGAAAGGGGGATGTGCTGCAAGGC

GATTAAGTTGGGTAACGCCAGGGTTTTCCCAGTCACGACGTTGTAAAACGACGGCCAG

TGAGCGCGCGTAATACGACTCACTATAGGGCGAATTGGGTACCGGGCCCCCCCTCGAG

GTCCTCCAGCTTTTGTTCCCTTTAGTGAGGGTTAATTGCGCGCTTGGCGTAATCATGG

TCATAGCTGTTTCCTGTGTGAAATTGTTATCCGCTCACAATTCCACACAACATACGAG

CCGGAAGCATAAAGTGTAAAGCCTGGGGTGCCTAATGAGTGAGCTAACTCACATTAAT
TGCGTTGCGCTCACTGCCCGCTTTCCACCGGTGAAGACATGTTT"
/note="Geneious type: Editing History Replacement"
source 4161
/organism="Genus species"
/mol_type="genomic DNA"
/strain="strain"
unsure 4162..4661
/created_by="mza"
/modified_by="mza"
/label="500bp upstream CotE(nativ prom)"
source <4162..>4661
/organism="Genus species"
/mol_type="genomic DNA"
/strain="strain"
RBS 4650..4654
/created_by="mka"
/label
CDS 4662..>5216
/db_xref="SEED:fig|1406.214.peg.1727"
/product="Outer spore coat protein CotE"
/transl_table=11
/Original_Translation="MALSHKNRREIITKAICGKGRRFSTVTHTVTPPNN

PTSILGAWIINHQYEAVAAGDGIEVVGTYDINIWYSYDKNSQTDVAKETVSYVENVPL

SYLDPKHRASTVEVSAEATQEPSCVEASVSSGGGSVMIRVEREFAVELVAETKIVVEV
FPNGSSDDFDKDFDFGAEEGDYEELDPDLIDDEL"
source <4662..>5216
/mol_type="genomic DNA"
/db_xref="taxon:1406"
/genome_md5="/project=""GRMZ_1406"
/genome_id="1406.214"
/organism="Paenibacillus polymyxa DSM365"
primer_bind 4662..4687
/created_by="mza"
/Sequence="ATGGCATTAAGTCATAAAAATCGTAG"
/Tm_(binding_region)="56.5"
/Tm_(with_extension)="72.1"
/%GC_(binding_region)="30.8"
/%GC_(with_extension)="32.8"

```
                     /Extension="CATAAAAACGAAGGGGGATTTTAGGCTTTTACTTA"
                     /modified_by="mza"
                     /label="o50031"
     primer_bind     complement(5198..5216)
                     /created_by="mza"
                     /Sequence="GAGCTCGTCGTCAATGAGG"
                     /Tm_(binding_region)="58.0"
                     /Tm_(with_extension)="76.4"
                     /%GC_(binding_region)="57.9"
                     /%GC_(with_extension)="61.8"
                     /Hairpin_Tm_(with_extension)="44.3"
                     /Self_Dimer_Tm_(with_extension)="28.9"
                     /Extension="ACCTCCACCACCTCCACCACTTCCACCACCTCCACC"
                     /modified_by="mza"
                     /label="o50032"
     linker          5217..5252
                     /created_by="User"
                     /modified_by="Max Zander"
                     /label="Flex-Linker"
     gene            5253..>5966
                     /modified_by="User"
                     /label="sfGFP(iGEM)"
     primer_bind     complement(5947..5966)
                     /created_by="mza"
                     /Sequence="TTTGTACAGTTCATCCATAC"
                     /Tm_(binding_region)="50.5"
                     /Tm_(with_extension)="67.6"
                     /%GC_(binding_region)="35.0"
                     /%GC_(with_extension)="41.8"
                     /Hairpin_Tm_(with_extension)="45.2"
                     /Self_Dimer_Tm_(with_extension)="20.0"
                     /Extension="ACAAATCCAGATGGAGTATGGTGGTGGTGATGATG"
                     /modified_by="mza"
                     /label="o50033"
     motif           5967..5984
                     /created_by="mza"
                     /modified_by="Max Zander"
                     /label="HIS-TAG"
     misc_feature    5988..6270
                     /created_by="Max Zander"
                     /label="rapA"
     primer_bind     complement(6219..6274)
                     /standard_name="Fragment.REV"
     primer_bind     6245..12
                     /standard_name="Vector.FOR"
     misc_feature    6260..6272
                     /standard_name="PsfsE_mcs_rev"
     primer_bind     6260..24
                     /standard_name="PsgsE_fw"
     primer_bind     6268..13
                     /standard_name="pHEIP_MCS_fw"
     primer_bind     6276..20
                     /standard_name="oriT_XbaI.REV"
ORIGIN
        1 acctgacttc cgcctgcagg gccagctcgc ggacgtgctc atagtccacg acgcccgtga
       61 ttttgtagcc ctggccgacg gccagcaggt aggccgacag gctcatgccg gccgccgccg
```

121 cctttcctc aatcgctctt cgttcgtctg gaaggcagta caccttgata ggtgggctgc
181 ccttcctggt tggcttggtt tcatcagcca tccgcttgcc ctcatctgtt acgccggcgg
241 tagccggcca gcctcgcaga gcaggattcc cgttgagcac cgccaggtgc gaataaggga
301 cagtgaagaa ggaacacccg ctcgcgggtg ggcctacttc acctatcctg cccggctgac
361 gccgttggat acaccaagga aagtctacac gaaccctttg gcaaaatcct gtatatcgtg
421 cgaaaaagga tggatatacc gaaaaaatcg ctataatgac cccgaagcag ggttatgcag
481 cggaaaagat ccgtcgacct gcatactagc tgctcaaggt cgcccgcaac cggcgcatca
541 agcccgccga ctagtggcca ggaaccgtaa aaaggccgcg ttgctggcgt ttttccatag
601 gctccgcccc cctgacgagc atcacaaaaa tcgacgctca agtcagaggt ggcgaaaccc
661 gacaggacta taaagatacc aggcgtttcc ccctggaagc tccctcgtgc gctctcctgt
721 tccgaccctg ccgcttaccg gatacctgtc cgcctttctc ccttcgggaa gcgtggcgct
781 ttctcatagc tcacgctgta ggtatctcag ttcggtgtag gtcgttcgct ccaagctggg
841 ctgtgtgcac gaaccccccg ttcagcccga ccgctgcgcc ttatccggta actatcgtct
901 tgagtccaac ccggtaagac acgacttatc gccactggca gcagccactg gtaacaggat
961 tagcagagcg aggtatgtag gcggtgctac agagttcttg aagtggtggc ctaactacgg
1021 ctacactaga agaacagtat ttggtatctg cgctctgctg aagccagtta ccttcggaaa
1081 aagagttggt agctcttgat ccggcaaaca aaccaccgct ggtagcggtg gtttttttgt
1141 ttgcaagcag cagattacgc gcagaaaaaa aggatctcaa gaagatcctt tgatcttttc
1201 tacggggtct gacgctcagt ggaacgaaaa ctcacgttaa gggattttgg tcatgagatt
1261 atcaaaaagg atcttcacct agatcctttt ggttcatgtg cagctccatc agcaaaaggg
1321 gatgataagt ttatcaccac cgactatttg caacagtgcc gttaatggtt ataatagctg
1381 aataagaacg gtgctctcca aatattctta tttagaaaag caaatctaaa attatctgaa
1441 aagggaatga gaatagtgaa tggaccaata ataatgacta gagaagaaag aatgaagatt
1501 gttcatgaaa ttaaggaacg aatattggat aaatatgggg atgatgttaa ggctattggt
1561 gtttatggct ctcttggtcg tcagactgat gggccctatt cggatattga gatgatgtgt
1621 gtcatgtcaa cagaggaagc agagttcagc catgaatgga caaccggtga gtggaaggtg
1681 gaagtgaatt ttgatagcga agagagattcta ctagattatg catctcaggt ggaatcagat
1741 tggccgctta cacatggtca atttttctct attttgccga tttatgattc aggtggatac
1801 ttagagaaag tgtatcaaac tgctaaatcg gtagaagccc aaacgttcca cgatgcgatt
1861 tgtgccctta tcgtagaaga gctgtttgaa tatgcaggca aatggcgtaa tattcgtgtg
1921 caaggaccga caacatttct accatccttg actgtacagg tagcaatggc aggtgccatg
1981 ttgattggtc tgcatcatcg catctgttat acgacgagcg cttcggtctt aactgaagca
2041 gttaagcaat cagatcttcc ttcaggttat gaccatctgt gccagttcgt aatgtctggt
2101 caactttccg actctgagaa acttctggaa tcgctagaga atttctggaa tgggattcag
2161 gagtggacag aacgacacgg atatatagtg gatgtgtcaa aacgcatacc attttgaacg
2221 atgacctcta ataattgtta atcatgttgg ttacgtattt attaacttct cctagtatta
2281 gtaattatca tggctgtcat ggcgcattaa cggaataaag ggtgtgctta aatcgggcca
2341 ttttgcgtaa taagaaaaag gattaattat gagcgaattg aattaataat aaggtaatag
2401 atttacatta gaaaatgaaa ggggatttta tgcgtgagaa tgttacagtc tatcccggca
2461 ttgccagtcg gggatattaa aaagagtata ggtttttatt gcgataaact aggtttcact
2521 ttggttcacc atgaagatgg attcgcagtt ctaatgtgta atgaggttcg gattcatcta
2581 tgggaggcaa gtgatgaagc tggcgctctc gtagtaatga ttcaccggtt tgtacaggtg
2641 cggagtcgtt tattgctggt actgctagtt gccgcattga agtagaggga attgatgaat
2701 tatatcaaca tattaagcct ttgggcattt tgcaccccaa tacatcatta aaagatcagt
2761 ggtgggatga acgagacttt gcagtaattg atcccgacaa caatttgatt agcttttttc
2821 aacaaataaa aagctaaaat ctattattaa tctgttcagc aatcgggcgc gattgctgaa
2881 taaaagatac gaaggtgatg gttttgaact tgttctttct tatcttgata catatagaaa
2941 taacgtcatt tttattttag ttgctgaaag gtgcgttgaa gtgttggtat gtatgtgttt
3001 taaagtattg aaaaccctta aaattggttg cacagaaaaa ccccatctgt taaagttata
3061 agtgactaaa caaataacta aatagatggg ggtttctttt aatattatgt gtcctaatag
3121 tagcatttat tcagatgaaa aatcaagggt tttagtggac aagacaaaaa gtggaaaagt
3181 gaggccatgg agagaaaaga aaatcgctaa tgttgattac tttgaacttc tgcatattct
3241 tgaatttaaa aaggctgaaa gagtaaaaga ttgtgctgaa atattagagt ataaacaaaa
3301 tcgtgaaaca ggcgaaagaa agttgtatcg agtgtggttt tgtaaatcca ggctttgtcc
3361 aatgtgcaac tggaggagag caatgaaaca tggcattcag tcacaaaagg ttgttgctga
3421 agttattaaa caaaagccaa cagttcgttg gttgtttctc acattaacag ttaaaaatgt

3481 ttatgatggc gaagaattaa ataagagttt gtcagatatg gctcaaggat ttcgccgaat
3541 gatgcaatat aaaaaaatta ataaaaatct tgttggtttt atgcgtgcaa cggaagtgac
3601 aataaataat aaagataatt cttataatca gcacatgcat gtattggtat gtgtggaacc
3661 aacttatttt aagaatacag aaaactacgt gaatcaaaaa caatggattc aattttggaa
3721 aaaggcaatg aaattagact atgatccaaa tgtaaaagtt caaatgattc gaccgaaaaa
3781 taaatataaa tcggatatac aatcggcaat tgacgaaact gcaaaatatc ctgtaaagga
3841 tacggatttt atgaccgatg atgaagaaaa gaatttgaaa cgtttgtctg atttggagga
3901 aggtttacac cgtaaaaggt taatctccta tggtggtttg ttaaaagaaa tacataaaaa
3961 attaaacctt gatgacacag aagaaggcga tttgattcat acagatgatg acgaaaaagc
4021 cgatgaagat ggattttcta ttattgcaat gtggaattgg gaacggaaaa attattttat
4081 taaagagtag ttcaacaaac gggccagttt gttgaagatt agatgctata attgttatta
4141 aaaggattga aggatgggag cgctggttac ctatacccag gtagagcctg ccgacttgaa
4201 ggatgaaacc attttgcata cagaggcagg atgtacgtac cgtatccttt tgagcagtat
4261 ctgaataaac acggtatttt ttcagaccct agcctggaat tttggagtat tgaagccatc
4321 aagcagtgtg tcatggctgg gctgggtatt gcgttgctcc cgctagtaac ggtacaaaat
4381 gagctgcgag aaggtaaaat ggcgcgttta gcctgggatg acagtgaaca gcaggtggct
4441 actcaggtcg cttatcacac gaaaaagtgg aaatccccgg ctcttagcga atttttacag
4501 atcgttgagc agcatgtaac acattggcgt gcatgagttc aagccatcac ggcatatgct
4561 tatacaaata atatttttcc ttttcgccta ttcactcaac attcgcccac aacctgcata
4621 catatgtatg gaatgattga ttgtatggag gaggttcaag aatggcatta agtcataaaa
4681 atcgtagaga gattattacc aaagcgatct gtggtaaagg tcgcagattc tctaccgtaa
4741 cccataccgt aactccgccg aataatccga cgagcatttt aggggcatgg attattaacc
4801 accagtatga agctgtggcg gcgggggacg gcattgaggt cgtcgggacg tatgatatca
4861 atatttggta ctcatacgat aaaaactcgc agaccgatgt tgccaaggaa acggtgtcgt
4921 acgtagaaaa tgtgccgctc tcgtatcttg atccgaagca ccgggcgtct acagtggaag
4981 tatccgccga agctacacag gagccgagtt gcgtcgaggc cagtgtgtct tctggaggtg
5041 gcagcgtaat gatccgggtc gagcgggaat ttgcggtgga gctggtggcg gaaacgaaga
5101 ttgttgtaga agtattcccg aatggcagca gcgatgattt tgacaaagac tttgattttg
5161 gagcggaaga gggggactat gaggagctcg accccgacct cattgacgac gagctcggtg
5221 gaggtggtgg aagtggtgga ggtggtggag gtatgcgtaa aggcgaagag ctgttcactg
5281 gtgtcgtccc tattctggtg gaactggatg gtgatgtcaa cggtcataag ttttccgtgc
5341 gtggcgaggg tgaaggtgac gcaactaatg gtaaactgac gctgaagttc atctgtacta
5401 ctggtaaact gccggttcct tggccgactc tggtaacgac gctgacttat ggtgttcagt
5461 gctttgctcg ttatccggac catatgaagc agcatgactt cttcaagtcc gccatgccgg
5521 aaggctatgt gcaggaacgc acgatttcct ttaaggatga cggcacgtac aaaacgcgtg
5581 cggaagtgaa atttgaaggc gataccctgg taaaccgcat tgagctgaaa ggcattgact
5641 ttaaagagga cggcaatatc ctgggccata agctggaata caattttaac agccacaatg
5701 tttacatcac cgccgataaa caaaaaaatg gcattaaagc gaattttaaa attcgccaca
5761 acgtggagga tggcagcgtg cagctggctg atcactacca gcaaaacact ccaatcggtg
5821 atggtcctgt tctgctgcca gacaatcact atctgagcac gcaaagcgtt ctgtctaaag
5881 atccgaacga gaaacgcgat catatggttc tgctggagtt cgtaaccgca gcgggcatca
5941 cgcatggtat ggatgaactg tacaaacatc atcaccacca ccattaatct agagtcgacg
6001 tccccggggc agcccgccta atgagcgggc tttttcacg tcacgcgtcc atggagatct
6061 ttgtctgcaa ctgaaaagtt tataccttac ctggaacaaa tggttgaaac atacgaggct
6121 aatatcggct tattaggaat agtccctgta ctaataaaat caggtggatc agttgatcag
6181 tatattttgg acgaagctcg gaaagaattt ggagatgact tgcttaattc cacaattaaa
6241 ttaagggaaa gaataaagct caagaagaat tctagctag
//

Genbank sequenze of the pHeip plasmid carrying the cotE-TEV_GFP spore display.
LOCUS pHEiPspacZ_TEV_sporedisplayGFP_c-terminal 6297 bp DNA circular UNA 17-APR-2023
DEFINITION .
ACCESSION urn.local...x4-ee4vfx3
VERSION urn.local...x4-ee4vfx3
KEYWORDS .
SOURCE
ORGANISM .

```
FEATURES             Location/Qualifiers
     misc_feature    complement(1..22)
                     /note="Promotor"
                     /standard_name="rpsLp(XC)"
     misc_feature    complement(23..510)
                     /standard_name="oriT"
     primer_bind     complement(117..136)
                     /standard_name="seq_Harms_R"
     oriT            244..353
                     /note="incP origin of transfer"
                     /standard_name="oriT"
     primer_bind     378..402
                     /standard_name="seq_PSG5_F"
     primer_bind     complement(523..553)
                     /standard_name="GG_oriT_R"
     primer_bind     complement(535..549)
                     /standard_name="oriT_XbaI.FOR"
     primer_bind     550..578
                     /standard_name="GG_oriT_F"
     rep_origin      556..637
     rep_origin      complement(592..1180)
     primer_bind     complement(679..696)
                     /standard_name="seq_sg_pCasMC.REV"
     primer_bind     complement(767..786)
                     /standard_name="XbaI_ori_rev"
     primer_bind     1101..1125
                     /standard_name="oriMCSseq2"
     primer_bind     1207..1226
                     /standard_name="kan_fwd"
     primer_bind     complement(1231..1254)
                     /standard_name="GG_ori.FOR"
     primer_bind     complement(1235..1254)
                     /standard_name="pUC_oriMCS_BbsI_fwd"
     primer_bind     complement(1341..1367)
                     /standard_name="GG_Cas9Com2.REV"
     primer_bind     1372..1396
                     /standard_name="GG_neo.FOR"
     primer_bind     1377..1398
                     /standard_name="neo_rev"
     misc_feature    1378..1383
                     /standard_name="-35"
     misc_feature    1400..1408
                     /standard_name="-10"
     misc_feature    1447..2217
                     /note="neo"
                     /standard_name="neo"
     primer_bind     complement(1519..1552)
```

/standard_name="seq_neo.REV"
primer_bind complement(1571..1595)
/standard_name="oriMCSseq1"
primer_bind complement(1832..1849)
/created_by="Max Zander"
/Sequence="GGAACGTTTGGGCTTCTA"
/Tm_(binding_region)="54.5"
/Tm_(with_extension)="68.3"
/%GC_(binding_region)="50.0"
/%GC_(with_extension)="50.0"
/Hairpin_Tm_(with_extension)="59.3"
/Self_Dimer_Tm_(with_extension)="19.4"
/Extension="AAGGGCACAAATCGCATCGT"
/modified_by="Max Zander"
/label="o50XXXBBREV"
primer_bind 1850..1867
/created_by="Max Zander"
/Sequence="ACGATGCGATTTGTGCCC"
/Tm_(binding_region)="58.8"
/Tm_(with_extension)="70.1"
/%GC_(binding_region)="55.6"
/%GC_(with_extension)="56.4"
/Hairpin_Tm_(with_extension)="41.4"
/Self_Dimer_Tm_(with_extension)="None"
/Extension="CGGTAGAAGCCCAAACGTTCC"
/modified_by="Max Zander"
/label="o50XXBBfw"
primer_bind complement(2292..2311)
/standard_name="pUB110_rGI2_ligCtrl_rev"
primer_bind complement(2442..2463)
/standard_name="neo_fwd"
primer_bind 2717..2736
/standard_name="seq_BsaI.FOR"
primer_bind complement(2867..2896)
/standard_name="GG_neo.REV"
primer_bind 2892..2924
/standard_name="GG_bsaI.FOR"
misc_feature 2896
/note="transcription terminator (beta)"
/standard_name="terminator"
misc_feature 3028..3033
/standard_name="-35"
misc_feature 3048..3056
/standard_name="-10"
misc_feature join(3086..3189,3190..4090)
/note="plasmid replication protein from Staph. aureus"
/standard_name="repU"
primer_bind complement(3163..3193)
/standard_name="GG_bsaI.REV"
primer_bind 3190..3220
/standard_name="GG_repU.FOR"
primer_bind 4031..4060
/standard_name="seq_repu.FOR"
misc_feature 4091..4116
/note="transcription terminator"
/standard_name="terminator"

misc_feature 4161..6005
/Original_Bases="AGAAGATTTATTTGAGGTAGCCCTTGCCTACCTAGCTTCCA

AGAAAGATATCCTAACAGCACAAGAGCGGAAAGATGTTTTGTTCTACATCCAGAACAA

CCTCTGCTAAAATTCCTGAAAAATTTTGCAAAAAGTTGTTGACTTTATCTACAAGGTG

TGGCATAATGTGTGGAATTGTGAGCGGATAACAATTAAAGGAGGAAGGATCCACGCAA

GTCTTCTCAGCCGCTACAGGGCGCGTCCCATTCGCCATTCAGGCTGCGCAACTGTTGG

GAAGGGCGATCGGTGCGGGCCTCTTCGCTATTACGCCAGCTGGCGAAAGGGGGATGTG

CTGCAAGGCGATTAAGTTGGGTAACGCCAGGGTTTTCCCAGTCACGACGTTGTAAAAC

GACGGCCAGTGAGCGCGCGTAATACGACTCACTATAGGGCGAATTGGGTACCGGGCCC

CCCCTCGAGGTCCTCCAGCTTTTGTTCCCTTTAGTGAGGGTTAATTGCGCGCTTGGCG

TAATCATGGTCATAGCTGTTTCCTGTGTGAAATTGTTATCCGCTCACAATTCCACACA

ACATACGAGCCGGAAGCATAAAGTGTAAAGCCTGGGGTGCCTAATGAGTGAGCTAACT

CACATTAATTGCGTTGCGCTCACTGCCCGCTTTCCACCGGTGAAGACATGTTT"

/label="AGAAGATTTATTTGAGGTAGCCCTTGCCTACCTAGCTTCCAAGAAAGATA

TCCTAACAGCACAAGAGCGGAAAGATGTTTTGTTCTACATCCAGAACAACCTCTGCTA

AAATTCCTGAAAAATTTTGCAAAAAGTTGTTGACTTTATCTACAAGGTGTGGCATAAT

GTGTGGAATTGTGAGCGGATAACAATTAAAGGAGGAAGGATCCACGCAAGTCTTCTCA

GCCGCTACAGGGCGCGTCCCATTCGCCATTCAGGCTGCGCAACTGTTGGGAAGGGCGA

TCGGTGCGGGCCTCTTCGCTATTACGCCAGCTGGCGAAAGGGGGATGTGCTGCAAGGC

GATTAAGTTGGGTAACGCCAGGGTTTTCCCAGTCACGACGTTGTAAAACGACGGCCAG

TGAGCGCGCGTAATACGACTCACTATAGGGCGAATTGGGTACCGGGCCCCCCCTCGAG

GTCCTCCAGCTTTTGTTCCCTTTAGTGAGGGTTAATTGCGCGCTTGGCGTAATCATGG

TCATAGCTGTTTCCTGTGTGAAATTGTTATCCGCTCACAATTCCACACAACATACGAG

CCGGAAGCATAAAGTGTAAAGCCTGGGGTGCCTAATGAGTGAGCTAACTCACATTAAT
TGCGTTGCGCTCACTGCCCGCTTTCCACCGGTGAAGACATGTTT"
/note="Geneious type: Editing History Replacement"
source 4161
/organism="Genus species"
/mol_type="genomic DNA"
/strain="strain"
unsure 4162..4661
/created_by="mza"
/modified_by="mza"
/label="500bp upstream CotE(nativ prom)"

source <4162..>4661
/organism="Genus species"
/mol_type="genomic DNA"
/strain="strain"
RBS 4650..4654
/created_by="mka"
/label
CDS 4662..>5216
/db_xref="SEED:fig|1406.214.peg.1727"
/product="Outer spore coat protein CotE"
/transl_table=11
/Original_Translation="MALSHKNRREIITKAICGKGRRFSTVTHTVTPPNN

PTSILGAWIINHQYEAVAAGDGIEVVGTYDINIWYSYDKNSQTDVAKETVSYVENVPL

SYLDPKHRASTVEVSAEATQEPSCVEASVSSGGGSVMIRVEREFAVELVAETKIVVEV
FPNGSSDDFDKDFDFGAEEGDYEELDPDLIDDEL"
source <4662..>5216
/mol_type="genomic DNA"
/db_xref="taxon:1406"
/genome_md5="/project=""GRMZ_1406"
/genome_id="1406.214"
/organism="Paenibacillus polymyxa DSM365"
primer_bind 4662..4687
/created_by="mza"
/Sequence="ATGGCATTAAGTCATAAAAATCGTAG"
/Tm_(binding_region)="56.5"
/Tm_(with_extension)="72.1"
/%GC_(binding_region)="30.8"
/%GC_(with_extension)="32.8"
/Extension="CATAAAAACGAAGGGGGATTTTAGGCTTTTACTTA"
/modified_by="mza"
/label="o50031"
primer_bind complement(5198..5216)
/created_by="mza"
/Sequence="GAGCTCGTCGTCAATGAGG"
/Tm_(binding_region)="58.0"
/Tm_(with_extension)="76.4"
/%GC_(binding_region)="57.9"
/%GC_(with_extension)="61.8"
/Hairpin_Tm_(with_extension)="44.3"
/Self_Dimer_Tm_(with_extension)="28.9"
/Extension="ACCTCCACCACCTCCACCACTTCCACCACCTCCACC"
/modified_by="mza"
/label="o50032"
primer_bind complement(5199..5216)
/created_by="Max Zander"
/Sequence="GAGCTCGTCGTCAATGAG"
/Tm_(binding_region)="55.3"
/Tm_(with_extension)="75.3"
/%GC_(binding_region)="55.6"
/%GC_(with_extension)="57.6"
/Hairpin_Tm_(with_extension)="44.3"
/Self_Dimer_Tm_(with_extension)="28.9"
/Extension="AGATTCTCACCACCACCTCCACCACTTCCACCACCTCCACC"
/modified_by="Max Zander"

```
                /label="o50XXXRev"
     linker          5217..>5249
                /created_by="User"
                /modified_by="Max Zander"
                /label="Flex-Linker"
     linker          5250..5270
                /created_by="Max Zander"
                /modified_by="Max Zander"
                /label="TEV recognition site"
     gene            5271..>5984
                /modified_by="User"
                /label="sfGFP(iGEM)"
     primer_bind     5271..5288
                /created_by="Max Zander"
                /Sequence="ATGCGTAAAGGCGAAGAG"
                /Tm_(binding_region)="55.2"
                /Tm_(with_extension)="71.6"
                /%GC_(binding_region)="50.0"
                /%GC_(with_extension)="49.2"
                /Hairpin_Tm_(with_extension)="30.9"
                /Self_Dimer_Tm_(with_extension)="9.4"
                /Extension="GAAGTGGTGGAGGTGGTGGTGAGAATCTTTATTTTCAGGGC"
                /modified_by="Max Zander"
                /label="o50XXXFW"
     primer_bind     complement(5965..5984)
                /created_by="mza"
                /Sequence="TTTGTACAGTTCATCCATAC"
                /Tm_(binding_region)="50.5"
                /Tm_(with_extension)="67.6"
                /%GC_(binding_region)="35.0"
                /%GC_(with_extension)="41.8"
                /Hairpin_Tm_(with_extension)="45.2"
                /Self_Dimer_Tm_(with_extension)="20.0"
                /Extension="ACAAATCCAGATGGAGTATGGTGGTGGTGATGATG"
                /modified_by="mza"
                /label="o50033"
     motif           5985..6002
                /created_by="mza"
                /modified_by="Max Zander"
                /label="HIS-TAG"
     misc_feature    6006..6288
                /created_by="Max Zander"
                /label="rapA"
     primer_bind     complement(6237..6292)
                /standard_name="Fragment.REV"
     primer_bind     6263..12
                /standard_name="Vector.FOR"
     misc_feature    6278..6290
                /standard_name="PsfsE_mcs_rev"
     primer_bind     6278..24
                /standard_name="PsgsE_fw"
     primer_bind     6286..13
                /standard_name="pHEIP_MCS_fw"
     primer_bind     6294..20
                /standard_name="oriT_XbaI.REV"
ORIGIN
```

1 acctgacttc cgcctgcagg gccagctcgc ggacgtgctc atagtccacg acgcccgtga
61 ttttgtagcc ctggccgacg gccagcaggt aggccgacag gctcatgccg gccgccgccg
121 ccttttcctc aatcgctctt cgttcgtctg gaaggcagta caccttgata ggtgggctgc
181 ccttcctggt tggcttggtt tcatcagcca tccgcttgcc ctcatctgtt acgccggcgg
241 tagccggcca gcctcgcaga gcaggattcc cgttgagcac cgccaggtgc gaataaggga
301 cagtgaagaa ggaacacccg ctcgcgggtg ggcctacttc acctatcctg cccggctgac
361 gccgttggat acaccaagga aagtctacac gaaccctttg gcaaaatcct gtatatcgtg
421 cgaaaaagga tggatatacc gaaaaaatcg ctataatgac cccgaagcag ggttatgcag
481 cggaaaagat ccgtcgacct gcatactagc tgctcaaggt cgcccgcaac cggcgcatca
541 agcccgccga ctagtggcca ggaaccgtaa aaaggccgcg ttgctggcgt ttttccatag
601 gctccgcccc cctgacgagc atcacaaaaa tcgacgctca agtcagaggt ggcgaaaccc
661 gacaggacta taaagatacc aggcgtttcc ccctggaagc tccctcgtgc gctctcctgt
721 tccgaccctg ccgcttaccg gatacctgtc cgcctttctc ccttcgggaa gcgtggcgct
781 ttctcatagc tcacgctgta ggtatctcag ttcggtgtag gtcgttcgct ccaagctggg
841 ctgtgtgcac gaaccccccg ttcagcccga ccgctgcgcc ttatccggta actatcgtct
901 tgagtccaac ccggtaagac acgacttatc gccactggca gcagccactg gtaacaggat
961 tagcagagcg aggtatgtag gcggtgctac agagttcttg aagtggtggc ctaactacgg
1021 ctacactaga agaacagtat ttggtatctg cgctctgctg aagccagtta ccttcggaaa
1081 aagagttggt agctcttgat ccggcaaaca aaccaccgct ggtagcggtg gtttttttgt
1141 ttgcaagcag cagattacgc gcagaaaaaa aggatctcaa gaagatcctt tgatcttttc
1201 tacggggtct gacgctcagt ggaacgaaaa ctcacgttaa gggattttgg tcatgagatt
1261 atcaaaaagg atcttcacct agatcctttt ggttcatgtg cagctccatc agcaaaaggg
1321 gatgataagt ttatcaccac cgactatttg caacagtgcc gttaatggtt ataatagctg
1381 aataagaacg gtgctctcca aatattctta tttagaaaag caaatctaaa attatctgaa
1441 aagggaatga gaatagtgaa tggaccaata ataatgacta gagaagaaag aatgaagatt
1501 gttcatgaaa ttaaggaacg aatattggat aaatatgggg atgatgttaa ggctattggt
1561 gtttatggct ctcttggtcg tcagactgat gggccctatt cggatattga gatgatgtgt
1621 gtcatgtcaa cagaggaagc agagttcagc catgaatgga caaccggtga gtggaaggtg
1681 gaagtgaatt ttgatagcga agagattcta ctagattatg catctcaggt ggaatcagat
1741 tggccgctta cacatggtca atttttctct attttgccga tttatgattc aggtggatac
1801 ttagagaaag tgtatcaaac tgctaaatcg gtagaagccc aaacgttcca cgatgcgatt
1861 tgtgccctta tcgtagaaga gctgtttgaa tatgcaggca aatggcgtaa tattcgtgtg
1921 caaggaccga caacatttct accatccttg actgtacagg tagcaatggc aggtgccatg
1981 ttgattggtc tgcatcatcg catctgttat acgacgagcg cttcggtctt aactgaagca
2041 gttaagcaat cagatcttcc ttcaggttat gaccatctgt gccagttcgt aatgtctggt
2101 caactttccg actctgagaa acttctggaa tcgctagaga atttctggaa tgggattcag
2161 gagtggacag aacgacacgg atatatagtg gatgtgtcaa aacgcatacc attttgaacg
2221 atgacctcta ataattgtta atcatgttgg ttacgtattt attaacttct cctagtatta
2281 gtaattatca tggctgtcat ggcgcattaa cggaataaag ggtgtgctta aatcgggcca
2341 ttttgcgtaa taagaaaaag gattaattat gagcgaattg aattaataat aaggtaatag
2401 atttacatta gaaaatgaaa ggggatttta tgcgtgagaa tgttacagtc tatcccggca
2461 ttgccagtcg ggatattaa aaagagtata ggtttttatt gcgataaact aggtttcact
2521 ttggttcacc atgaagatgg attcgcagtt ctaatgtgta atgaggttcg gattcatcta
2581 tgggaggcaa gtgatgaagc tggcgctctc gtagtaatga ttcaccggtt tgtacaggtg
2641 cggagtcgtt tattgctggt actgctagtt gccgcattga agtagaggga attgatgaat
2701 tatatcaaca tattaagcct ttgggcattt tgcaccccaa tacatcatta aaagatcagt
2761 ggtgggatga acgagacttt gcagtaattg atcccgacaa caatttgatt agctttttc
2821 aacaaataaa aagctaaaat ctattattaa tctgttcagc aatcgggcgc gattgctgaa
2881 taaaagatac gaaggtgatg gttttgaact tgttctttct tatcttgata catatagaaa
2941 taacgtcatt tttattttag ttgctgaaag gtgcgttgaa gtgttggtat gtatgtgttt
3001 taaagtattg aaaaccctta aaattggttg cacagaaaaa ccccatctgt taaagttata
3061 agtgactaaa caaataacta aatagatggg ggtttctttt aatattatgt gtcctaatag
3121 tagcatttat tcagatgaaa aatcaagggt tttagtggac aagacaaaaa gtggaaaagt
3181 gaggccatgg agagaaaaga aaatcgctaa tgttgattac tttgaacttc tgcatattct
3241 tgaatttaaa aaggctgaaa gagtaaaaga ttgtgctgaa atattagagt ataaacaaaa
3301 tcgtgaaaca ggcgaaagaa agttgtatcg agtgtggttt tgtaaatcca ggctttgtcc

3361 aatgtgcaac tggaggagag caatgaaaca tggcattcag tcacaaaagg ttgttgctga
3421 agttattaaa caaaagccaa cagttcgttg gttgtttctc acattaacag ttaaaaatgt
3481 ttatgatggc gaagaattaa ataagagttt gtcagatatg gctcaaggat ttcgccgaat
3541 gatgcaatat aaaaaaatta ataaaaatct tgttggtttt atgcgtgcaa cggaagtgac
3601 aataaataat aaagataatt cttataatca gcacatgcat gtattggtat gtgtggaacc
3661 aacttatttt aagaatacag aaaactacgt gaatcaaaaa caatggattc aattttggaa
3721 aaaggcaatg aaattagact atgatccaaa tgtaaaagtt caaatgattc gaccgaaaaa
3781 taaatataaa tcggatatac aatcggcaat tgacgaaact gcaaaatatc ctgtaaagga
3841 tacggatttt atgaccgatg atgaagaaaa gaatttgaaa cgtttgtctg atttggagga
3901 aggtttacac cgtaaaaggt taatctccta tggtggtttg ttaaaagaaa tacataaaaa
3961 attaaacctt gatgacacag aagaaggcga tttgattcat acagatgatg acgaaaaagc
4021 cgatgaagat ggattttcta ttattgcaat gtggaattgg gaacggaaaa attattttat
4081 taaagagtag ttcaacaaac gggccagttt gttgaagatt agatgctata attgttatta
4141 aaaggattga aggatgggag cgctggttac ctatacccag gtagagcctg ccgacttgaa
4201 ggatgaaacc attttgcata cagaggcagg atgtacgtac cgtatccttt tgagcagtat
4261 ctgaataaac acggtatttt ttcagaccct agcctggaat tttggagtat tgaagccatc
4321 aagcagtgtg tcatggctgg gctgggtatt gcgttgctcc cgctagtaac ggtacaaaat
4381 gagctgcgag aaggtaaaat ggcgcgttta gcctgggatg acagtgaaca gcaggtggct
4441 actcaggtcg cttatcacac gaaaaagtgg aaatccccgg ctcttagcga atttttacag
4501 atcgttgagc agcatgtaac acattggcgt gcatgagttc aagccatcac ggcatatgct
4561 tatacaaata atatttttcc ttttcgccta ttcactcaac attcgcccac aacctgcata
4621 catatgtatg gaatgattga ttgtatggag gaggttcaag aatggcatta agtcataaaa
4681 atcgtagaga gattattacc aaagcgatct gtggtaaagg tcgcagattc tctaccgtaa
4741 cccataccgt aactccgccg aataatccga cgagcatttt aggggcatgg attattaacc
4801 accagtatga agctgtggcg gcgggggacg gcattgaggt cgtcgggacg tatgatatca
4861 atatttggta ctcatacgat aaaaactcgc agaccgatgt tgccaaggaa acggtgtcgt
4921 acgtagaaaa tgtgccgctc tcgtatcttg atccgaagca ccgggcgtct acagtggaag
4981 tatccgccga agctacacag gagccgagtt gcgtcgaggc cagtgtgtct tctggaggtg
5041 gcagcgtaat gatccgggtc gagcgggaat ttgcggtgga gctggtggcg gaaacgaaga
5101 ttgttgtaga agtattcccg aatggcagca gcgatgattt tgacaaagac tttgattttg
5161 gagcggaaga gggggactat gaggagctcg accccgacct cattgacgac gagctcggtg
5221 gaggtggtgg aagtggtgga ggtggtggtg agaatcttta ttttcagggc atgcgtaaag
5281 gcgaagagct gttcactggt gtcgtcccta ttctggtgga actggatggt gatgtcaacg
5341 gtcataagtt ttccgtgcgt ggcgagggtg aaggtgacgc aactaatggt aaactgacgc
5401 tgaagttcat ctgtactact ggtaaactgc cggttccttg gccgactctg gtaacgacgc
5461 tgacttatgg tgttcagtgc tttgctcgtt atccggacca tatgaagcag catgacttct
5521 tcaagtccgc catgccggaa ggctatgtgc aggaacgcac gatttccttt aaggatgacg
5581 gcacgtacaa aacgcgtgcg gaagtgaaat ttgaaggcga taccctggta aaccgcattg
5641 agctgaaagg cattgacttt aaagaggacg gcaatatcct gggccataag ctggaataca
5701 attttaacag ccacaatgtt tacatcaccg ccgataaaca aaaaaatggc attaaagcga
5761 attttaaaat tcgccacaac gtggaggatg gcagcgtgca gctggctgat cactaccagc
5821 aaaacactcc aatcggtgat ggtcctgttc tgctgccaga caatcactat ctgagcacgc
5881 aaagcgttct gtctaaagat ccgaacgaga aacgcgatca tatggttctg ctggagttcg
5941 taaccgcagc gggcatcacg catggtatgg atgaactgta caaacatcat caccaccacc
6001 attaatctag agtcgacgtc cccggggcag cccgcctaat gagcgggctt ttttcacgtc
6061 acgcgtccat ggagatcttt gtctgcaact gaaaagttta taccttacct ggaacaaatg
6121 gttgaaacat acgaggctaa tatcggctta ttaggaatag tccctgtact aataaaatca
6181 ggtggatcag ttgatcagta tattttggac gaagctcgga aagaatttgg agatgacttg
6241 cttaattcca caattaaatt aagggaaaga ataaagctca agaagaattc tagctag
//
Genbank sequenze of the pHeip plasmid carrying the cotE-Lip3 spore display
LOCUS p50084 6572 bp DNA circular UNA 03-NOV-2023
DEFINITION pHeip_natprom_cotE_flex_lipase3.
ACCESSION urn.local.kabischlab.c-besnff8
VERSION urn.local.kabischlab.c-besnff8
KEYWORDS .

```
SOURCE
  ORGANISM  .
FEATURES             Location/Qualifiers
     misc_feature    1..4452
                     /note="Geneious type: Editing History Insertion"
                     /standard_name
     misc_feature    1..283
                     /standard_name="rapA"
     primer_bind     complement(232..287)
                     /standard_name="Fragment.REV"
     primer_bind     258..304
                     /standard_name="Vector.FOR"
     misc_feature    273..285
                     /standard_name="PsfsE_mcs_rev"
     primer_bind     273..316
                     /standard_name="PsgsE_fw"
     primer_bind     281..305
                     /standard_name="pHEIP_MCS_fw"
     primer_bind     289..312
                     /standard_name="oriT_XbaI.REV"
     misc_feature    complement(293..314)
                     /note="Promotor"
                     /standard_name="rpsLp(XC)"
     misc_feature    complement(315..802)
                     /standard_name="oriT"
     primer_bind     complement(409..428)
                     /standard_name="seq_Harms_R"
     oriT            536..645
                     /note="incP origin of transfer"
                     /standard_name="oriT"
     primer_bind     670..694
                     /standard_name="seq_PSG5_F"
     primer_bind     complement(815..845)
                     /standard_name="GG_oriT_R"
     primer_bind     complement(827..841)
                     /standard_name="oriT_XbaI.FOR"
     primer_bind     842..870
                     /standard_name="GG_oriT_F"
     rep_origin      848..929
     rep_origin      complement(884..1472)
     primer_bind     complement(971..988)
                     /standard_name="seq_sg_pCasMC.REV"
     primer_bind     complement(1059..1078)
                     /standard_name="XbaI_ori_rev"
     primer_bind     1393..1417
                     /standard_name="oriMCSseq2"
     primer_bind     1499..1518
                     /standard_name="kan_fwd"
     primer_bind     complement(1523..1546)
                     /standard_name="GG_ori.FOR"
     primer_bind     complement(1527..1546)
                     /standard_name="pUC_oriMCS_BbsI_fwd"
     primer_bind     complement(1633..1659)
                     /standard_name="GG_Cas9Com2.REV"
     primer_bind     1664..1688
                     /standard_name="GG_neo.FOR"
```

```
primer_bind     1669..1690
                /standard_name="neo_rev"
misc_feature    1670..1675
                /standard_name="-35"
misc_feature    1692..1700
                /standard_name="-10"
misc_feature    1739..2509
                /note="neo"
                /standard_name="neo"
primer_bind     complement(1811..1844)
                /standard_name="seq_neo.REV"
primer_bind     complement(1863..1887)
                /standard_name="oriMCSseq1"
primer_bind     complement(2584..2603)
                /standard_name="pUB110_rGI2_ligCtrl_rev"
primer_bind     complement(2734..2755)
                /standard_name="neo_fwd"
primer_bind     3009..3028
                /standard_name="seq_BsaI.FOR"
primer_bind     complement(3159..3188)
                /standard_name="GG_neo.REV"
primer_bind     3184..3216
                /standard_name="GG_bsaI.FOR"
misc_feature    3188
                /note="transcription terminator (beta)"
                /standard_name="terminator"
misc_feature    3320..3325
                /standard_name="-35"
misc_feature    3340..3348
                /standard_name="-10"
misc_feature    join(3378..3481,3482..4382)
                /note="plasmid replication protein from Staph. aureus"
                /standard_name="repU"
primer_bind     complement(3455..3485)
                /standard_name="GG_bsaI.REV"
primer_bind     3482..3512
                /standard_name="GG_repU.FOR"
primer_bind     4323..4352
                /standard_name="seq_repu.FOR"
misc_feature    4383..4408
                /note="transcription terminator"
                /standard_name="terminator"
source          4453
                /organism="Genus species"
                /mol_type="genomic DNA"
                /strain="strain"
unsure          4454..4955
source          <4454..>4955
                /organism="Genus species"
                /mol_type="genomic DNA"
                /strain="strain"
misc_feature    4544
                /note="Geneious type: Editing History Insertion"
                /standard_name
misc_feature    4872
                /note="Geneious type: Editing History Insertion"
```

/standard_name
RBS 4944..4948
CDS 4956..>5510
/db_xref="SEED:fig|1406.214.peg.1727"
/product="Outer spore coat protein CotE"
/transl_table=11
source <4956..>5510
/mol_type="genomic DNA"
/db_xref="taxon:1406"
/organism="Paenibacillus polymyxa DSM365"
primer_bind 4956..4981
/standard_name="o50031"
primer_bind complement(5492..5510)
/standard_name="o50032"
misc_feature 5511..5546
/note="Geneious type: linker"
/standard_name="Flex-Linker"
CDS 5547..>6551
/db_xref="SEED:fig|1406.265.peg.4275"
/db_xref="GO:0004622"
/db_xref="GO:0047372"
/product="Lysophospholipase (EC 3.1.1.5); Monoglyceride
lipase (EC 3.1.1.23)"
/EC_number="3.1.1.5"
/EC_number="3.1.1.23"
/transl_table=11
/standard_name="Lysophospholipase (EC 3.1.1.5);
Monoglyceride lipase (EC 3.1.1.23) CDS(Lipase3)"
source <5547..>6551
/mol_type="genomic DNA"
/db_xref="taxon:1406"
/organism="Paenibacillus polymyxa DSM365"
primer_bind 5547..5565
/standard_name="o50554"
misc_feature 5548..6551
/note="Geneious type: Editing History Replacement"
/standard_name="GCCTGCGAAAGGGCGCTTTCTTCCTTATGCTGAGCGGCTGAC

TTCACCTGCTCATGCGCTTGGTAGGAGCTGCGGACCAACGGTCCAGATTCAACGTGGC

TGAATCCCCGCTGTAATCCCTCTTCCTTTAACTTCGCAAAATCTTCCGGTGGATAATA

TTTTTGCACATATAAATGTTTCTCGGACGGCTGCAAATATTGACCAATTGTCAGAATG

TCACAATCGACTTTACGCAGATCATCCATGGCTTGTAAGATCTCGTCCCACTCTTCTC

CGACACCTAGCATGATGCTTGATTTCGTTGGAATAGCCGGATTTAATTGCTTCGCACG

TTGAAGCAGCTCCAGAGAGCGACGGTATTTCGCTTTAGCCCGAACCTTGTCGGACATG

CGCTCCACCGTTTCAATATTATGATTCAGAATATCTGGTTTGGCATCCATGACGATTC

GCAGCGATTCAATATCGCCCATAAAATCGGGAATCAATACTTCTACACTGCACAAAGG

TAACCGGCCGCGAATCGCACGAACCGTTTCTGCAAATATAGTAGCGCCCCCATCTTTT

AAATCGTCGCGAGCCACACTGGTTACGACACAGTGCTTCAAATTCATGTTCCCAGCCG

CTTCCGCGACGCGATCAGGCTCCTGCAAGTCAAGTTCCGTAGGCAATCCCGTATTTAC

TGCGCAAAAACGACACGCACGCGTACAAATATCACCCAAAATCATAAATGTAGCTGTC

CTATTGGCCCAGCATTCATATATATTAGGGCATCGTGCCTCCTCACATACGGTATGTA

ATGTCTTGGAACGCATCATGCTCTTGATTTCTTGATAGTTATCGCCTGTCGTAAGTTT
AATACGAATCCAGTCGGGCTTAGCTTCCTTCTCTTTTCTAGACAA"
primer_bind complement(6533..6551)
/standard_name="o50555_rev_lip"
misc_feature 6552..6569
/standard_name="HIS-TAG"
ORIGIN
1 tctagagtcg acgtccccgg ggcagcccgc ctaatgagcg ggcttttttc acgtcacgcg
61 tccatggaga tctttgtctg caactgaaaa gtttatacct tacctggaac aaatggttga
121 aacatacgag gctaatatcg gcttattagg aatagtccct gtactaataa aatcaggtgg
181 atcagttgat cagtatattt tggacgaagc tcggaaagaa tttggagatg acttgcttaa
241 ttccacaatt aaattaaggg aaagaataaa gctcaagaag aattctagct agacctgact
301 tccgcctgca gggccagctc gcggacgtgc tcatagtcca cgacgcccgt gattttgtag
361 ccctggccga cggccagcag gtaggccgac aggctcatgc cggccgccgc cgcctttcc
421 tcaatcgctc ttcgttcgtc tggaaggcag tacaccttga taggtgggct gcccttcctg
481 gttggcttgg tttcatcagc catccgcttg ccctcatctg ttacgccggc ggtagccggc
541 cagcctcgca gagcaggatt cccgttgagc accgccaggt gcgaataagg gacagtgaag
601 aaggaacacc cgctcgcggg tgggcctact tcacctatcc tgcccggctg acgccgttgg
661 atacaccaag gaaagtctac acgaaccctt tggcaaaatc ctgtatatcg tgcgaaaaag
721 gatggatata ccgaaaaaat cgctataatg accccgaagc agggttatgc agcggaaaag
781 atccgtcgac ctgcatacta gctgctcaag gtcgcccgca accggcgcat caagcccgcc
841 gactagtggc caggaaccgt aaaaaggccg cgttgctggc gtttttccat aggctccgcc
901 cccctgacga gcatcacaaa aatcgacgct caagtcagag gtggcgaaac ccgacaggac
961 tataaagata ccaggcgttt ccccctggaa gctccctcgt gcgctctcct gttccgaccc
1021 tgccgcttac cggatacctg tccgcctttc tcccttcggg aagcgtggcg ctttctcata
1081 gctcacgctg taggtatctc agttcggtgt aggtcgttcg ctccaagctg ggctgtgtgc
1141 acgaaccccc cgttcagccc gaccgctgcg ccttatccgg taactatcgt cttgagtcca
1201 acccggtaag acacgactta tcgccactgg cagcagccac tggtaacagg attagcagag
1261 cgaggtatgt aggcggtgct acagagttct tgaagtggtg gcctaactac ggctacacta
1321 gaagaacagt atttggtatc tgcgctctgc tgaagccagt taccttcgga aaaagagttg
1381 gtagctcttg atccggcaaa caaaccaccg ctggtagcgg tggttttttt gtttgcaagc
1441 agcagattac gcgcagaaaa aaaggatctc aagaagatcc tttgatcttt tctacggggt
1501 ctgacgctca gtggaacgaa aactcacgtt aagggatttt ggtcatgaga ttatcaaaaa
1561 ggatcttcac ctagatcctt ttggttcatg tgcagctcca tcagcaaaag gggatgataa
1621 gtttatcacc accgactatt tgcaacagtg ccgttaatgg ttataatagc tgaataagaa
1681 cggtgctctc caaatattct tatttagaaa agcaaatcta aaattatctg aaaagggaat
1741 gagaatagtg aatggaccaa taataatgac tagagaagaa agaatgaaga ttgttcatga
1801 aattaaggaa cgaatattgg ataaatatgg ggatgatgtt aaggctattg gtgtttatgg
1861 ctctcttggt cgtcagactg atgggcccta ttcggatatt gagatgatgt gtgtcatgtc
1921 aacagaggaa gcagagttca gccatgaatg gacaaccggt gagtggaagg tggaagtgaa
1981 ttttgatagc gaagagattc tactagatta tgcatctcag gtggaatcag attggccgct
2041 tacacatggt caatttttct ctattttgcc gatttatgat tcaggtggat acttagagaa
2101 agtgtatcaa actgctaaat cggtagaagc ccaaacgttc cacgatgcga tttgtgccct
2161 tatcgtagaa gagctgtttg aatatgcagg caaatggcgt aatattcgtg tgcaaggacc
2221 gacaacattt ctaccatcct tgactgtaca ggtagcaatg gcaggtgcca tgttgattgg
2281 tctgcatcat cgcatctgtt atacgacgag cgcttcggtc ttaactgaag cagttaagca
2341 atcagatctt ccttcaggtt atgaccatct gtgccagttc gtaatgtctg gtcaactttc

2401 cgactctgag aaacttctgg aatcgctaga gaatttctgg aatgggattc aggagtggac
2461 agaacgacac ggatatatag tggatgtgtc aaaacgcata ccattttgaa cgatgacctc
2521 taataattgt taatcatgtt ggttacgtat ttattaactt ctcctagtat tagtaattat
2581 catggctgtc atggcgcatt aacggaataa agggtgtgct taaatcgggc cattttgcgt
2641 aataagaaaa aggattaatt atgagcgaat tgaattaata ataaggtaat agatttacat
2701 tagaaaatga aaggggattt tatgcgtgag aatgttacag tctatcccgg cattgccagt
2761 cggggatatt aaaaagagta taggttttta ttgcgataaa ctaggtttca ctttggttca
2821 ccatgaagat ggattcgcag ttctaatgtg taatgaggtt cggattcatc tatgggaggc
2881 aagtgatgaa gctggcgctc tcgtagtaat gattcaccgg tttgtacagg tgcggagtcg
2941 tttattgctg gtactgctag ttgccgcatt gaagtagagg gaattgatga attatatcaa
3001 catattaagc ctttgggcat tttgcacccc aatacatcat taaaagatca gtggtgggat
3061 gaacgagact ttgcagtaat tgatcccgac aacaatttga ttagcttttt tcaacaaata
3121 aaaagctaaa atctattatt aatctgttca gcaatcgggc gcgattgctg aataaaagat
3181 acgaaggtga tggttttgaa cttgttcttt cttatcttga tacatataga aataacgtca
3241 tttttatttt agttgctgaa aggtgcgttg aagtgttggt atgtatgtgt tttaaagtat
3301 tgaaaaccct taaaattggt tgcacagaaa aaccccatct gttaaagtta taagtgacta
3361 aacaaataac taaatagatg ggggtttctt ttaatattat gtgtcctaat agtagcattt
3421 attcagatga aaaatcaagg gttttagtgg acaagacaaa aagtggaaaa gtgaggccat
3481 ggagagaaaa gaaaatcgct aatgttgatt actttgaact tctgcatatt cttgaattta
3541 aaaaggctga aagagtaaaa gattgtgctg aaatattaga gtataaacaa aatcgtgaaa
3601 caggcgaaag aaagttgtat cgagtgtggt tttgtaaatc caggctttgt ccaatgtgca
3661 actggaggag agcaatgaaa catggcattc agtcacaaaa ggttgttgct gaagttatta
3721 aacaaaagcc aacagttcgt tggttgtttc tcacattaac agttaaaaat gtttatgatg
3781 gcgaagaatt aaataagagt ttgtcagata tggctcaagg atttcgccga atgatgcaat
3841 ataaaaaaat taataaaaat cttgttggtt ttatgcgtgc aacggaagtg acaataaata
3901 ataaagataa ttcttataat cagcacatgc atgtattggt atgtgtggaa ccaacttatt
3961 ttaagaatac agaaaactac gtgaatcaaa aacaatggat tcaattttgg aaaaaggcaa
4021 tgaaattaga ctatgatcca aatgtaaaag ttcaaatgat tcgaccgaaa aataaatata
4081 aatcggatat acaatcggca attgacgaaa ctgcaaaata tcctgtaaag gatacggatt
4141 ttatgaccga tgatgaagaa aagaatttga aacgtttgtc tgatttggag gaaggtttac
4201 accgtaaaag gttaatctcc tatggtggtt tgttaaaaga aatacataaa aaattaaacc
4261 ttgatgacac agaagaaggc gatttgattc atacagatga tgacgaaaaa gccgatgaag
4321 atggattttc tattattgca atgtggaatt gggaacggaa aaattatttt attaaagagt
4381 agttcaacaa acgggccagt ttgttgaaga ttagatgcta taattgttat taaaaggatt
4441 gaaggatggg agcgctggtt acctataccc aggtagagcc tgccgacttg aaggatgaaa
4501 ccattttgca tacagaggca ggatgtacgt accgtatcct ttttgagcag tatctgaata
4561 aacacggtat tttttcagac cctagcctgg aattttggag tattgaagcc atcaagcagt
4621 gtgtcatggc tgggctgggt attgcgttgc tcccgctagt aacggtacaa aatgagctgc
4681 gagaaggtaa aatggcgcgt ttagcctggg atgacagtga acagcaggtg gctactcagg
4741 tcgcttatca cacgaaaaag tggaaatccc cggctcttag cgaattttta cagatcgttg
4801 agcagcatgt aacacattgg cgtgcatgag ttcaagccat cacggcatat gcttatacaa
4861 ataatatttt ttccttttcg cctattcact caacattcgc ccacaacctg catacatatg
4921 tatggaatga ttgattgtat ggaggaggtt caagaatggc attaagtcat aaaaatcgta
4981 gagagattat taccaaagcg atctgtggta aaggtcgcag attctctacc gtaacccata
5041 ccgtaactcc gccgaataat ccgacgagca ttttaggggc atggattatt aaccaccagt
5101 atgaagctgt ggcggcgggg gacggcattg aggtcgtcgg gacgtatgat atcaatattt
5161 ggtactcata cgataaaaac tcgcagaccg atgttgccaa ggaaacggtg tcgtacgtag
5221 aaaatgtgcc gctctcgtat cttgatccga agcaccgggc gtctacagtg gaagtatccg
5281 ccgaagctac acaggagccg agttgcgtcg aggccagtgt gtcttctgga ggtggcagcg
5341 taatgatccg ggtcgagcgg gaatttgcgg tggagctggt ggcggaaacg aagattgttg
5401 tagaagtatt cccgaatggc agcagcgatg attttgacaa agactttgat tttggagcgg
5461 aagaggggga ctatgaggag ctcgaccccg acctcattga cgacgagctc ggtggaggtg
5521 gtggaagtgg tggaggtggt ggaggtatgc gggaatacac cttcaccatc gctgaaaatg
5581 acggaacaga gctttttgcc taccgctggc tgcctgatca gaatttgccc attaagggga
5641 ttgttcaaat ttcgcatggt atgtgtgaga cgtcctatcg gtacatccga ctggccgaaa
5701 agcttactgc ttgcggttac ggtgtgtatg ccaacgatca tatcggtcac gggcgcacgg

```
    5761 ctggtgatcc tgataagctg ggtatgccag gagctgatgc gtttaatcga atggcaaatg
    5821 gcatgctgga gctaggcgaa attgtggcta aggaattccc tgaacagtct cgtttttgc
    5881 tgggtcacag catggggtcc tttttaaccc agaaaattat gtatgacgat cggcagacgt
    5941 atcatgggtt tattttatcg ggaaccaacg gaagacgtgg tcttctaaaa ctcggagagc
    6001 aagttgctct actgcaagcc aaactgcaag gaatggatca tcgcagtatg ttgctcaacg
    6061 ctatggtctt tggaggcttt aatcgtgcct ttcgtccggt gcgcacagcg ttcgactggc
    6121 tgtcccgtga tcctgaggaa gtagatcagt tcgtgcatga cccattatgt ggagctattt
    6181 gcacgacagg cttttttctg gattttttca ggctattgca agaaatccat tggccttcct
    6241 cgctgaaaaa tatcaatccc aaactgccag tttatatttt tgccggggat cgtgatccag
    6301 tgggtttgtt cggtaaaggc gtgttgtcac tagtagaaat gtatcgaagc ctagagcttc
    6361 aggacataga gtatcgcctc tatcctgatg gtcgccatga gatgcttcat gaaacaaacc
    6421 gcgacgaggt gatgagtgac atcgtagact ggctcgaccg tcatgttaat acagaggtga
    6481 cctctgtttc cacattgtct ggagcctccg atgaacctga acaatcaacc tcgcccaagt
    6541 catcagcctt gcatcatcac caccaccatt aa
//
```

Genbank sequenze of the pHeip plasmid carrying the cotE-pnbA spore display

```
LOCUS       p50085                7046 bp    DNA     circular UNA 20-SEP-2023
DEFINITION  pHeip_natprom_cotE_flex_pnbA.
ACCESSION   urn.local.kabischlab.c-besnff8
VERSION     urn.local.kabischlab.c-besnff8
KEYWORDS    .
SOURCE
  ORGANISM  .
FEATURES             Location/Qualifiers
     misc_feature    1..4452
                     /note="Geneious type: Editing History Insertion"
                     /standard_name
     misc_feature    1..283
                     /standard_name="rapA"
     primer_bind     complement(232..287)
                     /standard_name="Fragment.REV"
     primer_bind     258..304
                     /standard_name="Vector.FOR"
     misc_feature    273..285
                     /standard_name="PsfsE_mcs_rev"
     primer_bind     273..316
                     /standard_name="PsgsE_fw"
     primer_bind     281..305
                     /standard_name="pHEIP_MCS_fw"
     primer_bind     289..312
                     /standard_name="oriT_XbaI.REV"
     misc_feature    complement(293..314)
                     /note="Promotor"
                     /standard_name="rpsLp(XC)"
     misc_feature    complement(315..802)
                     /standard_name="oriT"
     primer_bind     complement(409..428)
                     /standard_name="seq_Harms_R"
     oriT            536..645
                     /note="incP origin of transfer"
                     /standard_name="oriT"
     primer_bind     670..694
                     /standard_name="seq_PSG5_F"
     primer_bind     complement(815..845)
                     /standard_name="GG_oriT_R"
     primer_bind     complement(827..841)
```

/standard_name="oriT_XbaI.FOR"
primer_bind   842..870
/standard_name="GG_oriT_F"
rep_origin   848..929
rep_origin   complement(884..1472)
primer_bind   complement(971..988)
/standard_name="seq_sg_pCasMC.REV"
primer_bind   complement(1059..1078)
/standard_name="XbaI_ori_rev"
primer_bind   1393..1417
/standard_name="oriMCSseq2"
primer_bind   1499..1518
/standard_name="kan_fwd"
primer_bind   complement(1523..1546)
/standard_name="GG_ori.FOR"
primer_bind   complement(1527..1546)
/standard_name="pUC_oriMCS_BbsI_fwd"
primer_bind   complement(1633..1659)
/standard_name="GG_Cas9Com2.REV"
primer_bind   1664..1688
/standard_name="GG_neo.FOR"
primer_bind   1669..1690
/standard_name="neo_rev"
misc_feature   1670..1675
/standard_name="-35"
misc_feature   1692..1700
/standard_name="-10"
misc_feature   1739..2509
/note="neo"
/standard_name="neo"
primer_bind   complement(1811..1844)
/standard_name="seq_neo.REV"
primer_bind   complement(1863..1887)
/standard_name="oriMCSseq1"
primer_bind   complement(2584..2603)
/standard_name="pUB110_rGI2_ligCtrl_rev"
primer_bind   complement(2734..2755)
/standard_name="neo_fwd"
primer_bind   3009..3028
/standard_name="seq_BsaI.FOR"
primer_bind   complement(3159..3188)
/standard_name="GG_neo.REV"
primer_bind   3184..3216
/standard_name="GG_bsaI.FOR"
misc_feature   3188
/note="transcription terminator (beta)"
/standard_name="terminator"
misc_feature   3320..3325
/standard_name="-35"
misc_feature   3340..3348
/standard_name="-10"
misc_feature   join(3378..3481,3482..4382)
/note="plasmid replication protein from Staph. aureus"
/standard_name="repU"
primer_bind   complement(3455..3485)
/standard_name="GG_bsaI.REV"

primer_bind 3482..3512
/standard_name="GG_repU.FOR"
primer_bind 4323..4352
/standard_name="seq_repu.FOR"
misc_feature 4383..4408
/note="transcription terminator"
/standard_name="terminator"
source 4453
/organism="Genus species"
/mol_type="genomic DNA"
/strain="strain"
unsure 4454..4955
source <4454..>4955
/organism="Genus species"
/mol_type="genomic DNA"
/strain="strain"
misc_feature 4544
/note="Geneious type: Editing History Insertion"
/standard_name
misc_feature 4872
/note="Geneious type: Editing History Insertion"
/standard_name
RBS 4944..4948
CDS 4956..>5510
/db_xref="SEED:fig|1406.214.peg.1727"
/product="Outer spore coat protein CotE"
/transl_table=11
source <4956..>5510
/mol_type="genomic DNA"
/db_xref="taxon:1406"
/organism="Paenibacillus polymyxa DSM365"
primer_bind 4956..4981
/standard_name="o50031"
primer_bind complement(5492..5510)
/standard_name="o50032"
misc_feature 5511..5546
/note="Geneious type: linker"
/standard_name="Flex-Linker"
CDS 5547..>7025
/db_xref="SEED:fig|1406.265.peg.4431"
/product="para-nitrobenzyl esterase (intracellular
esterase B)"
/transl_table=11
source complement(<5547..>7025)
/mol_type="genomic DNA"
/db_xref="taxon:1406"
/organism="Paenibacillus polymyxa DSM365"
primer_bind 5547..5569
/standard_name="o50557"
misc_feature 5548..7024
/note="Geneious type: Editing History Replacement"
/standard_name="GCCTGCGAAAGGGCGCTTTCTTCCTTATGCTGAGCGGCTGAC

TTCACCTGCTCATGCGCTTGGTAGGAGCTGCGGACCAACGGTCCAGATTCAACGTGGC

TGAATCCCCGCTGTAATCCCTCTTCCTTTAACTTCGCAAAATCTTCCGGTGGATAATA

TTTTTGCACATATAAATGTTTCTCGGACGGCTGCAAATATTGACCAATTGTCAGAATG

TCACAATCGACTTTACGCAGATCATCCATGGCTTGTAAGATCTCGTCCCACTCTTCTC

CGACACCTAGCATGATGCTTGATTTCGTTGGAATAGCCGGATTTAATTGCTTCGCACG

TTGAAGCAGCTCCAGAGAGCGACGGTATTTCGCTTTAGCCCGAACCTTGTCGGACATG

CGCTCCACCGTTTCAATATTATGATTCAGAATATCTGGTTTGGCATCCATGACGATTC

GCAGCGATTCAATATCGCCCATAAAATCGGGAATCAATACTTCTACACTGCACAAAGG

TAACCGGCCGCGAATCGCACGAACCGTTTCTGCAAATATAGTAGCGCCCCCATCTTTT

AAATCGTCGCGAGCCACACTGGTTACGACACAGTGCTTCAAATTCATGTTCCCAGCCG

CTTCCGCGACGCGATCAGGCTCCTGCAAGTCAAGTTCCGTAGGCAATCCCGTATTTAC

TGCGCAAAAACGACACGCACGCGTACAAATATCACCCAAAATCATAAATGTAGCTGTC

CTATTGGCCCAGCATTCATATATATTAGGGCATCGTGCCTCCTCACATACGGTATGTA

ATGTCTTGGAACGCATCATGCTCTTGATTTCTTGATAGTTATCGCCTGTCGTAAGTTT
AATACGAATCCAGTCGGGCTTAGCTTCCTTCTCTTTTCTAGACA"
primer_bind complement(7008..7025)
/standard_name="o50556"
misc_feature 7026..7043
/standard_name="HIS-TAG"
ORIGIN
1 tctagagtcg acgtccccgg ggcagcccgc ctaatgagcg ggcttttttc acgtcacgcg
61 tccatggaga tctttgtctg caactgaaaa gtttatacct tacctggaac aaatggttga
121 aacatacgag gctaatatcg gcttattagg aatagtccct gtactaataa aatcaggtgg
181 atcagttgat cagtatattt tggacgaagc tcggaaagaa tttggagatg acttgcttaa
241 ttccacaatt aaattaaggg aaagaataaa gctcaagaag aattctagct agacctgact
301 tccgcctgca gggccagctc gcggacgtgc tcatagtcca cgacgcccgt gattttgtag
361 ccctggccga cggccagcag gtaggccgac aggctcatgc cggccgccgc cgccttttcc
421 tcaatcgctc ttcgttcgtc tggaaggcag tacaccttga taggtgggct gcccttcctg
481 gttggcttgg tttcatcagc catccgcttg ccctcatctg ttacgccggc ggtagccggc
541 cagcctcgca gagcaggatt cccgttgagc accgccaggt gcgaataagg gacagtgaag
601 aaggaacacc cgctcgcggg tgggcctact tcacctatcc tgcccggctg acgccgttgg
661 atacaccaag gaaagtctac acgaaccctt tggcaaaatc ctgtatatcg tgcgaaaaag
721 gatggatata ccgaaaaaat cgctataatg accccgaagc agggttatgc agcggaaaag
781 atccgtcgac ctgcatacta gctgctcaag gtcgcccgca accggcgcat caagcccgcc
841 gactagtggc caggaaccgt aaaaaggccg cgttgctggc gtttttccat aggctccgcc
901 cccctgacga gcatcacaaa aatcgacgct caagtcagag gtggcgaaac ccgacaggac
961 tataaagata ccaggcgttt ccccctggaa gctccctcgt gcgctctcct gttccgaccc
1021 tgccgcttac cggatacctg tccgcctttc tcccttcggg aagcgtggcg ctttctcata
1081 gctcacgctg taggtatctc agttcggtgt aggtcgttcg ctccaagctg ggctgtgtgc
1141 acgaaccccc cgttcagccc gaccgctgcg ccttatccgg taactatcgt cttgagtcca
1201 acccggtaag acacgactta tcgccactgg cagcagccac tggtaacagg attagcagag
1261 cgaggtatgt aggcggtgct acagagttct tgaagtggtg gcctaactac ggctacacta
1321 gaagaacagt atttggtatc tgcgctctgc tgaagccagt taccttcgga aaaagagttg
1381 ggtagctcttg atccggcaaa caaaccaccg ctggtagcgg tggttttttt gtttgcaagc
1441 agcagattac gcgcagaaaa aaaggatctc aagaagatcc tttgatcttt tctacggggt
1501 ctgacgctca gtggaacgaa aactcacgtt aagggatttt ggtcatgaga ttatcaaaaa

1561 ggatcttcac ctagatcctt ttggttcatg tgcagctcca tcagcaaaag gggatgataa
1621 gtttatcacc accgactatt tgcaacagtg ccgttaatgg ttataatagc tgaataagaa
1681 cggtgctctc caaatattct tatttagaaa agcaaatcta aaattatctg aaaagggaat
1741 gagaatagtg aatggaccaa taataatgac tagagaagaa agaatgaaga ttgttcatga
1801 aattaaggaa cgaatattgg ataaatatgg ggatgatgtt aaggctattg gtgtttatgg
1861 ctctcttggt cgtcagactg atgggcccta ttcggatatt gagatgatgt gtgtcatgtc
1921 aacagaggaa gcagagttca gccatgaatg gacaaccggt gagtggaagg tggaagtgaa
1981 tttgatagc gaagagattc tactagatta tgcatctcag gtggaatcag attggccgct
2041 tacacatggt caatttttct ctattttgcc gatttatgat tcaggtggat acttagagaa
2101 agtgtatcaa actgctaaat cggtagaagc ccaaacgttc cacgatgcga tttgtgccct
2161 tatcgtagaa gagctgtttg aatatgcagg caaatggcgt aatattcgtg tgcaaggacc
2221 gacaacattt ctaccatcct tgactgtaca ggtagcaatg gcaggtgcca tgttgattgg
2281 tctgcatcat cgcatctgtt atacgacgag cgcttcggtc ttaactgaag cagttaagca
2341 atcagatctt ccttcaggtt atgaccatct gtgccagttc gtaatgtctg gtcaactttc
2401 cgactctgag aaacttctgg aatcgctaga gaatttctgg aatgggattc aggagtggac
2461 agaacgacac ggatatatag tggatgtgtc aaaacgcata ccattttgaa cgatgacctc
2521 taataattgt taatcatgtt ggttacgtat ttattaactt ctcctagtat tagtaattat
2581 catggctgtc atggcgcatt aacggaataa agggtgtgct taaatcgggc cattttgcgt
2641 aataagaaaa aggattaatt atgagcgaat tgaattaata ataaggtaat agatttacat
2701 tagaaaatga aaggggattt tatgcgtgag aatgttacag tctatcccgg cattgccagt
2761 cggggatatt aaaaagagta taggttttta ttgcgataaa ctaggtttca ctttggttca
2821 ccatgaagat ggattcgcag ttctaatgtg taatgaggtt cggattcatc tatgggaggc
2881 aagtgatgaa gctggcgctc tcgtagtaat gattcaccgg tttgtacagg tgcggagtcg
2941 tttattgctg gtactgctag ttgccgcatt gaagtagagg gaattgatga attatatcaa
3001 catattaagc ctttgggcat tttgcacccc aatacatcat taaaagatca gtggtgggat
3061 gaacgagact ttgcagtaat tgatcccgac aacaatttga ttagcttttt tcaacaaata
3121 aaaagctaaa atctattatt aatctgttca gcaatcgggc gcgattgctg aataaaagat
3181 acgaaggtga tggttttgaa cttgttcttt cttatcttga tacatataga aataacgtca
3241 tttttatttt agttgctgaa aggtgcgttg aagtgttggt atgtatgtgt tttaaagtat
3301 tgaaaaccct taaaattggt tgcacagaaa aaccccatct gttaaagtta taagtgacta
3361 aacaaataac taaatagatg gggtttctt ttaatattat gtgtcctaat agtagcattt
3421 attcagatga aaaatcaagg gttttagtgg acaagacaaa aagtggaaaa gtgaggccat
3481 ggagagaaaa gaaaatcgct aatgttgatt actttgaact tctgcatatt cttgaattta
3541 aaaaggctga aagagtaaaa gattgtgctg aaatattaga gtataaacaa aatcgtgaaa
3601 caggcgaaag aaagttgtat cgagtgtggt tttgtaaatc caggctttgt ccaatgtgca
3661 actggaggag agcaatgaaa catggcattc agtcacaaaa ggttgttgct gaagttatta
3721 aacaaaagcc aacagttcgt tggttgtttc tcacattaac agttaaaaat gtttatgatg
3781 gcgaagaatt aaataagagt ttgtcagata tggctcaagg atttcgccga atgatgcaat
3841 ataaaaaaat taataaaaat cttgttggtt ttatgcgtgc aacggaagtg acaataaata
3901 ataaagataa ttcttataat cagcacatgc atgtattggt atgtgtggaa ccaacttatt
3961 ttaagaatac agaaaactac gtgaatcaaa aacaatggat tcaattttgg aaaaaggcaa
4021 tgaaattaga ctatgatcca aatgtaaaag ttcaaatgat tcgaccgaaa aataaatata
4081 aatcggatat acaatcggca attgacgaaa ctgcaaaata tcctgtaaag gatacggatt
4141 ttatgaccga tgatgaagaa aagaatttga aacgtttgtc tgatttggag gaaggtttac
4201 accgtaaaag gttaatctcc tatggtggtt tgttaaaaga aatacataaa aaattaaacc
4261 ttgatgacac agaagaaggc gatttgattc atacagatga tgacgaaaaa gccgatgaag
4321 atggattttc tattattgca atgtggaatt gggaacggaa aaattatttt attaaagagt
4381 agttcaacaa acgggccagt ttgttgaaga ttagatgcta taattgttat taaaaggatt
4441 gaaggatggg agcgctggtt acctataccc aggtagagcc tgccgacttg aaggatgaaa
4501 ccattttgca tacagaggca ggatgtacgt accgtatcct ttttgagcag tatctgaata
4561 aacacggtat tttttcagac cctagcctgg aattttggag tattgaagcc atcaagcagt
4621 gtgtcatggc tgggctgggt attgcgttgc tcccgctagt aacggtacaa aatgagctgc
4681 gagaaggtaa aatggcgcgt ttagcctggg atgacagtga acagcaggtg gctactcagg
4741 tcgcttatca cacgaaaaag tggaaatccc cggctcttag cgaattttta cagatcgttg
4801 agcagcatgt aacacattgg cgtgcatgag ttcaagccat cacggcatat gcttatacaa
4861 ataatatttt ttcctttttcg cctattcact caacattcgc ccacaacctg catacatatg

4921 tatggaatga ttgattgtat ggaggaggtt caagaatggc attaagtcat aaaaatcgta
4981 gagagattat taccaaagcg atctgtggta aaggtcgcag attctctacc gtaacccata
5041 ccgtaactcc gccgaataat ccgacgagca ttttaggggc atggattatt aaccaccagt
5101 atgaagctgt ggcggcgggg gacggcattg aggtcgtcgg gacgtatgat atcaatattt
5161 ggtactcata cgataaaaac tcgcagaccg atgttgccaa ggaaacggtg tcgtacgtag
5221 aaaatgtgcc gctctcgtat cttgatccga agcaccgggc gtctacagtg gaagtatccg
5281 ccgaagctac acaggagccg agttgcgtcg aggccagtgt gtcttctgga ggtggcagcg
5341 taatgatccg ggtcgagcgg gaatttgcgg tggagctggt ggcggaaacg aagattgttg
5401 tagaagtatt cccgaatggc agcagcgatg attttgacaa agactttgat tttggagcgg
5461 aagaggggga ctatgaggag ctcgaccccg acctcattga cgacgagctc ggtggaggtg
5521 gtggaagtgg tggaggtggt ggaggtatgg agagtattac agtacatact cgtttaggtc
5581 aattacgtgg ggaaacgggg aatggatatc atgtatggaa aggtatccca tatgcacagc
5641 ctcctgttgg aaaactacgt tttcacgcgc cccagccttt ggagccttgg gagggtgtac
5701 gggctgcgac gagttttggg ccaatctgtc cgcaacctat gccatccgct gaaagtatga
5761 ctggaaatct ggtggaaccg cctgagcagt cagaggattg cttatacctg aacatttgga
5821 cacctgcttc gaaggctcct gagaagggac gtccggtgat ggtgtggatt catggcggta
5881 cctttgtcac ggggtccggg attatacctt tatatgacgg ggaacgaatg gcgaagaatg
5941 gcgatgttgt ggttgttacg atcaattatc gattagggcc gttaggattt ttgcatctaa
6001 ctccaaaggg agatggcctg acctctaatg cgggactgct ggatcagatc gctgcgctgg
6061 aatgggttag ggatcacatc tctgcctttg gcggtaaccc ggacgaagta acggtgttcg
6121 gtgaatcggc aggcgcgatg agtattgccg ctttattggc gatgccagcc gctgagggac
6181 tgttccagcg tgcgattttg cagagcggtg cgtcgcaggt attgccaacc tcacaggccg
6241 agcaagtgac ggctgtgtat ctacagcagc tgggtgtgga cactcagcat ccagagaggt
6301 tgtttagcct gccgacagat gcgctgatgc tagcgatggc gaagacgcat gaagtcatcg
6361 gaccagggat ggcgatgatc tatcaaccca ttgtggacgg tgtgaccttg ccagatgtac
6421 ccctgtctgc aatcgcgcaa ggttcagcaa agcaggtatc cgttttgatt ggaacgaact
6481 tgcatgaggg ggcctatttc atccgaaaag aatcccatct aatgaacaag tcaacagcga
6541 gacaggcatt ggaaatgatg acgggcatgt cagacatcgg cgacctgata gaaccatttc
6601 cggttacgat tgagggacag gcgcaaatgt tgaccgatct atttttttgg cgtcctgcgt
6661 tggctcttgc tgttgcacag tcggcgcatg cgcctgtatg gatgtatcgt tttgattgga
6721 cattgcccgg gcatcctacc tttgaacaag cggttcacgg tgcagagatt gcgtttgttt
6781 tcgataatct ggagctgcta gataagcttg gattagagat tcaatcttcg atgcagaagc
6841 tggctcaaga tatgcagcag gcatgggttg cttttgcacg tgatgggaag ccagtgctgt
6901 ccgagggggc ttggccgatg tacgacagag aagagcgaac tacagctatt ttccatcaga
6961 atattaaggt ggagcacgac cctgaaggag atagacgccg tcatttaacc ggtcagatga
7021 ccctacatca tcaccaccac cattaa

//

Genbank sequenze of the pHeip plasmid carrying the cotE-LipA spore display

LOCUS p50086 6203 bp DNA circular UNA 11-NOV-2023
DEFINITION pHeip_natprom_cotE_flex_lipABsub.
ACCESSION urn.local.kabischlab.c-besnff8
VERSION urn.local.kabischlab.c-besnff8
KEYWORDS .
SOURCE
ORGANISM .
FEATURES Location/Qualifiers
misc_feature 1..4452
/note="Geneious type: Editing History Insertion"
/standard_name
misc_feature 1..283
/standard_name="rapA"
primer_bind complement(232..287)
/standard_name="Fragment.REV"
primer_bind 258..304
/standard_name="Vector.FOR"
misc_feature 273..285

/standard_name="PsfsE_mcs_rev"
primer_bind 273..316
/standard_name="PsgsE_fw"
primer_bind 281..305
/standard_name="pHEIP_MCS_fw"
primer_bind 289..312
/standard_name="oriT_XbaI.REV"
misc_feature complement(293..314)
/note="Promotor"
/standard_name="rpsLp(XC)"
misc_feature complement(315..802)
/standard_name="oriT"
primer_bind complement(409..428)
/standard_name="seq_Harms_R"
oriT 536..645
/note="incP origin of transfer"
/standard_name="oriT"
primer_bind 670..694
/standard_name="seq_PSG5_F"
primer_bind complement(815..845)
/standard_name="GG_oriT_R"
primer_bind complement(827..841)
/standard_name="oriT_XbaI.FOR"
primer_bind 842..870
/standard_name="GG_oriT_F"
rep_origin 848..929
rep_origin complement(884..1472)
primer_bind complement(971..988)
/standard_name="seq_sg_pCasMC.REV"
primer_bind complement(1059..1078)
/standard_name="XbaI_ori_rev"
primer_bind 1393..1417
/standard_name="oriMCSseq2"
primer_bind 1499..1518
/standard_name="kan_fwd"
primer_bind complement(1523..1546)
/standard_name="GG_ori.FOR"
primer_bind complement(1527..1546)
/standard_name="pUC_oriMCS_BbsI_fwd"
primer_bind complement(1633..1659)
/standard_name="GG_Cas9Com2.REV"
primer_bind 1664..1688
/standard_name="GG_neo.FOR"
primer_bind 1669..1690
/standard_name="neo_rev"
misc_feature 1670..1675
/standard_name="-35"
misc_feature 1692..1700
/standard_name="-10"
misc_feature 1739..2509
/note="neo"
/standard_name="neo"
primer_bind complement(1811..1844)
/standard_name="seq_neo.REV"
primer_bind complement(1863..1887)
/standard_name="oriMCSseq1"

primer_bind complement(2584..2603)
/standard_name="pUB110_rGI2_ligCtrl_rev"
primer_bind complement(2734..2755)
/standard_name="neo_fwd"
primer_bind 3009..3028
/standard_name="seq_BsaI.FOR"
primer_bind complement(3159..3188)
/standard_name="GG_neo.REV"
primer_bind 3184..3216
/standard_name="GG_bsaI.FOR"
misc_feature 3188
/note="transcription terminator (beta)"
/standard_name="terminator"
misc_feature 3320..3325
/standard_name="-35"
misc_feature 3340..3348
/standard_name="-10"
misc_feature join(3378..3481,3482..4382)
/note="plasmid replication protein from Staph. aureus"
/standard_name="repU"
primer_bind complement(3455..3485)
/standard_name="GG_bsaI.REV"
primer_bind 3482..3512
/standard_name="GG_repU.FOR"
primer_bind 4323..4352
/standard_name="seq_repu.FOR"
misc_feature 4383..4408
/note="transcription terminator"
/standard_name="terminator"
source 4453
/organism="Genus species"
/mol_type="genomic DNA"
/strain="strain"
unsure 4454..4955
source <4454..>4955
/organism="Genus species"
/mol_type="genomic DNA"
/strain="strain"
misc_feature 4544
/note="Geneious type: Editing History Insertion"
/standard_name
misc_feature 4872
/note="Geneious type: Editing History Insertion"
/standard_name
RBS 4944..4948
CDS 4956..>5510
/db_xref="SEED:fig|1406.214.peg.1727"
/product="Outer spore coat protein CotE"
/transl_table=11
source <4956..>5510
/mol_type="genomic DNA"
/db_xref="taxon:1406"
/organism="Paenibacillus polymyxa DSM365"
primer_bind 4956..4981
/standard_name="o50031"
primer_bind complement(5492..5510)

```
                /standard_name="o50032"
     misc_feature    5511..5546
                /note="Geneious type: linker"
                /standard_name="Flex-Linker"
     gene            5547..>6182
                /gene="estA"
                /locus_tag="BSU_02700"
                /old_locus_tag="BSU02700"
                /db_xref="GeneID:938389"
                /standard_name="LipA gene"
     source          <5547..>6182
                /organism="Bacillus subtilis subsp. subtilis str. 168"
                /mol_type="genomic DNA"
                /strain=168
                /sub_species="subtilis"
                /type_material="type strain of Bacillus subtilis"
                /db_xref="taxon:224308"
     primer_bind     5547..5569
                /standard_name="o50559"
     misc_feature    5548..6182
                /note="Geneious type: Editing History Replacement"
                /standard_name="GCCTGCGAAAGGGCGCTTTCTTCCTTATGCTGAGCGGCTGAC

TTCACCTGCTCATGCGCTTGGTAGGAGCTGCGGACCAACGGTCCAGATTCAACGTGGC

TGAATCCCCGCTGTAATCCCTCTTCCTTTAACTTCGCAAAATCTTCCGGTGGATAATA

TTTTTGCACATATAAATGTTTCTCGGACGGCTGCAAATATTGACCAATTGTCAGAATG

TCACAATCGACTTTACGCAGATCATCCATGGCTTGTAAGATCTCGTCCCACTCTTCTC

CGACACCTAGCATGATGCTTGATTTCGTTGGAATAGCCGGATTTAATTGCTTCGCACG

TTGAAGCAGCTCCAGAGAGCGACGGTATTTCGCTTTAGCCCGAACCTTGTCGGACATG

CGCTCCACCGTTTCAATATTATGATTCAGAATATCTGGTTTGGCATCCATGACGATTC

GCAGCGATTCAATATCGCCCATAAAATCGGGAATCAATACTTCTACACTGCACAAAGG

TAACCGGCCGCGAATCGCACGAACCGTTTCTGCAAATATAGTAGCGCCCCCATCTTTT

AAATCGTCGCGAGCCACACTGGTTACGACACAGTGCTTCAAATTCATGTTCCCAGCCG

CTTCCGCGACGCGATCAGGCTCCTGCAAGTCAAGTTCCGTAGGCAATCCCGTATTTAC

TGCGCAAAAACGACACGCACGCGTACAAATATCACCCAAAATCATAAATGTAGCTGTC

CTATTGGCCCAGCATTCATATATATTAGGGCATCGTGCCTCCTCACATACGGTATGTA

ATGTCTTGGAACGCATCATGCTCTTGATTTCTTGATAGTTATCGCCTGTCGTAAGTTT
                AATACGAATCCAGTCGGGCTTAGCTTCCTTCTCTTTTCTAGACAA"
     primer_bind     complement(6166..6182)
                /standard_name="o50558"
     misc_feature    6183..6200
                /standard_name="HIS-TAG"
ORIGIN
```

1 tctagagtcg acgtccccgg ggcagcccgc ctaatgagcg ggctttttc acgtcacgcg
61 tccatggaga tctttgtctg caactgaaaa gtttatacct tacctggaac aaatggttga
121 aacatacgag gctaatatcg gcttattagg aatagtccct gtactaataa aatcaggtgg
181 atcagttgat cagtatattt tggacgaagc tcggaaagaa tttggagatg acttgcttaa
241 ttccacaatt aaattaaggg aaagaataaa gctcaagaag aattctagct agacctgact
301 tccgcctgca gggccagctc gcggacgtgc tcatagtcca cgacgcccgt gattttgtag
361 ccctggccga cggccagcag gtaggccgac aggctcatgc cggccgccgc cgccttttcc
421 tcaatcgctc ttcgttcgtc tggaaggcag tacaccttga taggtgggct gcccttcctg
481 gttggcttgg tttcatcagc catccgcttg ccctcatctg ttacgccggc ggtagccggc
541 cagcctcgca gagcaggatt cccgttgagc accgccaggt gcgaataagg gacagtgaag
601 aaggaacacc cgctcgcggg tgggcctact tcacctatcc tgcccggctg acgccgttgg
661 atacaccaag gaaagtctac acgaaccctt tggcaaaatc ctgtatatcg tgcgaaaaag
721 gatggatata ccgaaaaaat cgctataatg accccgaagc agggttatgc agcggaaaag
781 atccgtcgac ctgcatacta gctgctcaag gtcgcccgca accggcgcat caagcccgcc
841 gactagtggc caggaaccgt aaaaaggccg cgttgctggc gtttttccat aggctccgcc
901 cccctgacga gcatcacaaa aatcgacgct caagtcagag gtggcgaaac ccgacaggac
961 tataaagata ccaggcgttt ccccctggaa gctccctcgt gcgctctcct gttccgaccc
1021 tgccgcttac cggatacctg tccgcctttc tcccttcggg aagcgtggcg ctttctcata
1081 gctcacgctg taggtatctc agttcggtgt aggtcgttcg ctccaagctg ggctgtgtgc
1141 acgaaccccc cgttcagccc gaccgctgcg ccttatccgg taactatcgt cttgagtcca
1201 acccggtaag acacgactta tcgccactgg cagcagccac tggtaacagg attagcagag
1261 cgaggtatgt aggcggtgct acagagttct tgaagtggtg gcctaactac ggctacacta
1321 gaagaacagt atttggtatc tgcgctctgc tgaagccagt taccttcgga aaaagagttg
1381 gtagctcttg atccggcaaa caaaccaccg ctggtagcgg tggttttttt gtttgcaagc
1441 agcagattac gcgcagaaaa aaaggatctc aagaagatcc tttgatcttt tctacggggt
1501 ctgacgctca gtggaacgaa aactcacgtt aagggatttt ggtcatgaga ttatcaaaaa
1561 ggatcttcac ctagatcctt ttggttcatg tgcagctcca tcagcaaaag gggatgataa
1621 gtttatcacc accgactatt tgcaacagtg ccgttaatgg ttataatagc tgaataagaa
1681 cggtgctctc caaatattct tatttagaaa agcaaatcta aaattatctg aaaagggaat
1741 gagaatagtg aatggaccaa taataatgac tagagaagaa agaatgaaga ttgttcatga
1801 aattaaggaa cgaatattgg ataaatatgg ggatgatgtt aaggctattg gtgtttatgg
1861 ctctcttggt cgtcagactg atgggcccta ttcggatatt gagatgatgt gtgtcatgtc
1921 aacagaggaa gcagagttca gccatgaatg gacaaccggt gagtggaagg tggaagtgaa
1981 ttttgatagc gaagagattc tactagatta tgcatctcag gtggaatcag attggccgct
2041 tacacatggt caatttttct ctattttgcc gatttatgat tcaggtggat acttagagaa
2101 agtgtatcaa actgctaaat cggtagaagc ccaaacgttc cacgatgcga tttgtgccct
2161 tatcgtagaa gagctgtttg aatatgcagg caaatggcgt aatattcgtg tgcaaggacc
2221 gacaacattt ctaccatcct tgactgtaca ggtagcaatg gcaggtgcca tgttgattgg
2281 tctgcatcat cgcatctgtt atacgacgag cgcttcggtc ttaactgaag cagttaagca
2341 atcagatctt ccttcaggtt atgaccatct gtgccagttc gtaatgtctg gtcaactttc
2401 cgactctgag aaacttctgg aatcgctaga gaatttctgg aatgggattc aggagtggac
2461 agaacgacac ggatatatag tggatgtgtc aaaacgcata ccattttgaa cgatgacctc
2521 taataattgt taatcatgtt ggttacgtat ttattaactt ctcctagtat tagtaattat
2581 catggctgtc atggcgcatt aacggaataa agggtgtgct taaatcgggc cattttgcgt
2641 aataagaaaa aggattaatt atgagcgaat tgaattaata ataaggtaat agatttacat
2701 tagaaaatga aaggggattt tatgcgtgag aatgttacag tctatcccgg cattgccagt
2761 cggggatatt aaaaagagta taggttttta ttgcgataaa ctaggtttca ctttggttca
2821 ccatgaagat ggattcgcag ttctaatgtg taatgaggtt cggattcatc tatgggaggc
2881 aagtgatgaa gctggcgctc tcgtagtaat gattcaccgg tttgtacagg tgcggagtcg
2941 tttattgctg gtactgctag ttgccgcatt gaagtagagg gaattgatga attatatcaa
3001 catattaagc ctttgggcat tttgcacccc aatacatcat taaaagatca gtggtgggat
3061 gaacgagact ttgcagtaat tgatcccgac aacaatttga ttagcttttt tcaacaaata
3121 aaaagctaaa atctattatt aatctgttca gcaatcgggc gcgattgctg aataaaagat
3181 acgaaggtga tggttttgaa cttgttcttt cttatcttga tacatataga aataacgtca
3241 tttttatttt agttgctgaa aggtgcgttg aagtgttggt atgtatgtgt tttaaagtat
3301 tgaaaaccct taaaattggt tgcacagaaa aaccccatct gttaaagtta taagtgacta

3361 aacaaataac taaatagatg ggggtttctt ttaatattat gtgtcctaat agtagcattt
3421 attcagatga aaaatcaagg gttttagtgg acaagacaaa aagtggaaaa gtgaggccat
3481 ggagagaaaa gaaaatcgct aatgttgatt actttgaact tctgcatatt cttgaattta
3541 aaaaggctga aagagtaaaa gattgtgctg aaatattaga gtataaacaa aatcgtgaaa
3601 caggcgaaag aaagttgtat cgagtgtggt tttgtaaatc caggctttgt ccaatgtgca
3661 actggaggag agcaatgaaa catggcattc agtcacaaaa ggttgttgct gaagttatta
3721 aacaaaagcc aacagttcgt tggttgtttc tcacattaac agttaaaaat gtttatgatg
3781 gcgaagaatt aaataagagt ttgtcagata tggctcaagg atttcgccga atgatgcaat
3841 ataaaaaaat taataaaaat cttgttggtt ttatgcgtgc aacggaagtg acaataaata
3901 ataaagataa ttcttataat cagcacatgc atgtattggt atgtgtggaa ccaacttatt
3961 ttaagaatac agaaaactac gtgaatcaaa aacaatggat tcaattttgg aaaaaggcaa
4021 tgaaattaga ctatgatcca aatgtaaaag ttcaaatgat tcgaccgaaa aataaatata
4081 aatcggatat acaatcggca attgacgaaa ctgcaaaata tcctgtaaag gatacggatt
4141 ttatgaccga tgatgaagaa aagaatttga aacgtttgtc tgatttggag gaaggtttac
4201 accgtaaaag gttaatctcc tatggtggtt tgttaaaaga aatacataaa aaattaaacc
4261 ttgatgacac agaagaaggc gatttgattc atacagatga tgacgaaaaa gccgatgaag
4321 atggattttc tattattgca atgtggaatt gggaacggaa aaattatttt attaaagagt
4381 agttcaacaa acgggccagt ttgttgaaga ttagatgcta taattgttat taaaaggatt
4441 gaaggatggg agcgctggtt acctataccc aggtagagcc tgccgacttg aaggatgaaa
4501 ccattttgca tacagaggca ggatgtacgt accgtatcct ttttgagcag tatctgaata
4561 aacacggtat tttttcagac cctagcctgg aattttggag tattgaagcc atcaagcagt
4621 gtgtcatggc tgggctgggt attgcgttgc tcccgctagt aacggtacaa aatgagctgc
4681 gagaaggtaa aatggcgcgt ttagcctggg atgacagtga acagcaggtg gctactcagg
4741 tcgcttatca cacgaaaaag tggaaatccc cggctcttag cgaattttta cagatcgttg
4801 agcagcatgt aacacattgg cgtgcatgag ttcaagccat cacggcatat gcttatacaa
4861 ataatatttt ttccttttcg cctattcact caacattcgc ccacaacctg catacatatg
4921 tatggaatga ttgattgtat ggaggaggtt caagaatggc attaagtcat aaaaatcgta
4981 gagagattat taccaaagcg atctgtggta aaggtcgcag attctctacc gtaacccata
5041 ccgtaactcc gccgaataat ccgacgagca ttttaggggc atggattatt aaccaccagt
5101 atgaagctgt ggcggcgggg gacggcattg aggtcgtcgg gacgtatgat atcaatattt
5161 ggtactcata cgataaaaac tcgcagaccg atgttgccaa ggaaacggtg tcgtacgtag
5221 aaaatgtgcc gctctcgtat cttgatccga agcaccgggc gtctacagtg gaagtatccg
5281 ccgaagctac acaggagccg agttgcgtcg aggccagtgt gtcttctgga ggtggcagcg
5341 taatgatccg ggtcgagcgg gaatttgcgg tggagctggt ggcggaaacg aagattgttg
5401 tagaagtatt cccgaatggc agcagcgatg attttgacaa agactttgat tttggagcgg
5461 aagaggggga ctatgaggag ctcgaccccg acctcattga cgacgagctc ggtggaggtg
5521 gtggaagtgg tggaggtggt ggaggtatga aatttgtaaa aagaaggatc attgcacttg
5581 taacaatttt gatgctgtct gttacatcgc tgtttgcgtt gcagccgtca gcaaaagccg
5641 ctgaacacaa tccagtcgtt atggttcacg gtattggagg ggcatcattc aattttgcgg
5701 gaattaagag ctatctcgta tctcagggct ggtcgcggga caagctgtat gcagttgatt
5761 tttgggacaa gacaggcaca aattataaca atggaccggt attatcacga tttgtgcaaa
5821 aggttttaga tgaaacgggt gcgaaaaaag tggatattgt cgctcacagc atgggggggcg
5881 cgaacacact ttactacata aaaaatctgg acggcggaaa taaagttgca aacgtcgtga
5941 cgcttggcgg cgcgaaccgt ttgacgacag gcaaggcgct tccgggaaca gatccaaatc
6001 aaaagatttt atacacatcc atttacagca gtgccgatat gattgtcatg aattacttat
6061 caagattaga tggtgctaga aacgttcaaa tccatggcgt tggacacatc ggccttctgt
6121 acagcagcca agtcaacagc ctgattaaag aagggctgaa cggcgggggc cagaatacga
6181 atcatcatca ccaccaccat taa
//
LOCUS p50080 6465 bp DNA circular UNA 19-DEC-2022
DEFINITION pHeip_natprom_cotE_lipoylA .
ACCESSION urn.local.kabischlab.c-besnff8
VERSION urn.local.kabischlab.c-besnff8
KEYWORDS .
SOURCE
ORGANISM .

```
FEATURES             Location/Qualifiers
     misc_feature    1..4452
                     /Original_Bases
                     /label
                     /note="Geneious type: Editing History Insertion"
     misc_feature    1..283
                     /created_by="Max Zander"
                     /label="rapA"
     primer_bind     complement(232..287)
                     /standard_name="Fragment.REV"
     primer_bind     258..304
                     /standard_name="Vector.FOR"
     misc_feature    273..285
                     /standard_name="PsfsE_mcs_rev"
     primer_bind     273..316
                     /standard_name="PsgsE_fw"
     primer_bind     281..305
                     /standard_name="pHEIP_MCS_fw"
     primer_bind     289..312
                     /standard_name="oriT_XbaI.REV"
     misc_feature    complement(293..314)
                     /note="Promotor"
                     /standard_name="rpsLp(XC)"
     misc_feature    complement(315..802)
                     /standard_name="oriT"
     primer_bind     complement(409..428)
                     /standard_name="seq_Harms_R"
     oriT            536..645
                     /note="incP origin of transfer"
                     /standard_name="oriT"
     primer_bind     670..694
                     /standard_name="seq_PSG5_F"
     primer_bind     complement(815..845)
                     /standard_name="GG_oriT_R"
     primer_bind     complement(827..841)
                     /standard_name="oriT_XbaI.FOR"
     primer_bind     842..870
                     /standard_name="GG_oriT_F"
     rep_origin      848..929
     rep_origin      complement(884..1472)
     primer_bind     complement(971..988)
                     /standard_name="seq_sg_pCasMC.REV"
     primer_bind     complement(1059..1078)
                     /standard_name="XbaI_ori_rev"
     primer_bind     1393..1417
                     /standard_name="oriMCSseq2"
     primer_bind     1499..1518
                     /standard_name="kan_fwd"
     primer_bind     complement(1523..1546)
                     /standard_name="GG_ori.FOR"
     primer_bind     complement(1527..1546)
                     /standard_name="pUC_oriMCS_BbsI_fwd"
     primer_bind     complement(1633..1659)
                     /standard_name="GG_Cas9Com2.REV"
     primer_bind     1664..1688
                     /standard_name="GG_neo.FOR"
```

```
primer_bind     1669..1690
                /standard_name="neo_rev"
misc_feature    1670..1675
                /standard_name="-35"
misc_feature    1692..1700
                /standard_name="-10"
misc_feature    1739..2509
                /note="neo"
                /standard_name="neo"
primer_bind     complement(1811..1844)
                /standard_name="seq_neo.REV"
primer_bind     complement(1863..1887)
                /standard_name="oriMCSseq1"
primer_bind     complement(2584..2603)
                /standard_name="pUB110_rGI2_ligCtrl_rev"
primer_bind     complement(2734..2755)
                /standard_name="neo_fwd"
primer_bind     3009..3028
                /standard_name="seq_BsaI.FOR"
primer_bind     complement(3159..3188)
                /standard_name="GG_neo.REV"
primer_bind     3184..3216
                /standard_name="GG_bsaI.FOR"
misc_feature    3188
                /note="transcription terminator (beta)"
                /standard_name="terminator"
misc_feature    3320..3325
                /standard_name="-35"
misc_feature    3340..3348
                /standard_name="-10"
misc_feature    join(3378..3481,3482..4382)
                /note="plasmid replication protein from Staph. aureus"
                /standard_name="repU"
primer_bind     complement(3455..3485)
                /standard_name="GG_bsaI.REV"
primer_bind     3482..3512
                /standard_name="GG_repU.FOR"
primer_bind     4323..4352
                /standard_name="seq_repu.FOR"
misc_feature    4383..4408
                /note="transcription terminator"
                /standard_name="terminator"
source          4453
                /organism="Genus species"
                /mol_type="genomic DNA"
                /strain="strain"
unsure          4454..4953
                /created_by="mza"
                /modified_by="mza"
                /label="500bp upstream CotE(nativ prom)"
source          <4454..>4953
                /organism="Genus species"
                /mol_type="genomic DNA"
                /strain="strain"
RBS             4942..4946
                /created_by="mka"
```

/label
CDS 4954..>5508
/db_xref="SEED:fig|1406.214.peg.1727"
/product="Outer spore coat protein CotE"
/transl_table=11
/Original_Translation="MALSHKNRREIITKAICGKGRRFSTVTHTVTPPNN

PTSILGAWIINHQYEAVAAGDGIEVVGTYDINIWYSYDKNSQTDVAKETVSYVENVPL

SYLDPKHRASTVEVSAEATQEPSCVEASVSSGGGSVMIRVEREFAVELVAETKIVVEV
FPNGSSDDFDKDFDFGAEEGDYEELDPDLIDDEL"
source <4954..>5508
/mol_type="genomic DNA"
/db_xref="taxon:1406"
/genome_md5="/project=""GRMZ_1406"
/genome_id="1406.214"
/organism="Paenibacillus polymyxa DSM365"
primer_bind 4954..4979
/created_by="mza"
/Sequence="ATGGCATTAAGTCATAAAAATCGTAG"
/Tm_(binding_region)="56.5"
/Tm_(with_extension)="72.1"
/%GC_(binding_region)="30.8"
/%GC_(with_extension)="32.8"
/Extension="CATAAAAACGAAGGGGGATTTTAGGCTTTTACTTA"
/modified_by="mza"
/label="o50031"
primer_bind complement(5490..5508)
/created_by="mza"
/Sequence="GAGCTCGTCGTCAATGAGG"
/Tm_(binding_region)="58.0"
/Tm_(with_extension)="76.4"
/%GC_(binding_region)="57.9"
/%GC_(with_extension)="61.8"
/Hairpin_Tm_(with_extension)="44.3"
/Self_Dimer_Tm_(with_extension)="28.9"
/Extension="ACCTCCACCACCTCCACCACTTCCACCACCTCCACC"
/modified_by="mza"
/label="o50032"
linker 5509..5544
/created_by="User"
/modified_by="mza"
/label="Flex-Linker"
CDS 5545..>6444
/db_xref="SEED:fig|1406.265.peg.1435"
/product="Lipoyl synthase (EC 2.8.1.8)"
/EC_number="2.8.1.8"
/transl_table=11
/Original_Translation="MSRKEKEAKPDWIRIKLTTGDNYQEIKSMMRSKTL

HTVCEEARCPNIYECWANRTATFMILGDICTRACRFCAVNTGLPTELDLQEPDRVAEA

AGNMNLKHCVVTSVARDDLKDGGATIFAETVRAIRGRLPLCSVEVLIPDFMGDIESLR

IVMDAKPDILNHNIETVERMSDKVRAKAKYRRSLELLQRAKQLNPAIPTKSSIMLGVG

EEWDEILQAMDDLRKVDCDILTIGQYLQPSEKHLYVQKYYPPEDFAKLKEEGLQRGFS
HVESGPLVRSSYQAHEQVKSAAQHKEESALSQA"
source <5545..>6444
/mol_type="genomic DNA"
/db_xref="taxon:1406"
/genome_md5="/project=""GRA_1406"
/genome_id="1406.265"
/organism="Paenibacillus polymyxa DSM365"
primer_bind 5545..5566
/created_by="Max Zander"
/Sequence="TTGTCTAGAAAAGAGAAGGAAG"
/Tm_(binding_region)="53.2"
/Tm_(with_extension)="69.8"
/%GC_(binding_region)="36.4"
/%GC_(with_extension)="50.0"
/Hairpin_Tm_(with_extension)="32.8"
/Self_Dimer_Tm_(with_extension)="None"
/Extension="TGGTGGAAGTGGTGGAGGTGGTGGAGGT"
/modified_by="Max Zander"
/label="o50371"
primer_bind complement(6427..6444)
/created_by="Max Zander"
/Sequence="AGCCTGCGAAAGGGCGCT"
/Tm_(binding_region)="65.1"
/Tm_(with_extension)="71.8"
/%GC_(binding_region)="66.7"
/%GC_(with_extension)="52.0"
/Hairpin_Tm_(with_extension)="52.1"
/Self_Dimer_Tm_(with_extension)="5.5"
/Extension="TCGACTCTAGATTAATGGTGGTGGTGATGATG"
/modified_by="Max Zander"
/label="o50372"
misc_feature 6445..6462
/created_by="mza"
/modified_by="mza"
/label="HIS-TAG"
misc_feature 6463..6465
/Original_Bases="ACTCCATCTGGATTTGTTCAGAACGCTCGGTTGCCGCCGGG

CGTTTTTTATCTAGCTAGACCTGACTTCCGCCTGCAGGGCCAGCTCGCGGACGTGCTC

ATAGTCCACGACGCCCGTGATTTTGTAGCCCTGGCCGACGGCCAGCAGGTAGGCCGAC

AGGCTCATGCCGGCCGCCGCCGCCTTTTCCTCAATCGCTCTTCGTTCGTCTGGAAGGC

AGTACACCTTGATAGGTGGGCTGCCCTTCCTGGTTGGCTTGGTTTCATCAGCCATCCG

CTTGCCCTCATCTGTTACGCCGGCGGTAGCCGGCCAGCCTCGCAGAGCAGGATTCCCG

TTGAGCACCGCCAGGTGCGAATAAGGGACAGTGAAGAAGGAACACCCGCTCGCGGGTG

GGCCTACTTCACCTATCCTGCCCGGCTGACGCCGTTGGATACACCAAGGAAAGTCTAC

ACGAACCCTTTGGCAAAATCCTGTATATCGTGCGAAAAAGGATGGATATACCGAAAAA

ATCGCTATAATGACCCCGAAGCAGGGTTATGCAGCGGAAAAGATCCGTCGACCTGCAT

ACTAGCTGCTCAAGGTCGCCCGCAACCGGCGCATCAAGCCCGCCGACTAGTGGCCAGG

AACCGTAAAAAGGCCGCGTTGCTGGCGTTTTTCCATAGGCTCCGCCCCCCTGACGAGC

ATCACAAAAATCGACGCTCAAGTCAGAGGTGGCGAAACCCGACAGGACTATAAAGATA

CCAGGCGTTTCCCCCTGGAAGCTCCCTCGTGCGCTCTCCTGTTCCGACCCTGCCGCTT

ACCGGATACCTGTCCGCCTTTCTCCCTTCGGGAAGCGTGGCGCTTTCTCATAGCTCAC

GCTGTAGGTATCTCAGTTCGGTGTAGGTCGTTCGCTCCAAGCTGGGCTGTGTGCACGA

CCCCCCGTTCAGCCCGACCGCTGCGCCTTATCCGGTAACTATCGTCTTGAGTCCAAC

CCGGTAAGACACGACTTATCGCCACTGGCAGCAGCCACTGGTAACAGGATTAGCAGAG

CGAGGTATGTAGGCGGTGCTACAGAGTTCTTGAAGTGGTGGCCTAACTACGGCTACAC

TAGAAGAACAGTATTTGGTATCTGCGCTCTGCTGAAGCCAGTTACCTTCGGAAAAAGA

GTTGGTAGCTCTTGATCCGGCAAACAAACCACCGCTGGTAGCGGTGGTTTTTTTGTTT

GCAAGCAGCAGATTACGCGCAGAAAAAAAGGATCTCAAGAAGATCCTTTGATCTTTTC

TACGGGGTCTGACGCTCAGTGGAACGAAAACTCACGTTAAGGGATTTTGGTCATGAGA

TTATCAAAAAGGATCTTCACCTAGATCCTTTTGGTTCATGTGCAGCTCCATCAGCAAA

AGGGGATGATAAGTTTATCACCACCGACTATTTGCAACAGTGCCGTTAATGGTTATAA

TAGCTGAATAAGAACGGTGCTCTCCAAATATTCTTATTTAGAAAAGCAAATCTAAAAT

TATCTGAAAAGGGAATGAGAATAGTGAATGGACCAATAATAATGACTAGAGAAGAAAG

AATGAAGATTGTTCATGAAATTAAGGAACGAATATTGGATAAATATGGGGATGATGTT

AAGGCTATTGGTGTTTATGGCTCTCTTGGTCGTCAGACTGATGGGCCCTATTCGGATA

TTGAGATGATGTGTGTCATGTCAACAGAGGAAGCAGAGTTCAGCCATGAATGGACAAC

CGGTGAGTGGAAGGTGGAAGTGAATTTTGATAGCGAAGAGATTCTACTAGATTATGCA

TCTCAGGTGGAATCAGATTGGCCGCTTACACATGGTCAATTTTTCTCTATTTTGCCGA

TTTATGATTCAGGTGGATACTTAGAGAAAGTGTATCAAACTGCTAAATCGGTAGAAGC

CCAAACGTTCCACGATGCGATTTGTGCCCTTATCGTAGAAGAGCTGTTTGAATATGCA

GGCAAATGGCGTAATATTCGTGTGCAAGGACCGACAACATTTCTACCATCCTTGACTG

TACAGGTAGCAATGGCAGGTGCCATGTTGATTGGTCTGCATCATCGCATCTGTTATAC

GACGAGCGCTTCGGTCTTAACTGAAGCAGTTAAGCAATCAGATCTTCCTTCAGGTTAT

GACCATCTGTGCCAGTTCGTAATGTCTGGTCAACTTTCCGACTCTGAGAAACTTCTGG

AATCGCTAGAGAATTTCTGGAATGGGATTCAGGAGTGGACAGAACGACACGGATATAT

AGTGGATGTGTCAAAACGCATACCATTTTGAACGATGACCTCTAATAATTGTTAATCA

TGTTGGTTACGTATTTATTAACTTCTCCTAGTATTAGTAATTATCATGGCTGTCATGG

CGCATTAACGGAATAAAGGGTGTGCTTAAATCGGGCCATTTTGCGTAATAAGAAAAAG

GATTAATTATGAGCGAATTGAATTAATAATAAGGTAATAGATTTACATTAGAAAATGA

AAGGGGATTTTATGCGTGAGAATGTTACAGTCTATCCCGGCATTGCCAGTCGGGGATA

TTAAAAAGAGTATAGGTTTTTATTGCGATAAACTAGGTTTCACTTTGGTTCACCATGA

AGATGGATTCGCAGTTCTAATGTGTAATGAGGTTCGGATTCATCTATGGGAGGCAAGT

GATGAAGCTGGCGCTCTCGTAGTAATGATTCACCGGTTTGTACAGGTGCGGAGTCGTT

TATTGCTGGTACTGCTAGTTGCCGCATTGAAGTAGAGGGAATTGATGAATTATATCAA

CATATTAAGCCTTTGGGCATTTTGCACCCCAATACATCATTAAAAGATCAGTGGTGGG

ATGAACGAGACTTTGCAGTAATTGATCCCGACAACAATTTGATTAGCTTTTTTCAACA

AATAAAAAGCTAAAATCTATTATTAATCTGTTCAGCAATCGGGCGCGATTGCTGAATA

AAAGATACGAAGGTGATGGTTTTGAACTTGTTCTTTCTTATCTTGATACATATAGAAA

TAACGTCATTTTTATTTTAGTTGCTGAAAGGTGCGTTGAAGTGTTGGTATGTATGTGT

TTTAAAGTATTGAAAACCCTTAAAATTGGTTGCACAGAAAAACCCCATCTGTTAAAGT

TATAAGTGACTAAACAAATAACTAAATAGATGGGGGTTTCTTTTAATATTATGTGTCC

TAATAGTAGCATTTATTCAGATGAAAAATCAAGGGTTTTAGTGGACAAGACAAAAAGT

GGAAAAGTGAGGCCATGGAGAGAAAAGAAAATCGCTAATGTTGATTACTTTGAACTTC

TGCATATTCTTGAATTTAAAAAGGCTGAAAGAGTAAAAGATTGTGCTGAAATATTAGA

GTATAAACAAAATCGTGAAACAGGCGAAAGAAAGTTGTATCGAGTGTGGTTTTGTAAA

TCCAGGCTTTGTCCAATGTGCAACTGGAGGAGAGCAATGAAACATGGCATTCAGTCAC

AAAAGGTTGTTGCTGAAGTTATTAAACAAAAGCCAACAGTTCGTTGGTTGTTTCTCAC

ATTAACAGTTAAAAATGTTTATGATGGCGAAGAATTAAATAAGAGTTTGTCAGATATG

GCTCAAGGATTTCGCCGAATGATGCAATATAAAAAAATTAATAAAAATCTTGTTGGTT

TTATGCGTGCAACGGAAGTGACAATAAATAATAAAGATAATTCTTATAATCAGCACAT

GCATGTATTGGTATGTGTGGAACCAACTTATTTTAAGAATACAGAAAACTACGTGAAT

CAAAAACAATGGATTCAATTTTGGAAAAAGGCAATGAAATTAGACTATGATCCAAATG

TAAAAGTTCAAATGATTCGACCGAAAAATAAATATAAATCGGATATACAATCGGCAAT

TGACGAAACTGCAAAATATCCTGTAAAGGATACGGATTTTATGACCGATGATGAAGAA

AAGAATTTGAAACGTTTGTCTGATTTGGAGGAAGGTTTACACCGTAAAAGGTTAATCT

CCTATGGTGGTTTGTTAAAAGAAATACATAAAAAATTAAACCTTGATGACACAGAAGA

AGGCGATTTGATTCATACAGATGATGACGAAAAAGCCGATGAAGATGGATTTTCTATT

ATTGCAATGTGGAATTGGGAACGGAAAAATTATTTTATTAAAGAGTAGTTCAACAAAC

GGGCCAGTTTGTTGAAGATTAGATGCTATAATTGTTATTAAAAGGATTGAAGGATGGG
AG"

/label="ACTCCATCTGGATTTGTTCAGAACGCTCGGTTGCCGCCGGGCGTTTTTTA

TCTAGCTAGACCTGACTTCCGCCTGCAGGGCCAGCTCGCGGACGTGCTCATAGTCCAC

GACGCCCGTGATTTTGTAGCCCTGGCCGACGGCCAGCAGGTAGGCCGACAGGCTCATG

CCGGCCGCCGCCGCCTTTTCCTCAATCGCTCTTCGTTCGTCTGGAAGGCAGTACACCT

TGATAGGTGGGCTGCCCTTCCTGGTTGGCTTGGTTTCATCAGCCATCCGCTTGCCCTC

ATCTGTTACGCCGGCGGTAGCCGGCCAGCCTCGCAGAGCAGGATTCCCGTTGAGCACC

GCCAGGTGCGAATAAGGGACAGTGAAGAAGGAACACCCGCTCGCGGGTGGGCCTACTT

CACCTATCCTGCCCGGCTGACGCCGTTGGATACACCAAGGAAAGTCTACACGAACCCT

TTGGCAAAATCCTGTATATCGTGCGAAAAAGGATGGATATACCGAAAAAATCGCTATA

ATGACCCCGAAGCAGGGTTATGCAGCGGAAAAGATCCGTCGACCTGCATACTAGCTGC

TCAAGGTCGCCCGCAACCGGCGCATCAAGCCCGCCGACTAGTGGCCAGGAACCGTAAA

AAGGCCGCGTTGCTGGCGTTTTTCCATAGGCTCCGCCCCCCTGACGAGCATCACAAAA

ATCGACGCTCAAGTCAGAGGTGGCGAAACCCGACAGGACTATAAAGATACCAGGCGTT

TCCCCCTGGAAGCTCCCTCGTGCGCTCTCCTGTTCCGACCCTGCCGCTTACCGGATAC

CTGTCCGCCTTTCTCCCTTCGGGAAGCGTGGCGCTTTCTCATAGCTCACGCTGTAGGT

ATCTCAGTTCGGTGTAGGTCGTTCGCTCCAAGCTGGGCTGTGTGCACGACCCCCCGT

TCAGCCCGACCGCTGCGCCTTATCCGGTAACTATCGTCTTGAGTCCAACCCGGTAAGA

CACGACTTATCGCCACTGGCAGCAGCCACTGGTAACAGGATTAGCAGAGCGAGGTATG

TAGGCGGTGCTACAGAGTTCTTGAAGTGGTGGCCTAACTACGGCTACACTAGAAGAAC

AGTATTTGGTATCTGCGCTCTGCTGAAGCCAGTTACCTTCGGAAAAAGAGTTGGTAGC

TCTTGATCCGGCAAACAAACCACCGCTGGTAGCGGTGGTTTTTTTGTTTGCAAGCAGC

AGATTACGCGCAGAAAAAAAGGATCTCAAGAAGATCCTTTGATCTTTTCTACGGGGTC

TGACGCTCAGTGGAACGAAAACTCACGTTAAGGGATTTTGGTCATGAGATTATCAAAA

AGGATCTTCACCTAGATCCTTTTGGTTCATGTGCAGCTCCATCAGCAAAAGGGGATGA

TAAGTTTATCACCACCGACTATTTGCAACAGTGCCGTTAATGGTTATAATAGCTGAAT

AAGAACGGTGCTCTCCAAATATTCTTATTTAGAAAAGCAAATCTAAAATTATCTGAAA

AGGGAATGAGAATAGTGAATGGACCAATAATAATGACTAGAGAAGAAAGAATGAAGAT

TGTTCATGAAATTAAGGAACGAATATTGGATAAATATGGGGATGATGTTAAGGCTATT

GGTGTTTATGGCTCTCTTGGTCGTCAGACTGATGGGCCCTATTCGGATATTGAGATGA

TGTGTGTCATGTCAACAGAGGAAGCAGAGTTCAGCCATGAATGGACAACCGGTGAGTG

GAAGGTGGAAGTGAATTTTGATAGCGAAGAGATTCTACTAGATTATGCATCTCAGGTG

GAATCAGATTGGCCGCTTACACATGGTCAATTTTTCTCTATTTTGCCGATTTATGATT

CAGGTGGATACTTAGAGAAAGTGTATCAAACTGCTAAATCGGTAGAAGCCCAAACGTT

CCACGATGCGATTTGTGCCCTTATCGTAGAAGAGCTGTTTGAATATGCAGGCAAATGG

CGTAATATTCGTGTGCAAGGACCGACAACATTTCTACCATCCTTGACTGTACAGGTAG

CAATGGCAGGTGCCATGTTGATTGGTCTGCATCATCGCATCTGTTATACGACGAGCGC

TTCGGTCTTAACTGAAGCAGTTAAGCAATCAGATCTTCCTTCAGGTTATGACCATCTG

TGCCAGTTCGTAATGTCTGGTCAACTTTCCGACTCTGAGAAACTTCTGGAATCGCTAG

AGAATTTCTGGAATGGGATTCAGGAGTGGACAGAACGACACGGATATATAGTGGATGT

GTCAAAACGCATACCATTTTGAACGATGACCTCTAATAATTGTTAATCATGTTGGTTA

CGTATTTATTAACTTCTCCTAGTATTAGTAATTATCATGGCTGTCATGGCGCATTAAC

GGAATAAAGGGTGTGCTTAAATCGGGCCATTTTGCGTAATAAGAAAAAGGATTAATTA

TGAGCGAATTGAATTAATAATAAGGTAATAGATTTACATTAGAAAATGAAAGGGGATT

TTATGCGTGAGAATGTTACAGTCTATCCCGGCATTGCCAGTCGGGGATATTAAAAAGA

GTATAGGTTTTTATTGCGATAAACTAGGTTTCACTTTGGTTCACCATGAAGATGGATT

CGCAGTTCTAATGTGTAATGAGGTTCGGATTCATCTATGGGAGGCAAGTGATGAAGCT

GGCGCTCTCGTAGTAATGATTCACCGGTTTGTACAGGTGCGGAGTCGTTTATTGCTGG

TACTGCTAGTTGCCGCATTGAAGTAGAGGGAATTGATGAATTATATCAACATATTAAG

CCTTTGGGCATTTTGCACCCCAATACATCATTAAAAGATCAGTGGTGGGATGAACGAG

ACTTTGCAGTAATTGATCCCGACAACAATTTGATTAGCTTTTTTCAACAAATAAAAAG

CTAAAATCTATTATTAATCTGTTCAGCAATCGGGCGCGATTGCTGAATAAAAGATACG

AAGGTGATGGTTTTGAACTTGTTCTTTCTTATCTTGATACATATAGAAATAACGTCAT

TTTTATTTTAGTTGCTGAAAGGTGCGTTGAAGTGTTGGTATGTATGTGTTTTAAAGTA

TTGAAAACCCTTAAAATTGGTTGCACAGAAAAACCCCATCTGTTAAAGTTATAAGTGA

CTAAACAAATAACTAAATAGATGGGGGTTTCTTTTAATATTATGTGTCCTAATAGTAG

CATTTATTCAGATGAAAAATCAAGGGTTTTAGTGGACAAGACAAAAAGTGGAAAAGTG

AGGCCATGGAGAGAAAAGAAAATCGCTAATGTTGATTACTTTGAACTTCTGCATATTC

TTGAATTTAAAAAGGCTGAAAGAGTAAAAGATTGTGCTGAAATATTAGAGTATAAACA

AAATCGTGAAACAGGCGAAAGAAAGTTGTATCGAGTGTGGTTTTGTAAATCCAGGCTT

TGTCCAATGTGCAACTGGAGGAGAGCAATGAAACATGGCATTCAGTCACAAAAGGTTG

TTGCTGAAGTTATTAAACAAAAGCCAACAGTTCGTTGGTTGTTTCTCACATTAACAGT

TAAAAATGTTTATGATGGCGAAGAATTAAATAAGAGTTTGTCAGATATGGCTCAAGGA

TTTCGCCGAATGATGCAATATAAAAAAATTAATAAAAATCTTGTTGGTTTTATGCGTG

CAACGGAAGTGACAATAAATAATAAAGATAATTCTTATAATCAGCACATGCATGTATT

GGTATGTGTGGAACCAACTTATTTTAAGAATACAGAAAACTACGTGAATCAAAAACAA

TGGATTCAATTTTGGAAAAAGGCAATGAAATTAGACTATGATCCAAATGTAAAAGTTC

AAATGATTCGACCGAAAAATAAATATAAATCGGATATACAATCGGCAATTGACGAAAC

TGCAAAATATCCTGTAAAGGATACGGATTTTATGACCGATGATGAAGAAAAGAATTTG

AAACGTTTGTCTGATTTGGAGGAAGGTTTACACCGTAAAAGGTTAATCTCCTATGGTG

GTTTGTTAAAAGAAATACATAAAAAATTAAACCTTGATGACACAGAAGAAGGCGATTT

GATTCATACAGATGATGACGAAAAAGCCGATGAAGATGGATTTTCTATTATTGCAATG

TGGAATTGGGAACGGAAAAATTATTTTATTAAAGAGTAGTTCAACAAACGGGCCAGTT
TGTTGAAGATTAGATGCTATAATTGTTATTAAAAGGATTGAAGGATGGGAG"
/note="Geneious type: Editing History Replacement"

ORIGIN
1 tctagagtcg acgtccccgg ggcagcccgc ctaatgagcg ggcttttttc acgtcacgcg
61 tccatggaga tctttgtctg caactgaaaa gtttatacct tacctggaac aaatggttga
121 aacatacgag gctaatatcg gcttattagg aatagtccct gtactaataa aatcaggtgg

181 atcagttgat cagtatattt tggacgaagc tcggaaagaa tttggagatg acttgcttaa
241 ttccacaatt aaattaaggg aaagaataaa gctcaagaag aattctagct agacctgact
301 tccgcctgca gggccagctc gcggacgtgc tcatagtcca cgacgcccgt gattttgtag
361 ccctggccga cggccagcag gtaggccgac aggctcatgc cggccgccgc cgccttttcc
421 tcaatcgctc ttcgttcgtc tggaaggcag tacaccttga taggtgggct gcccttcctg
481 gttggcttgg tttcatcagc catccgcttg ccctcatctg ttacgccggc ggtagccggc
541 cagcctcgca gagcaggatt cccgttgagc accgccaggt gcgaataagg gacagtgaag
601 aaggaacacc cgctcgcggg tgggcctact tcacctatcc tgcccggctg acgccgttgg
661 atacaccaag gaaagtctac acgaaccctt tggcaaaatc ctgtatatcg tgcgaaaaag
721 gatggatata ccgaaaaaat cgctataatg accccgaagc agggttatgc agcggaaaag
781 atccgtcgac ctgcatacta gctgctcaag gtcgcccgca accggcgcat caagcccgcc
841 gactagtggc caggaaccgt aaaaaggccg cgttgctggc gtttttccat aggctccgcc
901 cccctgacga gcatcacaaa aatcgacgct caagtcagag gtggcgaaac ccgacaggac
961 tataaagata ccaggcgttt ccccctggaa gctccctcgt gcgctctcct gttccgaccc
1021 tgccgcttac cggatacctg tccgcctttc tcccttcggg aagcgtggcg ctttctcata
1081 gctcacgctg taggtatctc agttcggtgt aggtcgttcg ctccaagctg ggctgtgtgc
1141 acgaaccccc cgttcagccc gaccgctgcg ccttatccgg taactatcgt cttgagtcca
1201 acccggtaag acacgactta tcgccactgg cagcagccac tggtaacagg attagcagag
1261 cgaggtatgt aggcggtgct acagagttct tgaagtggtg gcctaactac ggctacacta
1321 gaagaacagt atttggtatc tgcgctctgc tgaagccagt taccttcgga aaaagagttg
1381 gtagctcttg atccggcaaa caaaccaccg ctggtagcgg tggttttttt gtttgcaagc
1441 agcagattac gcgcagaaaa aaaggatctc aagaagatcc tttgatcttt tctacggggt
1501 ctgacgctca gtggaacgaa aactcacgtt aagggatttt ggtcatgaga ttatcaaaaa
1561 ggatcttcac ctagatcctt ttggttcatg tgcagctcca tcagcaaaag gggatgataa
1621 gtttatcacc accgactatt tgcaacagtg ccgttaatgg ttataatagc tgaataagaa
1681 cggtgctctc caaatattct tatttagaaa agcaaatcta aaattatctg aaaagggaat
1741 gagaatagtg aatggaccaa taataatgac tagagaagaa agaatgaaga ttgttcatga
1801 aattaaggaa cgaatattgg ataaatatgg ggatgatgtt aaggctattg gtgtttatgg
1861 ctctcttggt cgtcagactg atgggcccta ttcggatatt gagatgatgt gtgtcatgtc
1921 aacagaggaa gcagagttca gccatgaatg gacaaccggt gagtggaagg tggaagtgaa
1981 ttttgatagc gaagagattc tactagatta tgcatctcag gtggaatcag attggccgct
2041 tacacatggt caatttttct ctattttgcc gatttatgat tcaggtggat acttagagaa
2101 agtgtatcaa actgctaaat cggtagaagc ccaaacgttc cacgatgcga tttgtgccct
2161 tatcgtagaa gagctgtttg aatatgcagg caaatggcgt aatattcgtg tgcaaggacc
2221 gacaacattt ctaccatcct tgactgtaca ggtagcaatg gcaggtgcca tgttgattgg
2281 tctgcatcat cgcatctgtt atacgacgag cgcttcggtc ttaactgaag cagttaagca
2341 atcagatctt ccttcaggtt atgaccatct gtgccagttc gtaatgtctg gtcaactttc
2401 cgactctgag aaacttctgg aatcgctaga gaatttctgg aatgggattc aggagtggac
2461 agaacgacac ggatatatag tggatgtgtc aaaacgcata ccattttgaa cgatgacctc
2521 taataattgt taatcatgtt ggttacgtat ttattaactt ctcctagtat tagtaattat
2581 catggctgtc atggcgcatt aacggaataa agggtgtgct taaatcgggc cattttgcgt
2641 aataagaaaa aggattaatt atgagcgaat tgaattaata ataaggtaat agatttacat
2701 tagaaaatga aaggggattt tatgcgtgag aatgttacag tctatcccgg cattgccagt
2761 cggggatatt aaaaagagta taggttttta ttgcgataaa ctaggtttca ctttggttca
2821 ccatgaagat ggattcgcag ttctaatgtg taatgaggtt cggattcatc tatgggaggc
2881 aagtgatgaa gctggcgctc tcgtagtaat gattcaccgg tttgtacagg tgcggagtcg
2941 tttattgctg gtactgctag ttgccgcatt gaagtagagg gaattgatga attatatcaa
3001 catattaagc ctttgggcat tttgcacccc aatacatcat taaaagatca gtggtgggat
3061 gaacgagact ttgcagtaat tgatcccgac aacaatttga ttagcttttt tcaacaaata
3121 aaaagctaaa atctattatt aatctgttca gcaatcgggc gcgattgctg aataaaagat
3181 acgaaggtga tggttttgaa cttgttcttt cttatcttga tacatataga aataacgtca
3241 tttttatttt agttgctgaa aggtgcgttg aagtgttggt atgtatgtgt tttaaagtat
3301 tgaaaaccct taaaattggt tgcacagaaa aaccccatct gttaaagtta taagtgacta
3361 aacaaataac taaatagatg gggggtttctt ttaatattat gtgtcctaat agtagcattt
3421 attcagatga aaaatcaagg gttttagtgg acaagacaaa aagtggaaaa gtgaggccat
3481 ggagagaaaa gaaaatcgct aatgttgatt actttgaact tctgcatatt cttgaattta

3541 aaaaggctga aagagtaaaa gattgtgctg aaatattaga gtataaacaa aatcgtgaaa
3601 caggcgaaag aaagttgtat cgagtgtggt tttgtaaatc caggctttgt ccaatgtgca
3661 actggaggag agcaatgaaa catggcattc agtcacaaaa ggttgttgct gaagttatta
3721 aacaaaagcc aacagttcgt tggttgtttc tcacattaac agttaaaaat gtttatgatg
3781 gcgaagaatt aaataagagt ttgtcagata tggctcaagg atttcgccga atgatgcaat
3841 ataaaaaaat taataaaaat cttgttggtt ttatgcgtgc aacggaagtg acaataaata
3901 ataaagataa ttcttataat cagcacatgc atgtattggt atgtgtggaa ccaacttatt
3961 ttaagaatac agaaaactac gtgaatcaaa aacaatggat tcaattttgg aaaaaggcaa
4021 tgaaattaga ctatgatcca aatgtaaaag ttcaaatgat tcgaccgaaa aataaatata
4081 aatcggatat acaatcggca attgacgaaa ctgcaaaata tcctgtaaag gatacggatt
4141 ttatgaccga tgatgaagaa aagaatttga aacgtttgtc tgatttggag gaaggtttac
4201 accgtaaaag gttaatctcc tatggtggtt tgttaaaaga aatacataaa aaattaaacc
4261 ttgatgacac agaagaaggc gatttgattc atacagatga tgacgaaaaa gccgatgaag
4321 atggattttc tattattgca atgtggaatt gggaacggaa aaattatttt attaaagagt
4381 agttcaacaa acgggccagt ttgttgaaga ttagatgcta taattgttat taaaaggatt
4441 gaaggatggg agcgctggtt acctataccc aggtagagcc tgccgacttg aaggatgaaa
4501 ccattttgca tacagaggca ggatgtacgt accgtatcct tttgagcagt atctgaataa
4561 acacggtatt ttttcagacc ctagcctgga attttggagt attgaagcca tcaagcagtg
4621 tgtcatggct gggctgggta ttgcgttgct cccgctagta acggtacaaa atgagctgcg
4681 agaaggtaaa atggcgcgtt tagcctggga tgacagtgaa cagcaggtgg ctactcaggt
4741 cgcttatcac acgaaaaagt ggaaatcccc ggctcttagc gaatttttac agatcgttga
4801 gcagcatgta acacattggc gtgcatgagt tcaagccatc acggcatatg cttatacaaa
4861 taatattttt ccttttcgcc tattcactca acattcgccc acaacctgca tacatatgta
4921 tggaatgatt gattgtatgg aggaggttca agaatggcat taagtcataa aaatcgtaga
4981 gagattatta ccaaagcgat ctgtggtaaa ggtcgcagat tctctaccgt aacccatacc
5041 gtaactccgc cgaataatcc gacgagcatt ttaggggcat ggattattaa ccaccagtat
5101 gaagctgtgg cggcggggga cggcattgag gtcgtcggga cgtatgatat caatatttgg
5161 tactcatacg ataaaaactc gcagaccgat gttgccaagg aaacggtgtc gtacgtagaa
5221 aatgtgccgc tctcgtatct tgatccgaag caccgggcgt ctacagtgga agtatccgcc
5281 gaagctacac aggagccgag ttgcgtcgag gccagtgtgt cttctggagg tggcagcgta
5341 atgatccggg tcgagcggga atttgcggtg gagctggtgg cggaaacgaa gattgttgta
5401 gaagtattcc cgaatggcag cagcgatgat tttgacaaag actttgattt tggagcggaa
5461 gagggggact atgaggagct cgaccccgac ctcattgacg acgagctcgg tggaggtggt
5521 ggaagtggtg gaggtggtgg aggtttgtct agaaaagaga aggaagctaa gcccgactgg
5581 attcgtatta aacttacgac aggcgataac tatcaagaaa tcaagagcat gatgcgttcc
5641 aagacattac ataccgtatg tgaggaggca cgatgcccta atatatatga atgctgggcc
5701 aataggacag ctacatttat gattttgggt gatatttgta cgcgtgcgtg tcgtttttgc
5761 gcagtaaata cgggattgcc tacggaactt gacttgcagg agcctgatcg cgtcgcggaa
5821 gcggctggga acatgaattt gaagcactgt gtcgtaacca gtgtggctcg cgacgattta
5881 aaagatgggg gcgctactat atttgcagaa acggttcgtg cgattcgcgg ccggttacct
5941 ttgtgcagtg tagaagtatt gattcccgat tttatgggcg atattgaatc gctgcgaatc
6001 gtcatggatg ccaaaccaga tattctgaat cataatattg aaacggtgga gcgcatgtcc
6061 gacaaggttc gggctaaagc gaaataccgt cgctctctgg agctgcttca acgtgcgaag
6121 caattaaatc cggctattcc aacgaaatca agcatcatgc taggtgtcgg agaagagtgg
6181 gacgagatct tacaagccat ggatgatctg cgtaaagtcg attgtgacat tctgacaatt
6241 ggtcaatatt tgcagccgtc cgagaaacat ttatatgtgc aaaaatatta tccaccggaa
6301 gattttgcga agttaaagga agagggatta cagcggggat tcagccacgt tgaatctgga
6361 ccgttggtcc gcagctccta ccaagcgcat gagcaggtga agtcagccgc tcagcataag
6421 gaagaaagcg ccctttcgca ggctcatcat caccaccacc attaa

//
